# Utilization of a Chelating Bis[(dialkylamino)cyclopropenimine] to Isolate a Series of Heavier Zero‐Valent Group 14 Tetracarbonyl Iron Complexes

**DOI:** 10.1002/chem.202501324

**Published:** 2025-04-21

**Authors:** Simone V. Hirmer, Shicheng Dong, Sebastian Stigler, Arseni Kostenko, John A. Kelly, Zihan Zhang, Karsten Meyer, Jun Zhu, Shigeyoshi Inoue

**Affiliations:** ^1^ TUM School of Natural Sciences Department of Chemistry Catalysis Research Center and Institute of Silicon Chemistry Technische Universität München Lichtenbergstr. 4 85748 Garching bei München Germany; ^2^ State Key Laboratory of Physical Chemistry of Solid Surfaces Fujian Provincial Key Laboratory of Theoretical and Computational Chemistry College of Chemistry and Chemical Engineering Xiamen University Xiamen 361005 China; ^3^ Erlangen‐Nürnberg (FAU) Department für Chemie und Pharmazie Friedrich‐Alexander‐Universität Anorganische Chemie, Egerlandstr. 1 91059 Erlangen Germany; ^4^ School of Science and Engineering The Chinese University of Hong Kong Shenzhen, No. 2001 Longxiang Blvd., Longgang Dist. Shenzhen Guangdong 518172 China

**Keywords:** carbonyl iron complexes, germanium, lead, tetrylone, tin

## Abstract

We report on the utilization of the ethylene‐bridged bis[(dialkylamino)cyclopropenimine] (bisCPI) ligand, **L^CPI^**, to give access to new main‐group E(II) halide complexes (E = Ge, Sn, Pb; **1**, **2**, **3**). Subsequent reduction with Collman's reagent (Na_2_Fe(CO)_4_ • dioxane) enables the isolation of a series of zero‐valent tetrylone‐tetracarbonyl iron complexes, (**L^CPI^**)E(Fe(CO)_4_ (E = Ge (**4**), Sn (**5**), Pb (**6**)). Compounds **4 – 6** were reacted further with iron pentacarbonyl to yield the bis‐tetracarbonyl iron complexes (**L^CPI^**)E[(Fe(CO)_4_]_2_ (E = Ge (**7**), Sn (**8**), Pb (**9**)). The electronic structure of these complexes was studied by ^57^Fe Mössbauer spectroscopy and computationally by density functional theory calculations.

## Graphical abstract

The bis‐cyclopropenimine ligand was used to isolate tetracarbonyl iron‐stabilized tetrylones (Ge^0^, Sn^0^, Pb^0^). The strong donating abilities of tetrylones were further proven by reaction with Fe(CO)_5_ and Fe_2_(CO)_9_ to access the bis‐Fe(CO)_4_ complexes. The complexes were structurally characterized by NMR‐ and Mössbauer spectroscopy and X‐ray crystallography, and their bonding situations were investigated computationally using density functional theory.

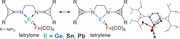

## 1 Introduction

**Figure 1:**
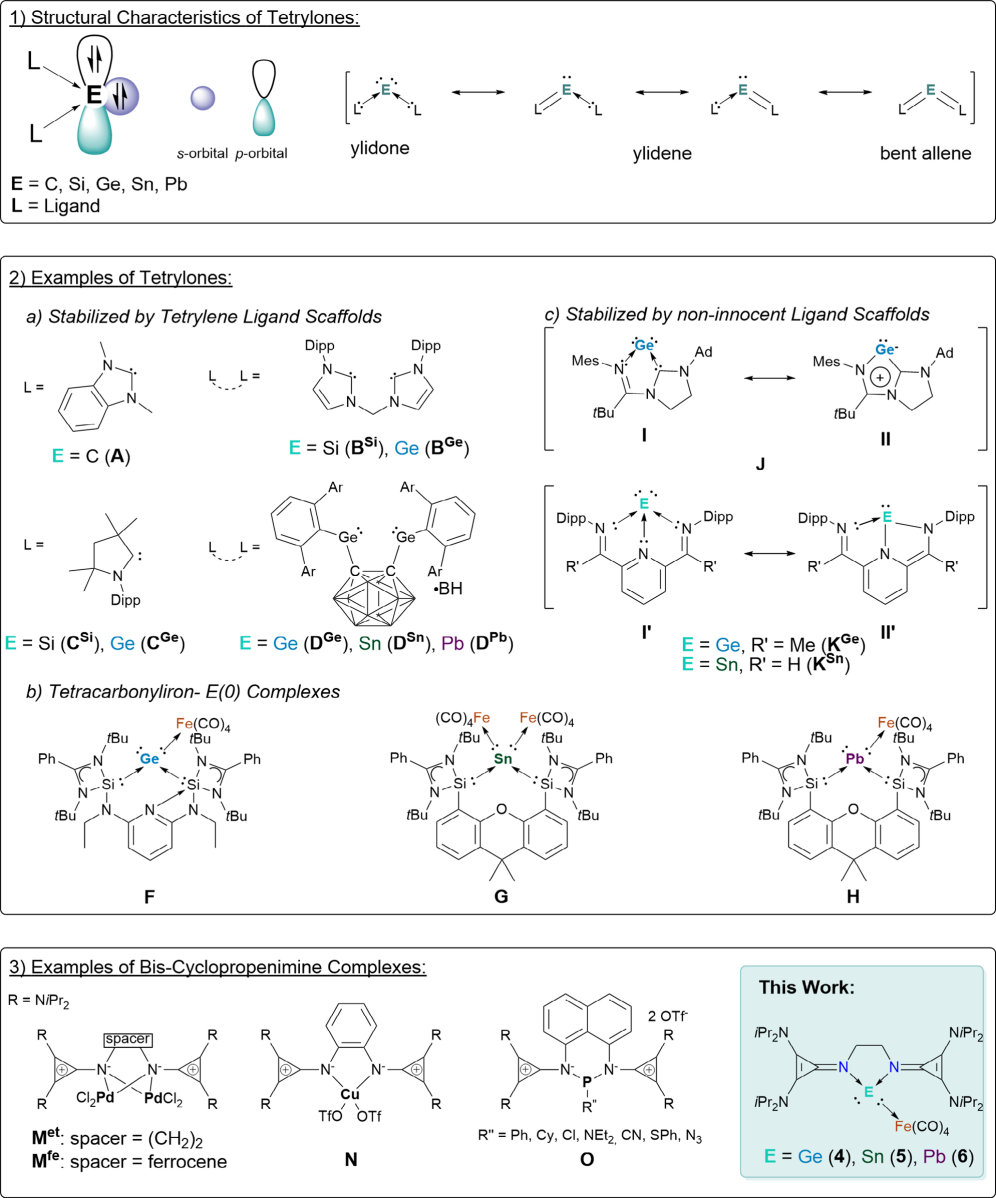
a) Structural characteristics of tetrylones and their mesomeric features, b) various examples of tetrylones stabilized by 1) NHC (**A**, **B** ^**Si**^, **B** ^Ge^), cAAC (**C** ^**Si**^, **C** ^**Ge**^) and bis‐germylene (**D** ^**Ge**^, **D** ^**Sn**^, **D** ^**Pb**^, Ar = 2,4,6‐ triisopropylphenyl), 2) examples of tetracarbonyl iron E(0) complexes stabilized by bisNHSi (**F**, **G**, **H**), and 3) non‐innocent ligands such as the imino‐N‐Heterocyclic carbene germylone/germylene **J** and the three‐fold coordinated diiminopyridene stannylones **K** ^**Sn**^ and germylone **K** ^**Ge**^ (Dipp  =  3,6‐diisopropylphenyl), and ca) examples of bis‐cyclopropenimine complexes with Pd (**M** ^**et**^, **M** ^**fe**^), Cu (M) and dicationic phosphor species **O** and herein presented bis‐cyclopropenimine stabilized germylone (**4**) stannylone (**5**) and plumbylone (**6**) tetracarbonyl iron complexes.

Tetrylones are a class of compounds where the central tetrel element is in the formal oxidation state 0, with the general formula L_2_E (E = C ‐ Pb, L = ligand).^[^ ^1^ ^]^ The four valence electrons of the tetrel center form two lone pairs of electrons and the ligands datively bond to the empty p‐orbitals. Depending on the nature of the ligand, the species can be described as having a ylidone, ylidene, or bent allene character. (Figure 1a).^[^ ^2^ ^]^

The first example of such a compound was the phosphine‐stabilized carbone (PPh_3_)_2_C reported in 1961 by Ramirez and coworkers, which was initially described with electron‐sharing bonds (e.g., Ph_3_P = C = PPh_3_) or as a double Wittig‐type ylide (Ph_3_P^+^‐C^2−^‐P^+^Ph_3_).^[^ ^3^ ^]^ However, calculations by Frenking et al. revealed that the more precise characterization is that of a carbone species.^[^ ^4^ ^]^ Since then, there has been a scarcity of reports on tetrylones; only in the last decade has their chemistry resurged with the isolation of a variety of carbones (C^0^), silylones (Si^0^), and germylones (Ge^0^).^[^ ^1b^ ^]^ Their heavier analogs, stannylones (Sn^0^) and plumbylones (Pb^0^) are rarer due to their lower stability, making their isolation difficult.^[^ ^5^ ^]^

**Figure 2:**
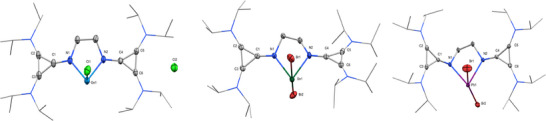
Molecular structures of **1** (left), **2** (middle), and **3** (right) in the solid state (hydrogen atoms and solvent molecules are removed for clarity, ellipsoids set at 50% probability level).

Some noteworthy examples are depicted in Figure 1, grouped by ligand class (e.g., tetrylene‐stabilized tetrylones and non‐innocent ligand scaffolds). By exploiting the donor capabilities of carbenes (e.g., *N*‐heterocyclic carbenes (NHCs) or cyclic (alkyl) amino carbenes (cAACs)) and their heavier analogs, a plethora of tetrylones could be isolated. The first example was the carbone **A** (Figures 1b and 2a), where the central carbon atom is stabilized by two NHC ligands, synthesized in 2008 by Bertrand and coworkers.^[^ ^6^ ^]^ In 2013, the first examples of silylones and germylones (**B^Si^** and **B^Ge^**) were reported and stabilized with bisNHC ligands.^[^ ^7^ ^]^ In the same year, by utilizing cAACs, which are known to possess superior σ‐donor and π‐acceptor abilities compared to NHCs,^[^ ^8^ ^]^ the silylone **C^Si^**, and germylone, **C^Ge^** were reported by the groups of Roesky, Frenking and Stalke.^[^ ^9^ ^]^ Recently, the groups of Zhao and Mo synthesized the first plumbylone **D^Pb^**, which is stabilized by a bis‐germylene ligand. Transmetallation rection of **D^Pb^** with GeCl_2_ • dioxane formed the bis‐germylene germylone **D^Ge^**.^[^ ^10^ ^]^ Later, they isolated the stannylone **D^Sn^** using the same ligand framework.^[^ ^11^ ^]^

With the use of chelating bis‐*N*‐heterocyclic silylenes (bisNHSi), Driess and coworkers have developed a variety of bidentate ligands with different spacers to establish a wealth of tetrylones, which have recently been reviewed (Figures 1b and 2b).^[^ ^12^ ^]^ Among them are the germylone‐tetracarbonyl iron complex **F** with a pyridine spacer^[^ ^13^ ^]^ and the Sn^0^‐[Fe(CO)_4_]_2_ complex **G** with a xanthene spacer.^[^ ^14^ ^]^ Those complexes highlight the strong σ‐donor abilities of tetrylones, as they can coordinate up to two Lewis acids due to their two chemically active lone pairs of electrons. Isolation of tetracarbonyl iron E(0) complexes can be achieved by the addition of Fe_2_(CO)_9_ to the isolated tetrylones, such as with complex **G**, or by the reduction of E(II) halides with Collman's reagent (Na_2_Fe(CO)_4_ • dioxane).^[^ ^14^ ^]^ The first monoatomic two‐coordinated stannylone was isolated via a reaction of **G** with KC_8_.^[^ ^14^ ^]^ However, this synthetic route is not feasible for isolating the Lewis acid‐free plumbylone analog of **H**.^[^ ^15^ ^]^

The general ligand motif used in stabilizing tetrylones contains tetrylenes (NHC, cAAC, silylenes, and germylenes), with only a few exceptions of isolated tetrylones stabilized by a different donor moiety.^[^ ^16^ ^]^ Examples of redox non‐innocent ligand scaffolds include the imino‐*N*‐heterocyclic carbenes (**J**) or diiminopyridenes (**K^Ge/Sn^**, Figures 1b and 2c).^[^ ^16a–c^ ^]^

However, it should be noted that for both examples, the complexes can be viewed as tetrylones or tetrylenes (mesomeric structures **I** versus **II** E^0^ versus E^II^). Hence, **J** can be viewed as tetrylone or mesoionic germylene, structures **I** versus **II**, respectively.^[^ ^16a^ ^]^ Apart from the previously mentioned carbodiphosphorane ((PPh_3_)_2_C),^[^ ^3^ ^]^ examples of tetrylones stabilized by group 15‐based ligands are limited only to two examples (**K^Ge^** and **K^Sn^**) by the groups of Nikonov as well as Fischer and Flock with three‐fold coordination by a 2,6‐diiminopyridene ligand scaffold ([dimpyrGe^0^] dimpyr = 2,6‐(ArN═CMe)_2_NC_5_H_3_);^[^ ^16b^ ^]^ and [dimpySn^0^] complex (dimpy = (2,6‐[ArN = CH]_2_(NC_5_H_3_))  with Ar = 2,6‐*i*Pr_2_C_6_H_3_^[^ ^16c^ ^]^), with mesomeric stabilization between oxidation states 0 and II (Figure 1b) **I** **'** and **I** **''**.^[^ ^16b–c^ ^]^

Intrigued by this observation, we wanted to expand the tetrylone chemistry by incorporating a nitrogen‐donor ligand scaffold. Our group successfully utilized *N*‐heterocyclic imino ligands (NHI) to stabilize low oxidation state main‐group complexes and was interested in using a bidentate system with this moiety.^[^ ^17^ ^]^

**Figure 3:**
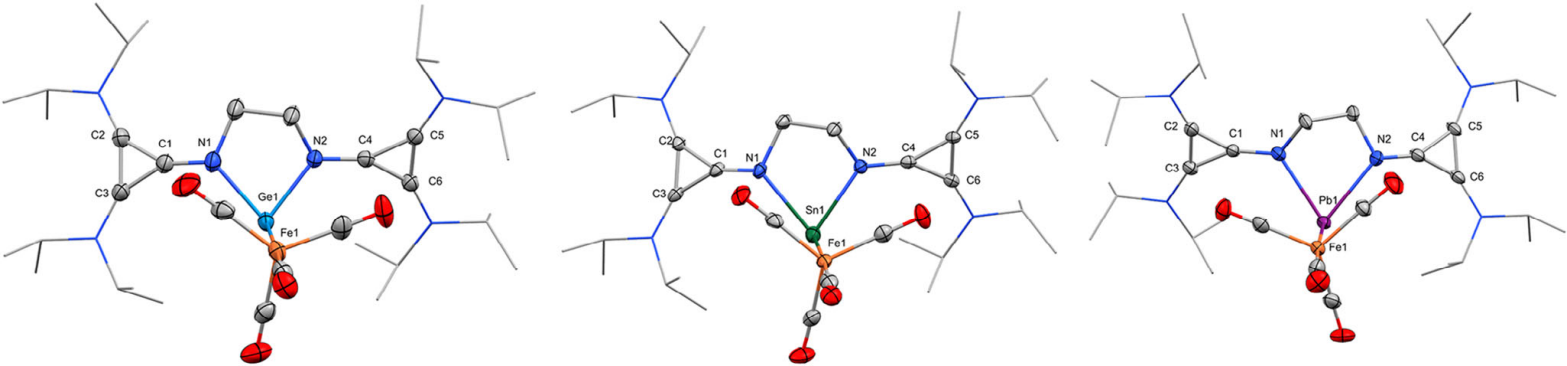
Molecular structures of **4** (left), **5** (middle), and **6** (right) in the solid state (hydrogen atoms and solvent molecules are removed for clarity, ellipsoids set at 50% probability level).

The ethylene‐bridged bis‐(cyclopropenimine) (bisCPI) ligand **L^CPI^**, reported by the Alcarazo group in 2010, exhibits strong electron donor ability via its imino functionalization.^[^ ^18^ ^]^ Additionally, the cyclopropenyl moieties in the scaffold can be viewed as an electron reservoir, forming aromatic cyclopropenyl cations when donating electron density toward the central element, offering high donation strength. This increases the complexation strength due to the gain in thermodynamic stability of the aromatic side arms. Due to resonance stabilization, the negative charge resides on the imino nitrogen, which possesses two lone pairs and can act as a four‐electron donor.^[^ ^18^, ^19^ ^]^ This was highlighted by the isolation of the bimetallic palladium complex **M^et^**, which has not been observed for similar bis‐*N*‐heterocyclic imines (bisNHI).^[^ ^20^ ^]^ Later, this unique characteristic of the dimeric palladium complex was also confirmed by the group of Tamm with the isolation of **M^fc^** by utilizing a ferrocene‐bridged bisCPI ligand (Figures 1c and 3).^[^ ^19^ ^]^

Furthermore, Alcarazo et al. could show the potential of the aryl‐bridged bisCPI ligand system (phenyl and naphthalene bridge) by isolating the electron‐rich dicationic P(III) complexes (**O**) that could be oxidized to P(IV) species.^[^ ^21^ ^]^ By utilizing a phenyl‐bridged bisCPI scaffold, the Lee group isolated a monomeric Cu(II) complex (**N**), which was used as a catalyst for C‐C cross‐coupling.^[^ ^22^ ^]^ However, despite the unique properties of this ligand design, examples of metal complexes stabilized by bisCPI ligands, to the best of our knowledge, are limited to the three mentioned complexes (**M^et^**, **M^fc^**, and **N**). Meanwhile, main‐group compounds with bisCPI ligands are limited to phosphorus complexes (e.g., **O**) stabilized by aryl‐bridged bisCPI.^[^ ^21^ ^]^

It is worth noting that related mono‐cyclopropenimines have also been used as ligands for metal complexes. So far, four examples of rhodium, gold, boron, and thallium have been reported.^[^ ^18^, ^23^ ^]^

Our group has reported on bisNHI low‐valent main‐group complexes and tetryliumylidenes (E = Si, Ge, Sn)^[^ ^17^, ^24^ ^]^. Previous attempts to further reduce these cationic E(II) compounds were unsuccessful. Hence, we were keen to exploit the superior donor abilities of the chelating **L^CPI^** ligand in main‐group chemistry. Herein, we report on the isolation and characterization of a series of E(II) precursors (E = Ge **1**, Sn **2**, and Pb **3**) as the first examples of the group 14 elements being stabilized by a bisCPI ligand scaffold. Furthermore, we reduced **1**, **2**, and **3** with Collman's reagent (Na_2_Fe(CO)_4_ • dioxane) and isolated a series of tetrylone‐Fe(CO)_4_ complexes (E = Ge **4**, Sn **5**, and Pb **6**), which were reacted further with iron pentacarbonyl (Fe(CO)_5_) to form the bis‐tetracarbonyl iron tetrylones (E = Ge **7**, Sn **8**, and Pb **9**).

## 2 Results and Discussion

### 2.1 Synthesis of Precursors

**Scheme 1:**
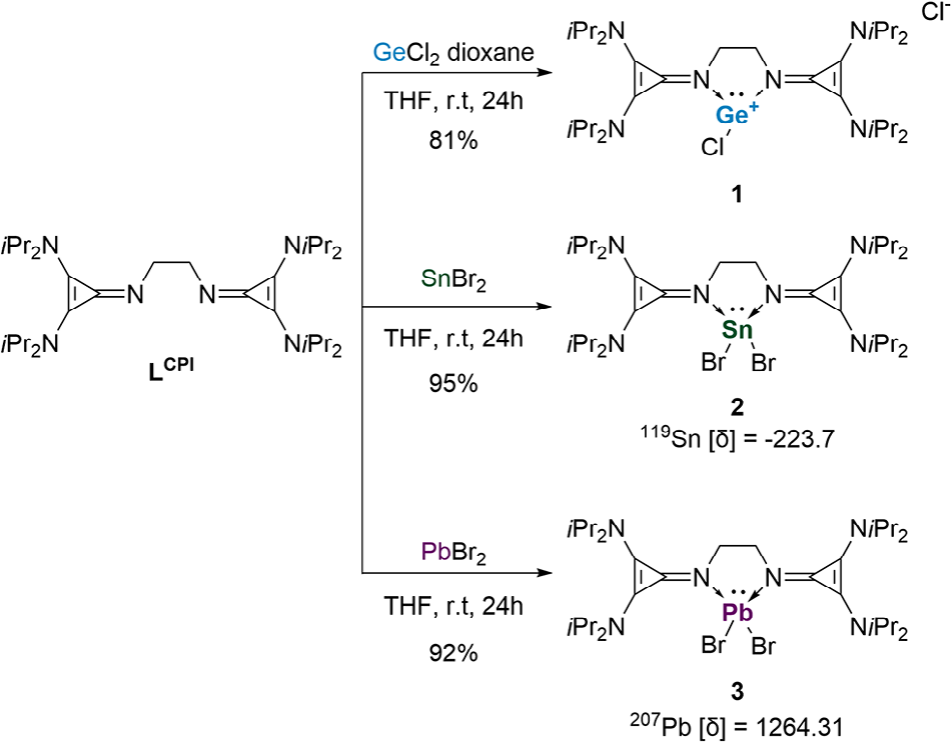
Synthesis of the precursors **1**, **2**, and **3** by adding **L^CPI^** c_2_ (EX_2_ = GeCl_2_ dioxane, SnBr_2_, or PbBr_2_ for **1**, **2**, and **3**, respectively) in THF for 24 h at room temperature (r.t.).

The precursors [(bisCPI)EX_2_] (E = Ge (**1**), Sn (**2**), Pb (**3**)) were synthesized by treating **L^CPI^** with an equimolar ratio of the corresponding tetrel halide (GeCl_2_ • dioxane, SnBr_2_ or PbBr_2_) in THF at room temperature. (Scheme 1)

Compounds **1**, **2**, and **3** were isolated as white solids in moderate to good yields. The ^1^H NMR of **1**, **2**, and **3** are very similar, displaying only the three expected signals for the isopropyl group and the ethylene bridge, indicating a symmetric ligand environment. For compound **2**, the ^119^Sn NMR signal at ‐160.95 ppm is comparable to known Sn(II) complexes with a similar ligand scaffold, such as the bisNHI tin complexes.^[^ ^17a–c^ ^]^ The ^207^Pb NMR signal was detected at δ = 1264.31 ppm for compound **3**, similar to the PbBr_2_ precursor of **D^Pb^** (1585.2 ppm). SC‐XRD quality crystals of **1** and **3** were grown from a saturated toluene solution with slow diffusion of pentane, and **2** was grown from a saturated THF solution. The molecular structure of **1** (Figure 2) is that of a chloro‐germyliumylidene, where the central germanium cation adopts a trigonal‐pyramidal structure with an angle of 82.75° for N1‐Ge‐N2 and 94.19° for Cl1‐Ge‐N, indicating a lone pair of electrons at the Ge atom. This structure is comparable to bisNHI chloro‐tetryliumylidenes synthesized by our group.^[^ ^17^, ^25^ ^]^ One chloride is directly bound to the Ge center, whereas the other is separated from the Ge‐cation at a distance of 7.07 Å. A slight distortion of one of the *iPr* groups reveals a weak (CH_3_)_2_C‐H···Cl^−^ interaction with a distance of 2.724 Å.

Contrary to **1**, in the solid state, compounds **2** and **3** adopt a seesaw geometry (Figure 2), with the bromides as axial ligands in an almost linear fashion (Br1‐Sn1‐Br2 = 172.14° for **2** and Br1‐Pb1‐Br2 = 165.12° for **3**). Much like **1**, the imino‐nitrogen bound five‐membered ring is highly distorted. The structural difference between compounds **1** and **2** / **3** (separated counterion) is similar to that reported by Driess et al. when utilizing their bisNHSi xanthene ligand.^[^ ^13^, ^14^, ^15^ ^]^

Notably, the N‐E bond lengths of compounds **1** and **2** are slightly shorter than related bisNHI tetryliumylidenes, indicating a stronger N‐E bond.^[^ ^17^, ^26^ ^]^ However, the N‐E bond in complexes **1** and **2** are longer compared to the covalent imino‐E bonds for NHI germylenes and stannylenes (E  =  Ge, Sn;, e.g., (I*^t^* BuN)_2_E:, ^Mes^Ter(NHI)Sn:, Dipp(*^i^* Pr_3_Si)N(IDippN)Sn:).^[^ ^27^ ^]^

To date, no bisNHI lead complexes have been reported. The N‐Pb bond in complex **3** (average 2.33 Å) falls between other reported N‐Pb^II^ bonds. It is significantly shorter than the dicationic lead tetramethyl ethylenediamine complex [Pb(NMe_2_)_2_(CH^2^)_2_]I_2_ (2.63 Å),^[^ ^28^ ^]^ but longer than covalent N‐Pb bonds in plumbylenes (2.19‐2.29 Å).^[^ ^29^ ^]^ This intermediate bond length suggests a dative bonding situation in complex **3**.

One key aspect of **L^CPI^** is the electron‐rich cyclopropenyl moiety, which can donate substantial electron density upon aromatising. This is evidenced by examining the bond lengths within the ring and the exocyclic amino group. When comparing the elongation of the average C═N(imino) bond lengths in **1** (1.34 Å) to the value of the free ligand (1.289 Å), this indicates some loss of multiple bond character in the C═N(imino) bonds of complex **1**. Furthermore, an elongation of the C═C bonds in the cyclopropenyl moiety of **1** occurs (average C2‐C3 & C5‐C6 bond lengths for **1**  =  1.387 Å) compared to C2‐C3 in **L^CPI^** = 1.366 Å. The formerly C‐C single bonds in the cyclopropenyl ring of **1** are shortened compared to **L^CPI^** (average C2‐C1 and C3‐C1 bond lengths for **1**  =  1.394 Å versus 1.419 Å for **L^CPI^**). The same trend is observed for **2** and **3** (see Table S1).

**Scheme 2:**
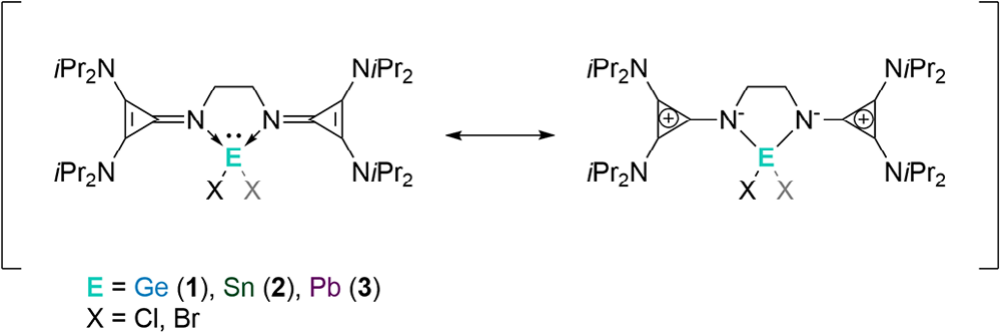
Generalized resonance structures of the precursor complexes **1**, **2**, and **3** whereby E = Ge; X = Cl for **1** and E = Sn and Pb and X = Br for **2** and **3**, respectively.

We also looked into the aromaticity computationally and calculated the Harmonic Oscillator Model of Aromaticity (HOMA) values of the cyclopropenyl moieties to unambiguously prove the aromaticity of this moiety.^[^ ^30^ ^]^ Compounds **1**, **2**, and **3** show an averaged HOMA value of the two cyclopropenyl moieties of 0.99, 0.98, and 0.97, respectively. The free ligand system **L^CPI^** can be viewed as less aromatic (HOMA value of the cyclopropenylring = 0.80), attributed to the more pronounced localization of electrons in the cycloproenyl ring and alternating double and single bonds. This demonstrates a highly aromatic system with delocalized electrons over the ligand, which gives rise to the mesomeric structures of **1**, **2**, and **3** (Scheme 2). This was further confirmed theoretically by the electron density of delocalized bonds (EDDB) (Table S8).^[^ ^31^ ^]^ The values of **L^CPI^** are lower (average of 0.837 over both rings) than those of **1**, **2**, and **3**, with 1.257, 1.222, and 1.189, respectively. A larger EDDB value indicates a more significant aromaticity.

These findings align with the previously reported complexes with cyclopropenimine ligands (*vide supra*).^[^ ^18^, ^21^ ^]^ When **L^CPI^** coordinates with the E(II) center, the electron donation of the imino nitrogen atoms is balanced by the 2‐π electron delocalized system of the electron‐rich cyclopropenyl moieties.

Upon complexation of the imino‐*N* to the tetrel center, the electron deficiency is compensated by the cyclopropenyl rings, which gain aromaticity,^[^ ^18^, ^21^ ^]^ giving rise to the mesomeric structures of **1**, **2**, and **3** (Scheme 2).

### 2.2 Reduction

Attempts to access the desired tetrylone compounds via the reduction of compounds **1** – **3** with KC_8_ were unsuccessful. A metallic precipitate formed, and the free ligand **L^CPI^** was identified as the sole product by ^1^H NMR spectroscopy. This demonstrates that tetrylones are highly reactive species demanding stabilization beyond that provided by the **L^CPI^** ligand scaffold despite its unique ability to provide aromatic stabilization through the cyclopropenyl moieties. Hence, we switched to Collman's reagent, which has been proven successful for synthesizing various tetrylones due to additional Lewis acid stabilization.^[^ ^13^, ^14^, ^15^ ^]^

**Scheme 3:**
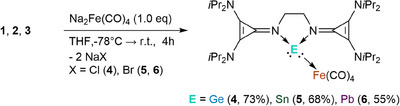
The reaction of compounds **1**–**3** with Collman's reagent (Na_2_Fe(CO)_4_ • 1.5 dioxane), yielded the low‐valent tetrylone‐tetracarbonyl iron complexes **4**, **5**, and **6**.

Treating a suspension of compounds **1**, **2**, or **3** with one equivalent of Collman's reagent resulted in a rapid color change from colorless to dark orange to red in all cases. After workup, germylone **4**, stannylone **5**, and plumbylone **6** Fe(CO)_4_ complexes were isolated in good yields. (Scheme 3).

Complexes **4** – **6** all share similar ^1^H NMR spectra. Comparing them to their respective precursors (**1** – **3**), the proton signals are high‐field shifted, and the formerly singlet signal of the ethylene bridge for compounds **1**, **2**, and **3** is split into two multiplets. The signal for the methyl groups, formerly a doublet, broadens, implying an asymmetric ligand environment.

The ^119^Sn NMR of **5** displays a downfield shift from ‐116 ppm (for **2**) to 899 ppm, which is significantly changed compared to **D^Sn^** (*δ* =  ‐199.96 ppm), **G** (*δ* =  7.3 ppm), and **J^Sn^** (*δ* =  64 ppm), respectively.^[^ ^11^, ^14^, ^16^ ^]^ This indicates a less shielded Sn(0) center for complex **5**. This is due to the lower coordination number of the Sn center in **5** and electron donation to the Lewis acidic Fe(CO)_4_ moiety.

For compound **6**, a signal was observed in the ^207^Pb NMR spectrum at 6542.7 ppm, significantly downfield shifted compared to the precursor **3** (*δ* =  1264.31 ppm), **D^Pb^** (*δ* =  1637.6 ppm) and **H** (*δ* =  2238.9 ppm).^[^ ^10^, ^15^ ^]^ This indicates the stronger electron donation of the NHSi and germylene‐based ligand scaffolds to the central tetrel element compared to the ligand **L^CPI^**.

The solid‐state structures of the tetrylone‐Fe(CO)_4_ complexes **4** – **6** show a distorted trigonal‐pyramidal coordination sphere around the Ge, Sn, and Pb atoms, with the lone pair of electrons indicated at the apex of the central tetrel element (Figure 3). The second electron lone pair is coordinated to the Fe(CO)_4_ moiety. The E(1)‐Fe(1) distances of 2.5856 Å, 2.6958 Å, and 2.7298 Å for **4**, **5**, and **6**, respectively, are within comparable range to the reported distances in the complexes **F**, **G**, and **H**.^[^ ^13^, ^14^, ^15^ ^]^

The average imino‐N(1,2)‐E(1) bond length in **4** – **6** are slightly elongated compared to their respective precursors **1**, **2**, and **3**, which is consistent with the decreasing N(1)‐E(1)‐N(2) angles with 79.59°, 74.94°, 73.12° for **4**, **5**, and **6**, respectively.

The ligand's versatility is reflected when investigating the **4**, **5**, and **6** bond lengths. For instance, in the Ge(0) complex **4**, the average C═N(imino) bond length (1.322 Å) is shortened compared to its precursor compound **1** (1.340 Å) but still longer than in the free ligand **L^CPI^** with 1.289 Å. The N‐Ge bond in **4** is similar to the imine nitrogen Ge bond of **J^Ge^** (2.047 Å and 2.306 Å) but longer than the pyridine N‐Ge bond in **J^Ge^** (1.898 Å) and the imino‐*N*‐Heterocyclic carbene germylone/germylene **I** (1.968 Å). For stannylone **5**, the N‐Sn bonds (2.252 Å) are shorter than the imino N‐Sn bonds of the three‐coordinate **J^Sn^** (2.397 Å and 2.315 Å). However, it is slightly longer compared to the covalent N(pyridine)‐Sn bond (2.122 Å) in **J^Sn^** and NHI stannylones (N‐Sn 2.041^[^ ^27b^ ^]^ and 2.14^[^ ^27a^ ^]^). For the cyclopropenyl moieties, a similar trend, as discussed for the precursors (*vide supra*), can be detected in the solid‐state of compounds **4**, **5**, and **6**. The alternating double and single bonds in the cyclopropenyl rings of **L^CPI^** are uniform, indicating aromatization (see Table S2).

This was also confirmed by the average HOMA values for the cyclopropenyl rings in **4**, **5**, and **6** with 0.97, 0.97, and 0.96, as well as the calculated EDDB values. The average value over the two cyclopropenyl rings is 1.170 for **4**, 1.203 for **5**, and 1.162 for **6**, slightly lower than their respective precursors **1** – **3**. The IR stretching vibrations of CO for **4** (*ṽ_CO_*  =  1948, 1905, 1827 cm^−1^), **5** (*ṽ_CO_*  =  1948, 1905, 1860, 1825 cm^−1^), and **6** (*ṽ_CO_*  =  1950, 1866, 1849, 1823 cm^−1^) are significantly red‐shifted compared to free CO (*ṽ_CO_*  =  2143 cm^−1^)^[^ ^32^ ^]^ and Fe(CO)_5_ (*ṽ_CO_*  =  2034, 2013 cm^−1^)^[^ ^33^ ^]^ but is within comparable range as reported complexes **F** (*ṽ_CO_*  =  1969, 1886, 1865, 1830 cm^−1^), **G** (*ṽ_CO_*  =  1962, 1881, 1834 cm^−1^), and **H** (*ṽ_CO_*  =  1951, 1869, 1848, 1831 cm^−1^).

### 2.3 Further Reactivity

When attempting to synthesize compounds **4**, **5**, and **6** at room temperature, in the case of tin, we detected a mixture of **5** and an unknown species in the ^1^H, ^13^C, and ^119^Sn NMR. With SC‐XRD, we could identify the stannylone [Fe(CO)_4_]_2_ complex **8**, with two Lewis acids coordinated to the Sn center.

**Scheme 4:**
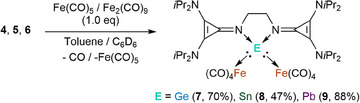
Synthesis of **7**, **8**, and **9** starting from 4, 5, and 6 with an additional equivalent of Fe(CO)_5_ (in the case of compounds **4** and **5**) and Fe_2_(CO)_9_ for compound **6**.

The ^119^Sn NMR signal for compound **8** is at 595 ppm, which is high‐field shifted compared to **5** but downfield shifted to **G** (*δ* =  7.3 ppm). Monitoring compound **5** at elevated temperatures (80 °C) resulted in the formation of compound **8** and unknown insoluble side products. Most likely, compound **5** partly decomposes, as seen for the formation of complex **G**,^[^ ^14^ ^]^, and forms the thermodynamically favored product **8**. To increase the yield and selectivity for compound **8**, the synthesis was optimized by adding one equivalent Fe(CO)_5_ to compound **5** (Scheme 4).

**Figure 4:**
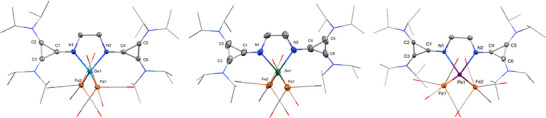
Molecular structures of **7** (left), **8** (middle), and **9** (right) in the solid‐state (hydrogen atoms and solvent molecules are removed for clarity, ellipsoids set at 50% probability level).

The germylone **4** seems more stable than **5** and **6**, as heating to 100 °C for several days showed no conversion to a bis‐tetracarbonyl iron analog. However, we could isolate compound **7** when adding one equivalent Fe(CO)_5_ and heating it to 80 °C for 72 h to ensure complete conversion. Compound **7** was identified with SC‐XRD and exhibited a solid structure similar to compound **8**, with both lone pairs of electrons coordinated to Fe(CO)_4_ units and the Ge and Sn center adopting a distorted tetrahedral geometry (Figure 4).

In the case of the plumbylone **6**, the solid is stable over several weeks at ‐33 °C, however in solution (THF, C_6_D_6_, and toluene) at room temperature after 24 – 48 h, the compound decomposes, depositing a metallic precipitate (most likely elemental lead) and the formation of the Bis‐Fe(CO)_4_ plumbylone **9** is observed. Plumbylone **9** can be synthesised by adding Fe(CO)_5_ to compound **6** in a toluene/THF 1:1 mixture at room temperature. While the synthesis with Fe(CO)_5_ afforded **9** in low yield (30%), the use of Fe_2_(CO)_9_ significantly increased the yield to 88%.

For compound **9**, a signal was detected at 2084.01 ppm in the ^207^Pb NMR spectrum, which is high‐field shifted compared to **6** (6542.7 ppm) but in a comparable range of plumbylones **D^Pb^** and **H** (*δ* =  1637.6^[^ ^10^ ^]^ and 2238.9 ppm,^[^ ^15^ ^]^ respectively).

The IR CO stretching vibrations for **7** (*ṽ_CO_*  =  2012, 1981, 1895, 1876 cm^−1^), **8** (*ṽ_CO_*  =  2012, 1983, 1880 cm^−1^), and **9** (*ṽ_CO_*  =  2018, 1985, 1903, 1886 cm^−1^) are blue‐shifted, compared to **4**, **5**, and **6**, respectively. A weaker π‐back donation from the iron center to CO results in a weaker Fe‐CO and a stronger CO bond. This might be caused by a weaker donation from the tetrel center to both iron centers, as two Lewis acids are present in complexes **7** – **9**.

The average HOMA values of the cyclopropenyl moieties for **7**, **8**, and **9** are 0.99, 0.97, and 0.96, respectively. The EDDB values confirm the aromaticity of the cyclopropene rings, with 1.308 for **7**, 1.183 for **8**, and 1.211 for **9**.

To gain deeper insights into the isolated compounds' electronic structure and elucidate the oxidation state of iron, we employed zero‐field ^57^Fe Mössbauer spectroscopy. The Mössbauer isomer shift (δ) indicates the total *s*‐orbital electron density at the iron nucleus, which is influenced by the oxidation state, coordination geometry, spin state, metal‐ligand distances, and the degree of covalency.^[^ ^34^ ^]^ Furthermore, a lower formal oxidation state represents a higher and less negative isomer shift. However, in the case of iron carbonyl species, the opposite trend can be found, such as in Fe(CO)_5_ and Fe(CO)_4_^2−^.^[^ ^35^ ^]^ This can be explained due to π‐back‐bonding, where more electron density is moved towards the iron center. Hence, despite Fe being in a lower formal oxidation state, the Mössbauer values indicate a higher oxidation state.^[^ ^35^ ^]^ Therefore, a comparison of the obtained data of compounds **4** (δ  =  ‐0.13 mm s^−1^; |ΔE_Q_|  =  1.27 mm s^−1^), **5** (δ  =  ‐0.13 mm s^−1^; |ΔE_Q_|  =  1.28 mm s^−1^), **6** (δ  =  ‐0.11 mm s^−1^; |ΔE_Q_|  = 1.36 mm s^−1^), **7** (δ = ‐0.11 mm s^−1^; |ΔE_Q_|  =  1.90 mm s^−1^), and **8** (δ = ‐0.09 mm s^−1^; |ΔE_Q_| = 1.99 mm s^−1^), **9** (δ  =  ‐0.06 mm s^−1^; |ΔE_Q_|  = 2.47 mm s^−1^), to literature‐known iron compounds in oxidation state ‐II (K_2_Fe(CO)_4_ and L^2^ClSi^+^‐Au^+^‐Fe^2−^ L^2^ = bis‐NHI^[^ ^17b^ ^]^) and 0 (Fe(CO)_5_), is crucial (Table 1).

**Table 1:** Comparison of the isomer shift, the quadrupole splitting, and the average Fe‐C and C‐O bond lengths of compound **6** and Fe(CO)_5_ and Fe(CO)_4_^2−^ species.

	4	Fe(CO)_5_	Fe(CO)_4_^2−^	Si‐Au‐Fe^[^ ^17b^ ^]^
Δ [mm s^−1^]	−0.13	−0.08^[^ ^35^ ^]^	−0.19^[^ ^17b^ ^]^	−0.14^[^ ^17b^ ^]^
ΔE_Q_ [mm s^−1^]	1.27	2.55^[^ ^35^ ^]^	0.19^[^ ^17b^ ^]^	0.84^[^ ^17b^ ^]^
avg. Fe‐C [Å]	1.767	1.815^[^ ^36^ ^]^	1.746^[^ ^37^ ^]^	1.760^[^ ^17b^ ^]^
avg C‐O [Å]	1.159	1.142^[^ ^36^ ^]^	1.175^[^ ^37^ ^]^	1.167^[^ ^17b^ ^]^

**Figure 5:**
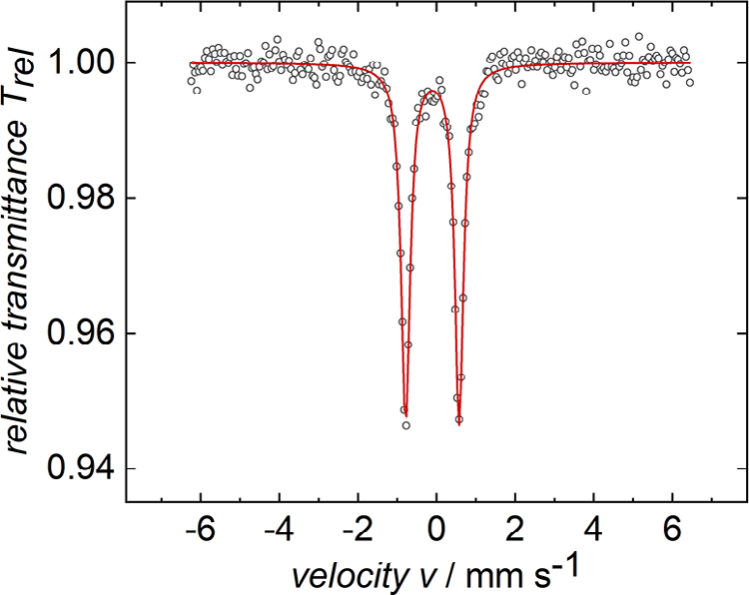
Zero‐field ^57^Fe‐Mößbauer spectrum of compound **4**, recorded in the solid‐state at 77 K. The red trace represents the best fit obtained with the parameters given in the text. Collected data are represented by black circles. δ  =  −0.13 mm s^−1^, |ΔE_Q_|  =  1.27 mm s^−1^, Γ_FWHM_  =  0.28 mm s^−1^.

**Scheme 5:**
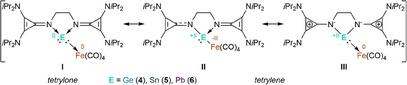
Possible resonance structures of tetrylone (**I**) and tetrylene (**II** and **III**) with covalent or dative E‐Fe(CO)_4_ interactions.

The obtained isomer shift (δ), quadrupole splitting (ΔE_Q_), and the average Fe‐C and C‐O bond lengths of the new compounds **4** – **9** (Figure 5, and SI Figure 44–48) lie intermediate between Fe(CO)_5_, formally assigned a zero oxidation state, and the heterobimetallic Si‐Au‐Fe complex (formal oxidation state ‐II). Thus, this prevents definitive structural assignment via zero‐field ^57^Fe Mössbauer spectroscopy. Possible resonance structures include a tetrylone structure with a dative E→Fe(CO)_4_ interaction (**I**, Scheme 5) or a tetrylene with either covalent (**II**) or dative (**III**) interactions on E‐Fe(CO)_4_ (E = Ge, Sn, Pb), depending on the degree of aromatization of the cyclopropenyl rings and the N‐E interaction.

### 2.4 Computational Studies

In the optimized structures, compared to the free ligand **L^CPI^**, the C═N bonds in the E(II) precursors **1**, **2**, and **3** are elongated, ranging from 1.289 Å (for **L^CPI^**) to 1.320∼1.343 Å. After reduction with Collman's reagent, the C═N bond lengths in the zero‐valent tetrylone‐tetracarbonyl iron complexes (**4**, **5**, **6**) decrease slightly, and the N‐E (E = Ge, Sn, Pb) bond lengths increase. Similarly, compared to precursor **1** – **3**, the Wiberg bond indices (WBIs)^[^ ^38^ ^]^ of N‐E (E = Ge, Sn, Pb) in the corresponding zero‐valent species (**4** – **9**) steadily decrease (Figure S54). The Mayer bond indices (MBO) show the same trend. However, the N‐E values of **4** – **6** (ranging from 0.417 to 0.606) are even smaller than for the bis‐Fe(CO)_4_ complexes (**7**, **8,** and **9** ranging from 0.522 – 0.695) (Figure S59). Calculations suggest that the relative electronic energies of complexes **1** to **9** in the lowest singlet states are thermodynamically more stable than those in the lowest triplet states (ΔEST, Table S5), excluding a diradical ground state.

As shown in Scheme S1, the germanium (**1‐0**)/tin (**2‐0**)/lead (**3‐0**) atom is strongly bonded to the chelating ligand **L^CPI^** with a calculated bond dissociation energy of *D*e  =  56.6, 49.0, and 40.5 kcal mol^−1^ (Δ*G* ^298^ = 46.2, 38.6, and 31.1 kcal mol^−1^), respectively. The Fe(CO)_4_ fragment in the tetracarbonyl iron tetrylone complexes has a calculated bond dissociation energy (BDE) at room temperature of *D*e  =  113.8, 107.0, and 107.6 kcal mol^−1^ for **4**, **5**, and **6**, respectively. The second Fe(CO)_4_ moiety is calculated with a bond strength of 77.9, 74.0, and 59.2 kcal mol^−1^ in complexes **7**, **8**, and **9**, respectively (Scheme S1). Hence, the two Fe(CO)_4_ units are strongly coordinated to the central element. In comparison, the BDE for dissociation of the tetrel element in the parent “free” tetrylones from the chelating ligand **L^CPI^** is *De*  =  56.6, 49.0, and 40.5 kcal mol^−1^ (ΔG^298^  =  46.2, 38.6, and 31.1 kcal mol^−1^) for Ge, Sn, and Pb, respectively. Furthermore, the electron density of delocalized bonds (EDDB) values are lower than those of the isolated compounds **1** – **9**, which underlines the significance of thermodynamic stabilization due to aromatization. The EDDB analysis assessed the aromaticity of the three‐membered rings (3MRs) in the compounds studied (Table S8 and Figure S62).^[^ ^31^ ^]^ Calculations reveal that the EDDB values for the 3MRs in the isolated ligand L^CPI^ (0.844 and 0.829 e) are marginally lower than those in the corresponding model compounds formed by its coordination with Ge (**1‐0**), Sn (**2‐0**), and Pb (**3‐0**), which exhibit EDDB values ranging from 0.848 to 0.957 e. In contrast, the EDDB values for the three‐membered rings in the isolated compounds (**1** – **9**) range from 1.066 to 1.329 e, indicating enhanced aromaticity in these cyclopropenyl rings. This increased aromaticity contributes significantly to the stabilization of the positive charge. These findings underscore the crucial role of aromatic stabilization in facilitating successful isolation and conferring thermodynamic stability to these compounds.

**Figure 6:**
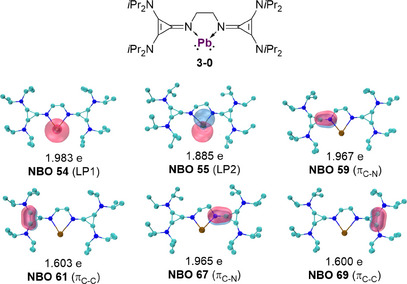
The key orbitals in natural bond orbital analysis (NBO) of plumbylone **3‐0**. The number of occupied electrons is listed below the plot. The isosurface 0.050 a.u. is plotted.

To investigate the electronic structure of the anticipated zero‐valent tetrylone complexes and estimate the stabilization provided by **L^CPI^**, we conducted density functional theory (DFT) calculations at the PBE0‐D3BJ level of theory, using the def2‐SVP basis set for C, O, N, H, Cl, and Br atoms, and the ma‐TZVP basis for Ge, Sn, and Pb atoms.^[^ ^39^ ^]^ Natural bonding orbital (NBO) analyses were performed on the tetrylone model compounds‐ (germylone **1‐0**, stannylone **2‐0** and plumbylone **3‐0**).^[^ ^40^ ^]^ The NBO results revealed two distinctive lone pairs (LP) at the tetrel center. For plumbylone **3‐0**, the s‐type lone pair (LP1) has an electron occupancy of 1.983 e, while the p‐type lone pair (LP2) has a slightly reduced occupancy of 1.885 e (Figure 6). Additionally, the analysis identified four two‐center two‐electron (2c‐2e) π‐bonding orbitals, comprising C‐N π‐bonds and two C‐C π‐bonds. The other model tetrylone compounds show similar NBO characteristics (see SI Figure S65 and S66). These findings align with the dominant resonance structure (NRT), which showed the highest weight contribution (Figure S63)

## 3 Conclusions

In conclusion, we isolated divalent germanium, tin, and lead precursors **1**, **2**, and **3** using a nitrogen‐based bidentate ligand scaffold (**L^CPI^**). We reduced these complexes with Na_2_Fe(CO)_4_ • dioxane and isolated a series of monoatomic zero‐valent tetrylone‐tetracarbonyl iron complexes **4**, **5**, and **6**. Furthermore, we could prove the σ‐donor ability of compounds **4**, **5**, and **6** by reacting them with Fe(CO)_5_ to give the bis‐iron carbonyl compounds **7**, **8**, and **9**.The Complexes **4** – **9** mark the first examples of formal zero‐valent tetrylones stabilized by a bidentate nitrogen‐based donor ligand scaffold (**L^CPI^**). We could determine the 2π‐ aromaticity of the cyclopropene rings of all isolated complexes (**1** – **9**), increasing the compounds' thermodynamic stability. With zero‐field ^57^Fe Mössbauer spectroscopy, we determined the oxidation state of iron (Fe^0^(CO)_4_), which is intermediate between 0 and ‐II. This indicates a complex bonding situation with possible resonance between tetrylone (E^0^) and tetrylene (E^+II^), similar to nitrogen donor‐stabilized tetrylones **J** and **K** (Figure 1).^[^ ^16a–c^ ^]^ Density functional theory (DFT) calculations such as WBI and MBO support the dative interaction of the N‐E bonds, which indicates the zero‐valent tetrylone nature of the complexes **4** – **9**. We introduced the bis‐cyclopropenimine motif as a novel nitrogen donor ligand for low‐valent main‐group chemistry.

## Conflict of Interests

The authors declare no conflict of interest.

## Supporting information



Supporting Information

## Data Availability

The data that support the findings of this study are available in the supplementary material of this article.
