# Supplementary material for: Utilization of a Chelating Bis[(dialkylamino)cyclopropenimine] to Isolate a Series of Heavier Zero‐Valent Group 14 Tetracarbonyl Iron Complexes
Source: Chemistry. 2025 Apr 21;31(29):e202501324. doi: 10.1002/chem.202501324 (PMC12099194; doi:10.1002/chem.202501324)
Supplement: Supplementary file 1 — Supporting Information [file CHEM-31-e202501324-s001.docx]

**Supporting Information**

**Utilization of a Chelating Bis[(dialkylamino)cyclopropenimine] to Isolate a Series of Heavier Zero-Valent Group 14 Tetracarbonyl iron Complexes**

**Authors:** Simone V. Hirmer, Shicheng Dong, Sebastian Stigler, Arseni Kostenko, John A. Kelly, Zihan Zhang, Karsten Meyer, Jun Zhu and Shigeyoshi Inoue*

Content

[1 Experimental section 2](#_Toc193201954)

[1.1 General methods and instrumentation 2](#_Toc193201955)

[1.2 Synthesis and Characterization 3](#_Toc193201956)

[2 Mößbauer Data 35](#_Toc193201957)

[2 Crystallographic Data 42](#_Toc193201958)

[2.1 Single Crystal Analysis of selected Bond lengths and angles 47](#_Toc193201959)

[2.2 Crystal Structures 50](#_Toc193201960)

[3 Computational Details 55](#_Toc193201961)

[3 References 89](#_Toc193201962)

# 1 Experimental section

## 1.1 General methods and instrumentation

All reactions and manipulations were carried out under a dry, oxygen-free argon atmosphere using standard Schlenk techniques or in a Labstar glovebox from MBraun under an argon atmosphere with H_2_O and O_2_ levels below 0.5 ppm if not stated otherwise. Glassware was heat-dried under vacuum before use. Solvents were dried using standard methods, e.g., distilling over sodium/benzophenone or CaH_2_ under argon, and stored over a 3 Å molecular sieve. Pentane was withdrawn from an MBraun solvent purification system and stored in a glovebox over a 3 Å molecular sieve. Deuterated solvents such as C_6_D_6_, THF, and CD_3_CN were dried over a 3 Å molecular sieve in the glovebox. All NMR samples were prepared under argon in J. Young NMR tubes. NMR spectra were recorded on a Bruker Avance 300 MHz, 400 MHz, or 500 MHz spectrometer and referenced against the residual proton resonances of the respective deuterated solvent as internal standard (^1^H and ^13^C). ^109^Sn-NMR and ^207^Pb-NMR spectra are referenced to Me_4_Sn and Pb(NO_3_)_2_ , respectively. The values for the chemical shift (δ) are given in parts per million (ppm). The following abbreviations describe the signal multiplicities: s = singlet, d = doublet, dd = doublet of doublets, t = triplet, sept = septet, bs = broad singlet, m = multiplet. n.o. = not observed. The carbon atoms in the ^13^C spectra were assigned via cross-peaks in 2D correlation experiments (HMBC; HSQC). Infrared (IR) spectra were recorded on a Perkin Elmer FT-IR spectrometer (diamond ATR, Spectrum Two) in a range of 400–4000 cm^-1^ at room temperature inside an argon-filled glovebox. Zero-field ^57^FeMößbauer spectra were recorded on a WissEl Mössbauer spectrometer (MRG-500) at 77 K in constant acceleration mode, with ^57^Co/Rh as the γ-radiation source. WinNormos for Igor Pro software was used to quantitatively evaluate the spectral parameters (least-squares fitting to Lorentzian peaks). The minimum experimental line width was determined at 0.21 mm s^–1^ (full width at half maximum, FWHM). The sample temperature was controlled by an MBBC-HE0106 Mössbauer He/N_2_ cryostat with an accuracy of +/– 0.3 K. Least squares fitting of the Lorentzian signals was carried out with the “Mfit” software, developed by Dr. Eckhard Bill (MPI CEC, Mülheim/Ruhr).^[1-2]^ The isomer shifts were reported relative to α-iron reference at 300 K. Melting points (m.p.) were conducted by the microanalytical laboratory of the Catalysis Research Center (Technical University of Munich) in glass capillaries, sealed with paraffin wax, under inert gas by a Büchi M-565 melting point apparatus. Liquid Injection Field Desorption Ionization Mass Spectrometry (LIFDI-MS) was measured directly from an inert atmosphere glovebox with a Thermo Fisher Scientific Exactive Plus Orbitrap equipped with an ion source from Linden CMS.^[3]^ The samples were diluted in toluene, or THF and injected under inert atmosphere, the obtained spectra were resolved by mass-to-charge values. Unless otherwise stated, commercially available chemicals were purchased from suppliers and used without further purification—the starting materials GeCl_2_ dioxane,^[4]^ Collmann’s reagent ((Na_2_Fe(CO)_4_ • 1.5 dioxane)^[5]^, and the ligand L^CPI [6]^ were synthesized according to the literature.

## 1.2 Synthesis and Characterization

### 1.2.1 Precursor Complexes

**Chloro-Germyliumylidene 1:**

Ligand **L^CPI^** (100 mg, 189 µmol, 1 eq) was dissolved in THF (3 mL), and GeCl_2_ dioxane (52.5 mg, 189 µmol, 1 eq) in THF (2 mL) was added to the solution and stirred at r.t. for 18 h. Then, the solution was removed via filtration, and the precipitate was dried under reduced pressure. After washing the residue with pentane (3 × 3 mL), the product was obtained as a white solid in 81% (126 mg, 154 µmol) yield. Crystals suitable for X-ray diffraction analysis were obtained by slow vapor diffusion of pentane in a concentrated solution of **1** in THF.

**^1^H-NMR** (400 MHz, C_6_D_6_) δ [ppm] = 4.44 (s, 4H, C*H*_2_), 3.74 (sept, 8H, *J* = 6.9 Hz, NC*H*), 1.13 (d, 48H, *J* = 6.9 Hz, C*H*_3_).

**^13^C-NMR** (101 MHz, C_6_D_6_) δ [ppm] = 123.39 (s, N=*C*), 117.00 (*C*=*C*), 53.31 (s, N-*C*H_2_), 50.92 (s, N-*C*H), 22.24 (s, *C*H_3_)

**LIFDI-MS**: *m/z* calculated for [C_32_H_60_N_6_GeCl]^+^ 637.3780, observed 637.3780.

**m.p.:** 193.0 °C (decomposition)

Figure S 1: ^1^H-NMR spectrum of **1** in C_6_D_6_.

Figure S 2: ^13^C-NMR spectrum of **1** in C_6_D_6_.


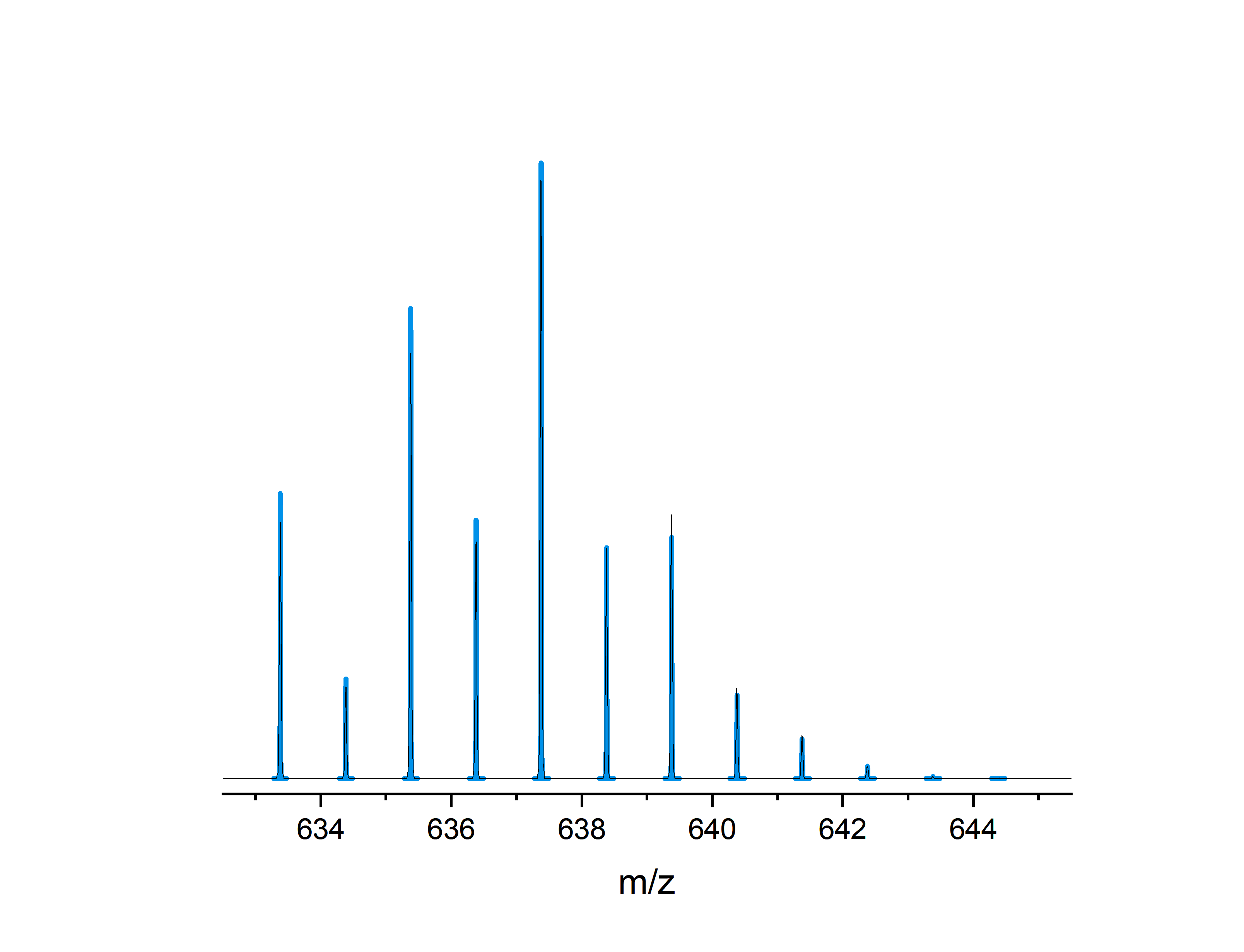


Figure S 3: LIFDI-MS spectra showing the isotopic pattern of compound **1**, measured spectra in grey, simulated in blue.

**Tin (II) precursor 2:**

Ligand **L^CPI^** (300 mg, 567 µmol, 1 eq) was dissolved in THF (15 mL), and SnBr_2_ (158 mg, 567 µmol, 1 eq) was added to the solution and stirred at r.t. for 18 h. Then, the solvent was removed *in vacuo,* and the obtained slightly orange residue was washed with pentane (3 × 5 mL) and subsequently dried. The product was obtained as a white solid with a yield of 95% (437 mg, 541 µmol). Crystals suitable for X-ray diffraction analysis were obtained by in a concentrated solution of **2** in THF.

**^1^H-NMR** (400 MHz, C_6_D_6_) δ [ppm] = 4.29 (s, 4H, C*H*_2_), 3.64 (sept, 8H, *J* = 6.9 Hz, NC*H*), 1.10 (d, 48H, *J* = 6.9 Hz, C*H*_3_).

**^13^C-NMR** (101 MHz, C_6_D_6_) δ [ppm] = 127.60 (s, N=*C*), 117.16 (*C*=*C*), 54.57 (s, N-*C*H_2_), 50.52 (s, N-*C*H), 22.23 (s, *C*H_3_).

**^119^Sn-NMR** (149 MHz, C_6_D_6_) δ [ppm] = -223.76 (s, *Sn*Br_2_)

**LIFDI-MS**: *m/z* calculated for [C_32_H_60_N_6_SnBr]^+^ 727.3085, observed 727.3023.

**m.p.:** 183.2 °C (decomposition)

Figure S 4: ^1^H-NMR spectrum of **2** in C_6_D_6_. (contains traces of THF)

Figure S 5: ^13^C-NMR spectrum of **2** in C_6_D_6_.

Figure S 6: ^119^Sn-NMR spectrum of **2** in C_6_D_6_.


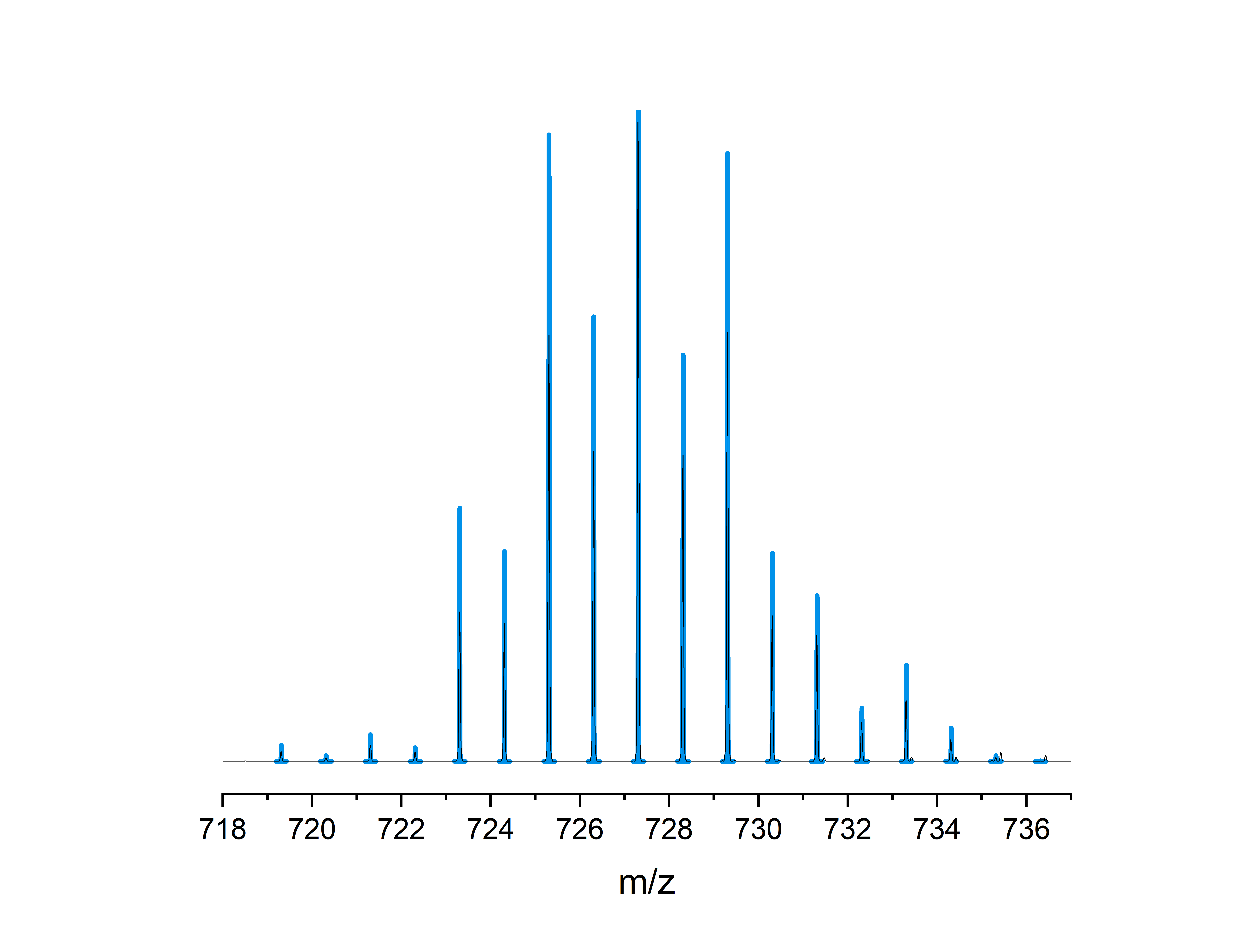


Figure S 7: LIFDI-MS spectra showing the isotopic pattern of compound **2**, measured spectra in grey, simulated in blue.

**Lead (II) precursor 3:**

Ligand **L^CPI^** (100 mg, 189 µmol, 1 eq) was dissolved in THF (5 mL), and PbBr_2_ (69.4 mg, 189 µmol, 1 eq) was added to the solution. The reaction mixture was stirred at r.t. for 24 h. Then, the solvent was removed under reduced pressure, and the residue was washed with pentane (3 ×3 mL). The product was obtained as a white solid in 92% (156 mg, 175 µmol) yield. Crystals suitable for X-ray diffraction analysis were obtained by slow vapor diffusion of pentane in a concentrated solution of **3** in benzene.

**^1^H-NMR** (400 MHz, C_6_D_6_) δ [ppm] = 4.48 (s, 4H, C*H*_2_), 3.56 (sept, 8H, *J* = 6.9 Hz, NC*H*), 1.10 (d, 48H, *J* = 6.8 Hz, C*H*_3_).

**^13^C-NMR** (101 MHz, C_6_D_6_) δ [ppm] = 130.45 (s, N=*C*), 117.53 (*C*=*C*), 58.95 (s, N-*C*H_2_), 50.23 (s, N-*C*H), 22.20 (s, *C*H_3_).

**^207^Pb-NMR** δ [ppm] = 1264.31 ppm.

**EA:** calculated C 42.90, H 6.75, N 9.38; found C 42.67, H 6.88, N 9.33.

**LIFDI-MS**: *m/z* calculated for [C_32_H_60_N_6_Pb_1_Br_1_]^+^ 815.3829, observed 815.3741.

**m.p.:** 195.4 °C (decomposition)

Figure S 8: ^1^H-NMR spectrum of **3** in C_6_D_6_.

Figure S 9: ^13^C-NMR spectrum of **3** in C_6_D_6_.

Figure S 10: ^207^Pb-NMR spectrum of **3** in C_6_D_6_.


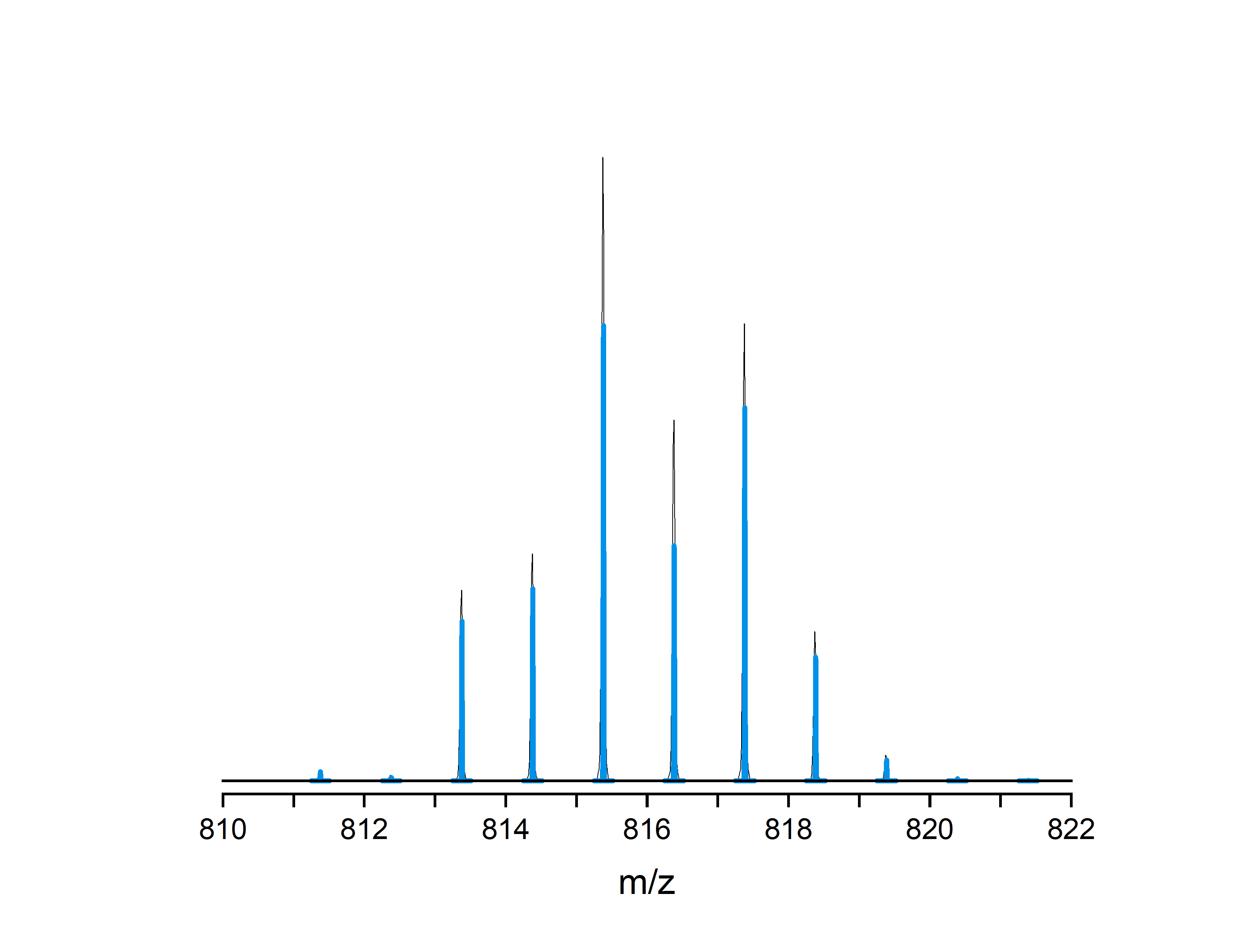


Figure S 11: LIFDI-MS spectra showing the isotopic pattern of compound **3**, measured spectra in grey, simulated in blue.

### 1.2.3 Reduction

**Germylone-Fe(CO)_4_ complex 4:**

In a schlenk flask, compound **1** (150 mg, 223 µmol, 1 eq) was suspended in THF (10 mL) and cooled to -78°C. Then Na_2_Fe(CO)_4_ (77.2 mg, 223 µmol, 1 eq) dissolved in THF (10 mL) was added dropwise. The reaction mixture turned brownish-red and was stirred for 1h in the cold. Then, slowly thawed to room temperature and stirred overnight. Afterwards, the suspension was filtrated, and the filtrate was dried *in vacuo*. The obtained precipitate was extracted with THF (3 × 5 mL), and subsequently, all volatiles were removed under reduced pressure. The crude product was washed with pentane (3 × 2 mL), dried, and yielded complex **4** as a brownish-yellow solid in 73% (124 mg, 161 µmol). Crystals suitable for X-ray diffraction analysis were obtained by slow vapor diffusion of pentane in a concentrated solution of **4** in THF.

**^1^H-NMR** (400 MHz, THF-d_8_) δ [ppm] = 4.07 – 3.94 (m, 10H, C*H*_2_, NC*H*); 3.77 – 3.71 (m, 2H, C*H*_2_), 1.32 (dd*, 48H, *J* = 6.9 Hz, C*H*_3_).

**^13^C-NMR** (101 MHz, THF-d_8_)) δ [ppm] = 224.80 (*C*O), 126.50 (s, N=*C*), n.o. (*C*=*C*), 53.48 (s, N-*C*H_2_), 51.46 (s, N-*C*H), 22.52 (s, *C*H_3_), 22.41 (s, CH_3_).

**IR** (cm^-1^): 2970 (w), 2931 (w), 2871 (w), 1948 (m, CO), 1905 (m, CO), 1827 (s, CO), 1489 (m), 1437 (m), 1365 (w), 1324 (m), 1205 (m), 1116 (w), 1038 (w), 922 (w), 805 (w), 617 (s), 500 (w).

**LIFDI-MS**: *m/z* calculated for [C_36_H_60_N_6_GeFeO_4_]^+^ 771.3310, observed 771.3339 [MH^+^].

**m.p.:** 115.7 °C (decomposition)

**Note: pseudo doublet von doublets resulting from two independent overlapping doublets due to rotational constraints.*

Figure S 12: ^1^H-NMR spectrum of **4** in THF-d_8_ (pentane impurities are marked with *).

Figure S 13: ^13^C-NMR **4** in THF-d_8_ (pentane impurities are marked with *).


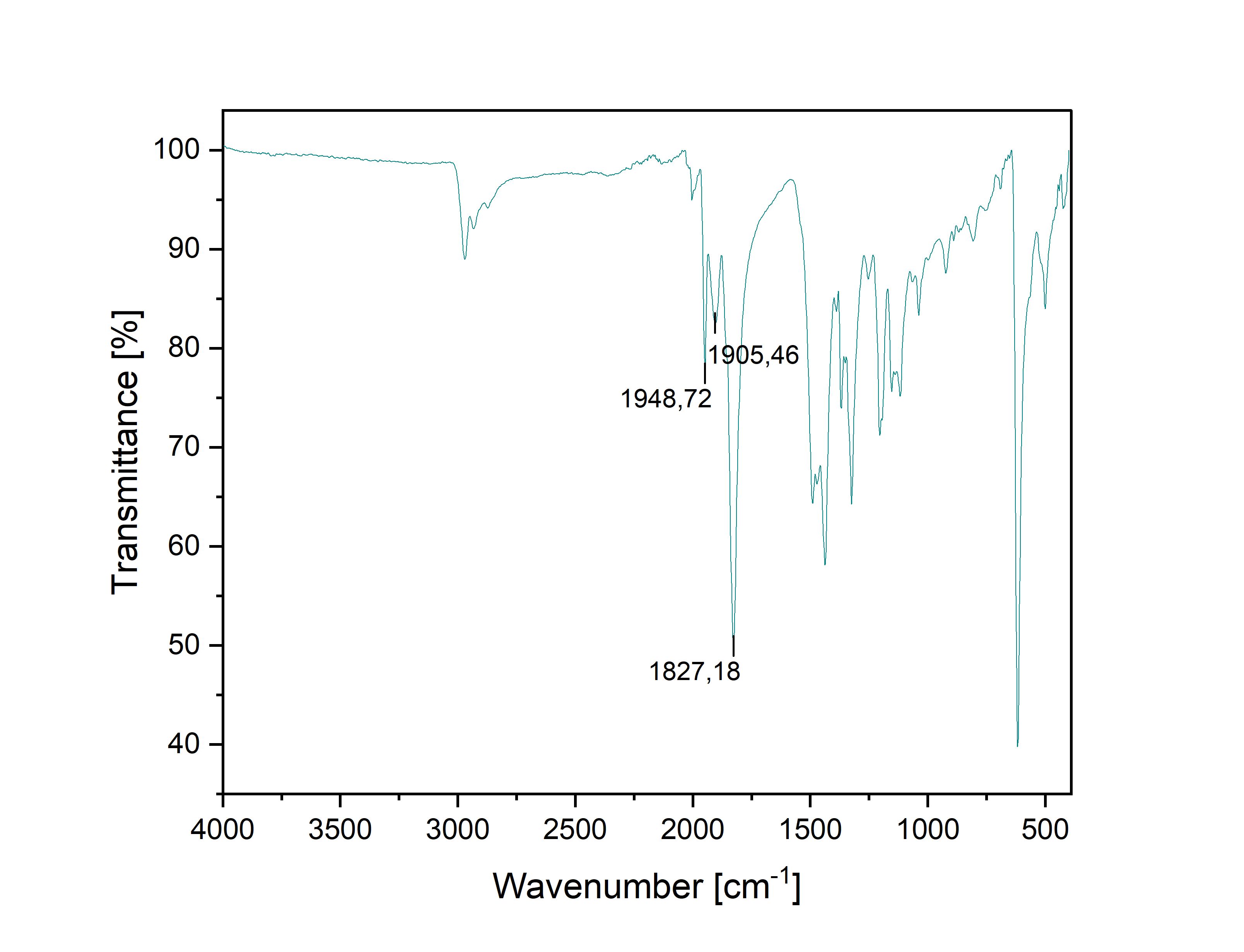


Figure S 14: Solid-state FT-IR spectrum of **4**, the position of the CO bands are marked.


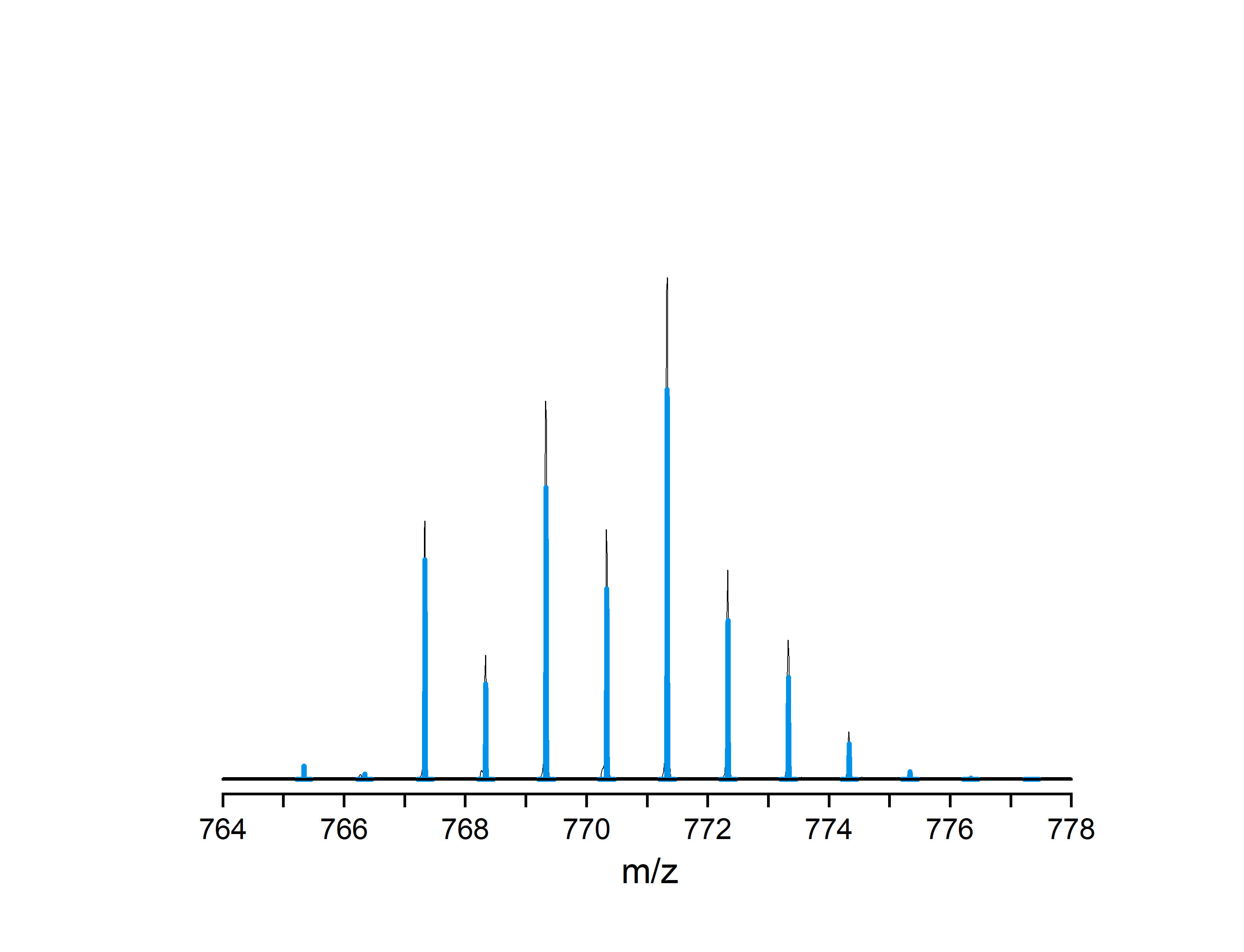


Figure S 15: LIFDI-MS spectra showing the isotopic pattern of compound **4,** measured spectra in grey, simulated in blue.

**Stannylone-Fe(CO)_4_ complex 5:**

The synthesis of **5** was conducted in the same manner as for complex **4**. Compound **2** (250 mg, 310 µmol, 1 eq) is suspended in THF (15 mL) and cooled to -78°C, Na_2_Fe(CO)_4_ (107 mg, 310 µmol, 1 eq) is dissolved in THF (15 mL) and added dropwise. Upon addition, the reaction mixture turns from yellow to dark red. The solution is kept at -78°C for one hour, then thawed to r.t and stirred for 3h. Afterward, the solvent is removed *in vacuo,* and the solid is extracted with toluene (3 × 10 mL). Following, the solvent was concentrated in vacuum and placed in the freezer at -33°C to induce precipitation. After two days, the residual solvent was removed *via* a syringe, and the precipitate was dried and subsequently washed with pentane (3 × 5 mL). Compound **5** was isolated as a yellowish-red solid with a yield of 68% (172 mg, 211 µmol). Crystals suitable for X-ray diffraction analysis were obtained in a concentrated solution of **5** in Et_2_O.

**^1^H-NMR** (400 MHz, C_6_D_6_) δ [ppm] = 4.39 – 4.29 (m, 2H, C*H*_2_), 3.91 – 3.79 (m, 2H, C*H*_2_), 3.58, (bs, 8H, NC*H*), 1.15 – 0.99 (m, 48H, C*H*_3_).

**^13^C-NMR** (101 MHz, C_6_D_6_) δ [ppm] = 224.11 (*C*O), 129.40, (s, N=*C*), n.o. (*C*=*C*), 55.22 (s, N-*C*H_2_), 50.45 (s, N-*C*H), 22.19, 21.88. (s, CH_3_).

**^119^Sn-NMR** (112 MHz, C_6_D_6_) δ [ppm] = 893.79.

**IR** (cm^-1^): 2972 (w), 2931 (w), 2875 (w), 1948 (m, CO), 1906 (m, CO), 1860 (m, CO), 1825 (s, CO), 1487 (m), 1468 (m), 1435 (s), 1367 (w), 1326 (m), 1203 (m), 1151 (w), 1139 (w), 1124 (w), 1036 (w), 914 (w), 617 (s), 498 (w).

**LIFDI-MS**: *m/z* calculated for [C_36_H_60_N_6_SnFeO_4_]^+^ 816.3047, observed 816.3047.

**m.p.:** 163.6 °C

Figure S 16: ^1^H-NMR of compound **5** in C_6_D_6_.

Figure S 17: ^13^C-NMR spectrum of **5** in C_6_D_6_.

Figure S 18: ^119^Sn-NMR of compound **5** in C_6_D_6_.

Figure S 19: ^1^H-^13^C-HSQC spectrum of **5**.





Figure S 20: Solid-state FT-IR spectrum of **5**, the position of the CO bands are marked.





Figure S 21: LIFDI-MS spectra showing the isotopic pattern of compound **5**, measured spectra in grey, simulated in blue.

**Plumbylone-Fe(CO)_4_ complex 6:**

Compound **3** (103 mg, 115 µmol, 1 eq) was dissolved in THF (10 mL) and cooled to -78°C, and Na_2_Fe(CO)_4_ (39.8 mg, 114 µmol, 1 eq) dissolved in THF (7 mL) was added dropwise. Upon addition, the reaction mixture turns from yellow to dark red. The solution is kept at -78°C for one hour, then thawed to r.t and stirred for 30 min. Then, the solvent was removed in vacuo, and the solid was extracted with toluene (2 × 5 mL). Subsequent drying and washing of the crude product with pentane (3 × 2 mL) yielded **6** as a red solid in 55% (57.0 mg, 63.0 µmol) yield. Crystals suitable for X-ray diffraction analysis were obtained by slow vapor diffusion of pentane in a concentrated solution of **6** in benzene.

**^1^H-NMR** (400 MHz, THF-d_8_) δ [ppm] = 3.97 (m, 2H, C*H*_2_), 3.81 (sept, *J* = 6.9 Hz, 8H, NC*H*) 3.67 (m, 2H C*H*_2_), 1.29 (dd*, *J* = 6.8 Hz, 48H, C*H*_3_).

**^13^C-NMR** (101 MHz, THF-d_8_)) δ [ppm] = 223.79 (s, *C*O), 130.84 (s, N=*C*), 116.88 (s, *C*=*C*), 59.76 (s, N-*C*H_2_), 50.81 (s, N-*C*H), 22.58 (s, *C*H_3_), 22.35(s, *C*H_3_).

**^207^Pb-NMR** (63 MHz, THF-*d_8_*) δ [ppm] = 6542.69.

**IR** (cm^-1^): 2968 (w), 2931 (w), 2871 (w), 1951 (m, CO), 1866 (m, CO), 1850 (s, CO), 1823 (s, CO), 1491 (m), 1431 (s), 1367 (w), 1324 (m), 1205 (m), 1151 (m),1139 (m), 1122 (m), 1036 (w), 906 (w), 887 (w), 848 (w), 815 (w), 613 (s), 502 (w).

**LIFDI-MS**: *m/z* calculated for [C_36_H_60_N_6_PbFeO_4_]^+^ 904.3792, observed 904.3727.

**m.p.:** 143.4 °C (decomposition)

**Note: pseudo doublet von doublets resulting from two independent overlapping doublets due to rotational constraints.*

Figure S 22: ^1^H-NMR spectrum of **6** in THF-d_8_ with superimposed region of multiples.

Figure S 23: ^13^C-NMR spectrum of **6** in THF-d_8_.

Figure S 24: ^1^H-^13^C-HMBC spectrum of **6**.

Figure S 25: ^1^H-^13^C-HSQC spectrum of **6**.

Figure S 26: ^207^Pb-NMR of **6** in THF-d_8_.


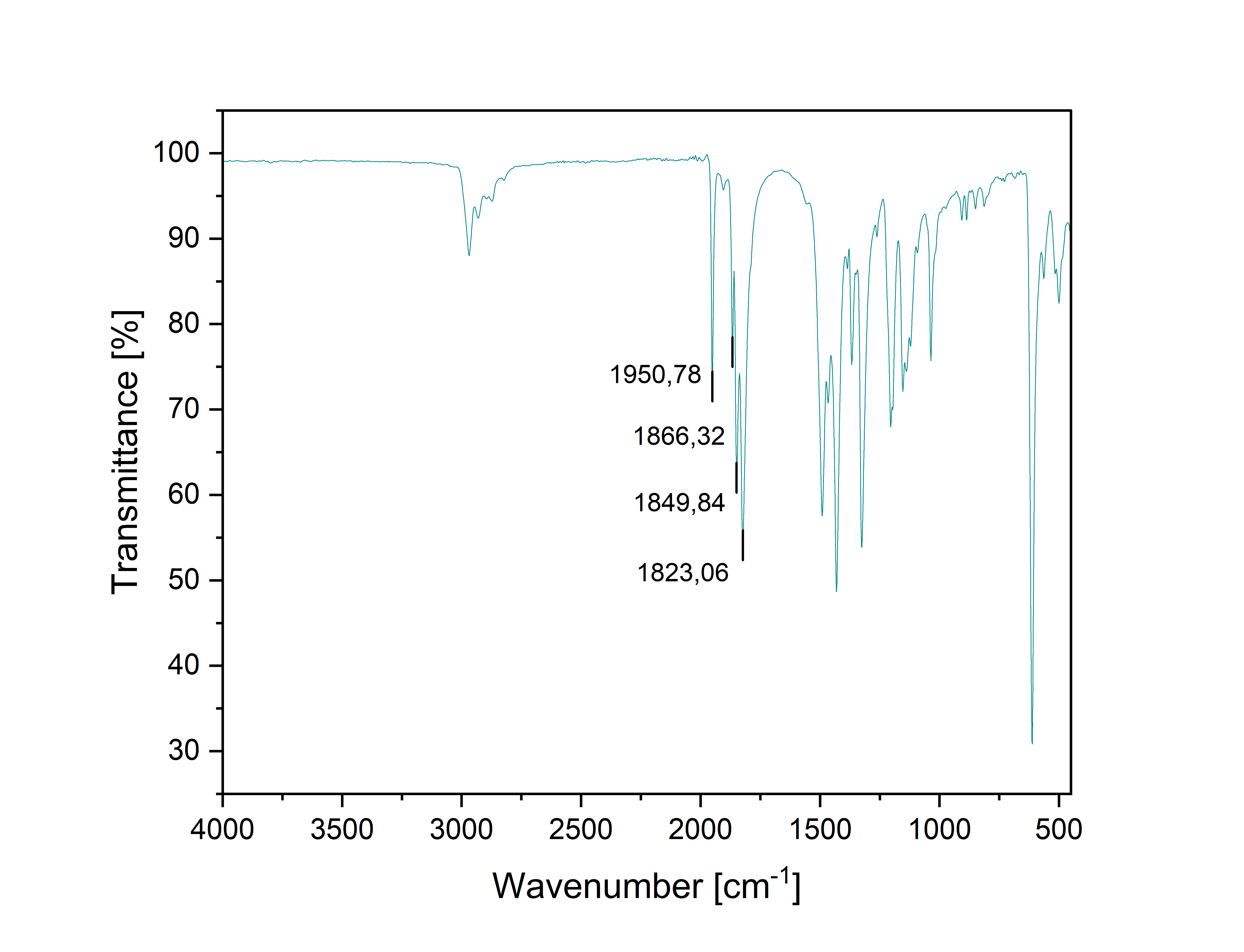


Figure S 27: Solid-state FT-IR spectrum of **6**, the position of the CO bands are marked.


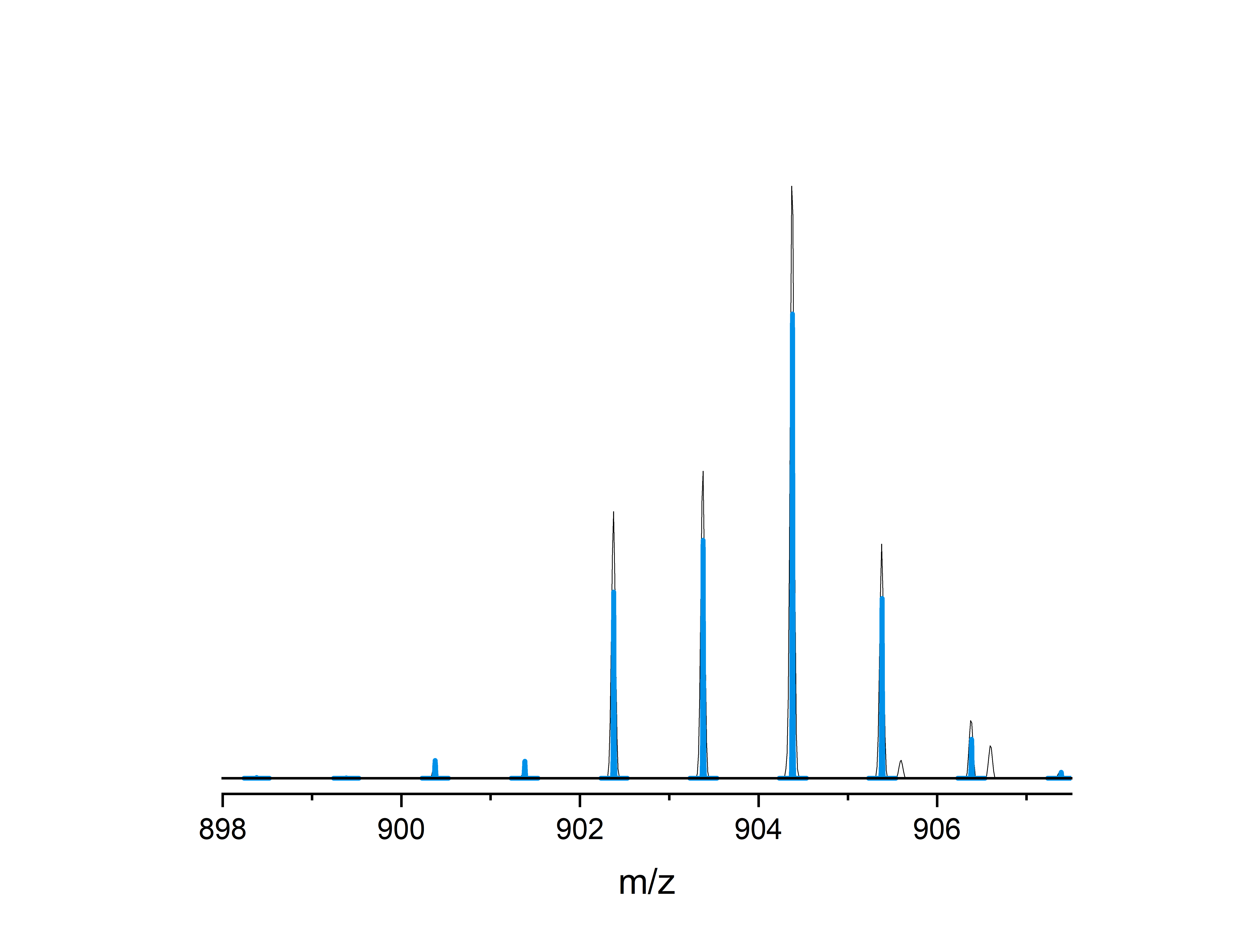


Figure S 28: LIFDI-MS spectra showing the isotopic pattern of compound **6**, measured spectra in grey, simulated in blue.1.2.3 Further Reactivity

**Germylone-[Fe(CO)_4_]_2_ complex 7:**

For the synthesis of compound **7**, complex **4** (75.0 mg. 97.5 µmol, 1 eq.) is suspended in Toluene (7 mL), and Fe(CO)_5_ (19.1 mg, 13.1 mL, 97.5 µmol, 1 eq) is added. Then, the mixture was heated at 80°C for two days. Subsequent filtration of the suspension and evaporation of the filtrate yielded the raw product as an orange solid. After washing with pentane (3 x 5.00 mL), complex **7** was isolated in 70% (63.6 mg, 67.9 µmol). Crystals suitable for X-ray diffraction analysis were obtained by slow vapor diffusion of pentane in a concentrated solution of **7** in benzene.

**^1^H-NMR** (400 MHz, C_6_D_6_) δ [ppm] = 3.95(s, 4H, C*H*_2_), 3.71 (bs, 8H, NC*H*), 1.10 (d, 48H, *J* = 6.7 Hz, C*H*_3_).

**^13^C-NMR** (126 MHz, C_6_D_6_) δ [ppm] = 219.15 (s, *C*O), 133.75 (s, N=*C*), 53.75 (s, N-*C*H_2_), 51.59 (s, N-*C*H), 21.94 (s, *C*H_3_), *(C=C n.o.).*

**IR** (cm^-1^): 2974 (w), 2935 (w), 2871 (w), 2012 (m, CO), 1981 (m, CO), 1895 (s, CO), 1876 (s, CO), 1468 (m), 1439 (m), 1389 (w), 1369 (w), 1347 (w), 1328 (m), 1205 (w), 1192 (w), 1153 (w), 1128 (w), 1114(w), 1040 (w), 1011 (w), 933 (w), 617 (s), 547 (w), 498 (w).

**LIFDI-MS**: *m/z* calculated for [C_40_H_60_N_6_GeFe_2_O_8_]^+^ 938.2383, observed 938.2295.

**m.p.:** 186.5 °C (decomposition)

Figure S 29 ^1^H-NMR spectrum of **7** in C_6_D_6_.

Figure S 30:^13^C NMR of **7** in C_6_D_6_.





Figure S 31: Solid-state FT-IR spectrum of **7**, the position of the CO bands are marked.





Figure S 32: LIFDI-MS spectra showing the isotopic pattern of compound **7**, measured spectra in grey, simulated in blue.

**Stannylone-[Fe(CO)_4_]_2_ complex 8:**

For the synthesis of compound **8**, complex **5** (21.1 mg, 25.9 µmol, 1 eq) was dissolved in THF (0.5 mL) and cooled to -30 C. Then Fe(CO)_5_ (5.07 mg, 25.9 µmol, 1 eq) was added, and stirred for 4h in the cold, then for 18 h at r.t. Subsequent removal of the solvent and washing with pentane (2 × 0.5 mL) yielded **8** as a red solid in 47% (12.0 mg, 25.9 µmol). Crystals suitable for X-ray diffraction analysis were obtained by slow vapor diffusion of pentane in a concentrated solution of **8** in THF.

**^1^H-NMR** (400 MHz, C_6_D_6_) δ [ppm] = 3.89 (s, 4H, C*H*_2_), 3.67 – 3.52 (m, 8H, NC*H*), 1.07 (d, 48H, *J* = 7.0 Hz, C*H*_3_).

**^13^C-NMR** (101 MHz, C_6_D_6_) δ [ppm] = 217.57 (s, *C*O), 132.77 (s, N=*C*), n.o. (s, C=C), 53.91 (s, N-*C*H_2_), 50.97 (s, N-*C*H), 21.44 (s, *C*H_3_).

**^119^Sn-NMR** (149 MHz, C_6_D_6_) δ [ppm] = 595.45 (s,*Sn*).

**IR** (cm^-1^): 2980 (w), 2937 (w), 2877 (w), 2012 (w, CO), 1983 (m, CO), 1880 (s, CO), 1466 (m), 1437 (m), 1369 (w), 1349 (m), 1328(m), 1217 (m), 1205 (m), 1192 (w), 1151 (w), 1126 (w), 1112 (w), 1040 (w), 1011 (w), 924(w), 823 (w), 617 (s), 504 (m) 451 (s).

**LIFDI-MS**: *m/z* calculated for [C_40_H_60_N_6_SnFe_2_O_8_]^+^ 984.2193, observed 984.2249.

**m.p.:** 102.2 °C

Figure S 33: ^1^H-NMR spectrum of **8** in C_6_D_6_.

Figure S 34: ^13^C-NMR spectrum of **8** in C_6_D_6_.

Figure S 35: ^119^Sn-NMR spectrum of **8** in C_6_D_6_.





Figure S 36: Solid-state FT-IR spectrum of **8**, the position of the CO bands are marked.


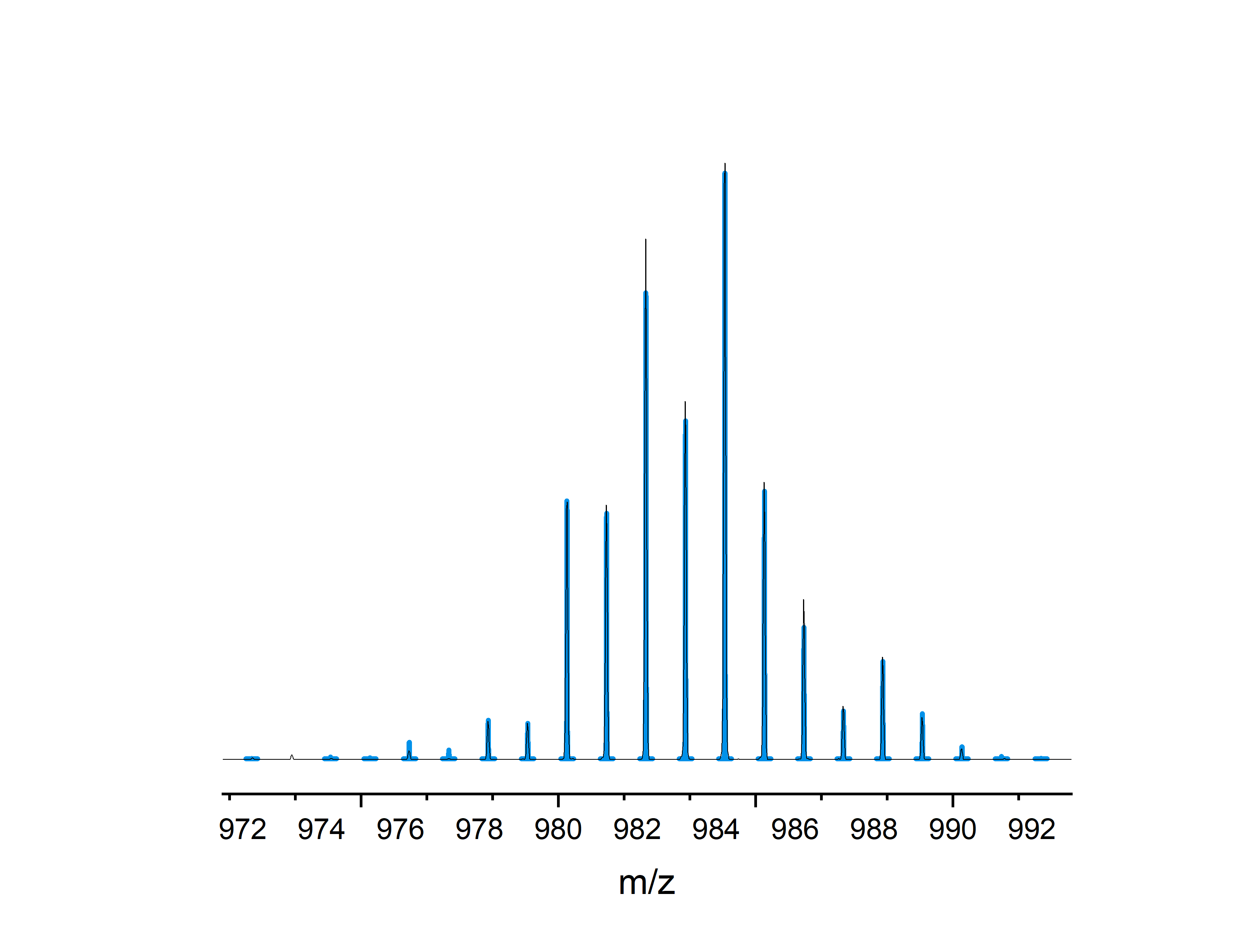


Figure S 37: LIFDI-MS spectra showing the isotopic pattern of compound **8,** measured spectra in grey, simulated in blue.

**Plumbylone-[Fe(CO)_4_]_2_ complex 9:**

Method A: For the synthesis of compound **9**, complex **6** (77.4 mg, 85.6 µmol, 1 eq) was suspended in THF/toluene mixture (1:1 each 1 mL) and Fe_2_(CO)_9_ (31.2 mg, 85.6 µmol, 1 eq) was added as a solid. The solution was stirred at r.t. for 18h, and subsequently the solvent was removed *in vacuo*. The residue was extracted with toluene (3 × 2 mL), and after removing the solvent, compound **9** was isolated as a dark red solid in 88% (81.1 mg, 75.7 µmol) yield.

Method B: Compound **6** (30.0 mg, 33.2 µmol, 1 eq) was dissolved in THF (2 mL) and cooled to -78 C. Then Fe(CO)_5_ (6.50 mg, 33.2 µmol, 1 eq) was added as a diluted solution in THF (1 mL) *via* syringe, and stirred for 4h in the cold and slowly thawed to room temperature. The suspension was filtrated to remove metallic precipitate, and the solvent was removed *in vacuo*. Then, complex **9** was recrystallized from a saturated toluene solution with pentane diffusion as a dark red solid in 31% (11.2 mg, 10.5 µmol).

Crystals suitable for X-ray diffraction analysis were obtained by slow vapor diffusion of pentane in a concentrated solution of **9** in THF, toluene or benzene.

**^1^H-NMR** (400 MHz, C_6_D_6_) δ [ppm] = 3.89 (s, 4H, C*H*_2_), 3.74 – 3.23 (m, 8H, NC*H*), 1.09 (bs, 48H, C*H*_3_).

**^13^C-NMR** (101 MHz, C_6_D_6_) δ [ppm] = 216.26 (*C*O), 133.54 (s, N=*C*), n.o. (*C*=*C*), 57.37 (s, N-*C*H_2_), 50.80 (s, N-*C*H), 21.79 (s, CH_3_).

**^207^Pb-NMR** (63 MHz, C_6_D_6_) δ [ppm] = 2084.01

**IR** (cm^-1^): 2974 (w), 2933 (w), 2873 (w), 2018 (w, CO), 1985 (m, CO), 1903 (s, CO), 1886 (s, CO), 1693 (w), 1561 (w), 1464 (m), 1433 (s), 1365 (m), 1325 (m), 1190 (m), 1151 (m), 1126 (m), 1110 (m), 1029 (w), 912 (w), 815 (w), 753 (w), 611 (s), 496 (m).

**LIFDI-MS**: *m/z* calculated for [C_40_H_60_N_6_PbFe2O_8_]^+^ 1072.2940, observed 1072.2962

**m.p.:** 153.9 °C (decomposition)

Figure S 38: ^1^H-NMR spectrum of **9** in C_6_D_6_.

Figure S 39: ^13^C-NMR spectrum of **9** in C_6_D_6_.

Figure S 40: ^207^Pb-NMR of **9** in C_6_D_6_.

**
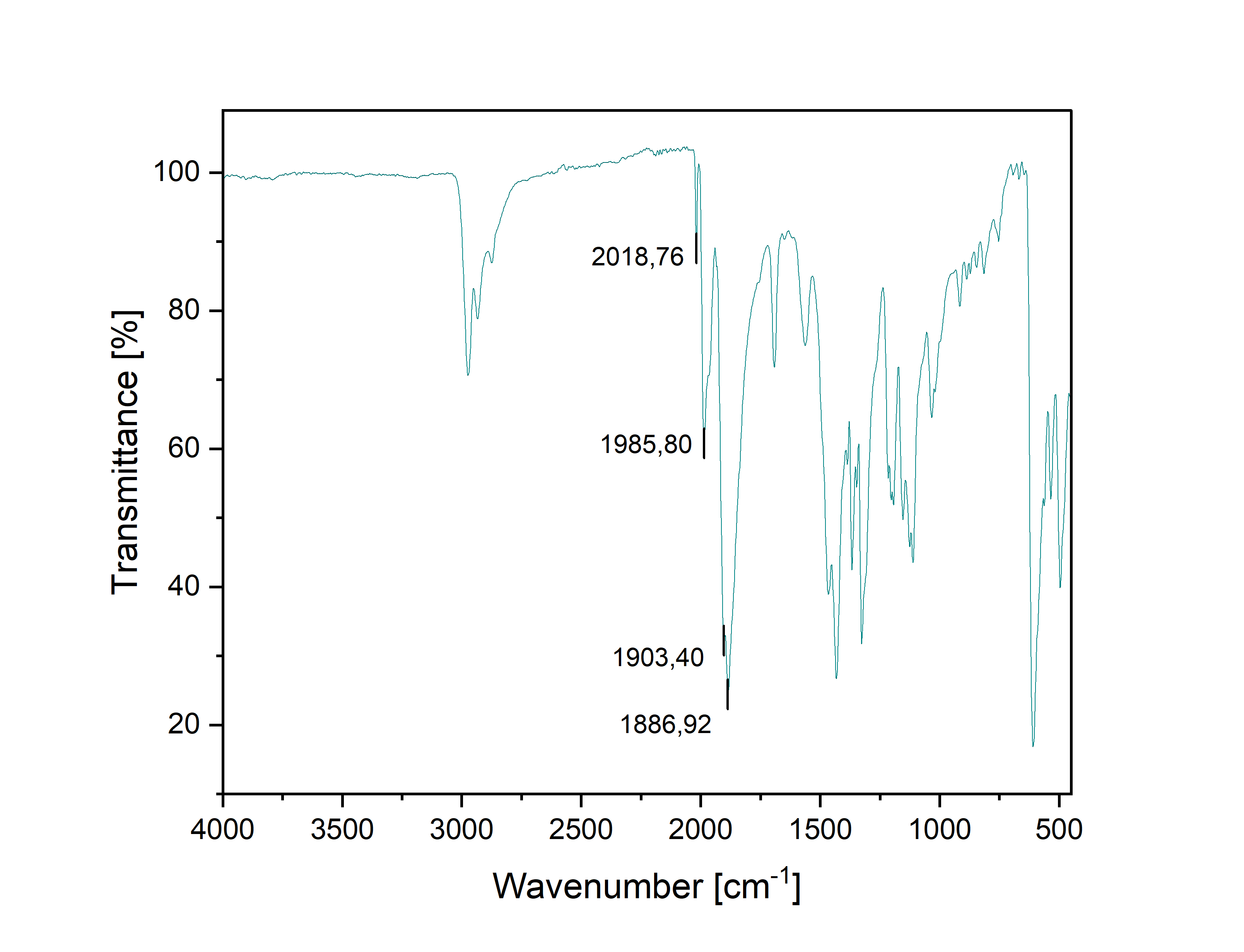
**

Figure S 41: Solid-state FT-IR spectrum of **9**, the position of the CO bands are marked.





Figure S 42: LIFDI-MS spectra showing the isotopic pattern of compound **9,** measured spectra in grey, simulated in blue.

# 2 Mößbauer Data





Figure S 43: Zero-field ^57^Fe-Mößbauer spectrum of compound **4**, recorded in solid state at 77 K. The red trace represents the best fit obtained with the parameters given below. Collected data are represented by black circles.

**^57^Fe Mößbauer data:** δ = −0.13 mm s^−1^, |ΔE_Q_| = 1.27 mms^−1^, Γ_FWHM_ = 0.28 mm s^−1^.





Figure S 44: Zero-field ^57^Fe-Mößbauer spectrum of compound **5**, recorded in solid state at 77 K. The red trace represents the best fit obtained with the parameters given below. Collected data are represented by black circles.

**^57^Fe Mößbauer data:** δ = −0.13 mm s^−1^, |ΔE_Q_| = 1.28 mms^−1^, Γ_FWHM_ = 0.28 mm s^−1^.





Figure S 45: Zero-field ^57^Fe-Mößbauer spectrum of compound **6**, recorded in solid state at 77 K. The red trace represents the best fit obtained with the parameters given below. Collected data are represented by black circles.

**^57^Fe Mößbauer data:** δ = −0.11 mm s^−1^, |ΔE_Q_| = 1.36 mms^−1^, Γ_FWHM_ = 0.27 mm s^−1^.





Figure S 46: Zero-field ^57^Fe-Mößbauer spectrum of compound **7**, recorded in solid state at 77 K. The red trace represents the best fit obtained with the parameters given below. Collected data are represented by black circles.

**^57^Fe Mößbauer data:** δ = −0.11 mm s^−1^, |ΔE_Q_| = 1.90 mms^−1^, Γ_FWHM_ = 0.28 mm s^−1^.





Figure S 47: Zero-field ^57^Fe-Mößbauer spectrum of compound **8**, recorded in solid state at 77 K. The red trace represents the best fit obtained with the parameters given below. Collected data are represented by black circles.

**^57^Fe Mößbauer data:** δ = −0.09 mm s^−1^, |ΔE_Q_| = 1.99 mms^−1^, Γ_FWHM_ = 0.29 mm s^−1^.





Figure S 48: Zero-field ^57^Fe-Mößbauer spectrum of compound **9**, recorded in solid state at 77 K. The red trace represents the best fit obtained with the parameters given below. Collected data are represented by black circles.

**^57^Fe Mößbauer data:** δ = −0.06 mm s^−1^, |ΔE_Q_| = 2.47 mms^−1^, Γ_FWHM_ = 0.28 mm s^−1^.

**Harmonic Oscillator Model of Aromaticity (HOMA)**

The HOMA values were calculated according to the following formulas for hydrocarbon rings:

$$HOMA=1-\frac{a}{n}\sum_{i=1}^{n} \left( R_{i}-R_{opt} \right)^{2}$$

Where R_i_ and R_opt_ (Å) stand for the *i*-th CC bond lengths in the three-membered ring, n is the number of the CC bonds, and the reference benzene ring and in the ring with R_opt_ = 1.388 Å and α = 257.7 Å^–2^.^[7-8]^

Figure S 49: Calculated HOMA values for each cyclopropenyl moiety of compounds **L^CPI^** and **1** – **9**.

# 2 Crystallographic Data

General:

The X-ray intensity data were collected on an X-ray single crystal diffractometer equipped with a CMOS detector (Bruker Photon-100), a rotating anode (Bruker TXS) with MoKα radiation (λ = 0.71073 Å) and a Helios mirror optic by using the APEX4 software package^[9]^ or an X-ray single crystal diffractometer equipped with a CMOS detector (Bruker Photon-100), an IMS microsource with MoKα radiation (λ = 0.71073 Å) and a Helios mirror optic by using the APEX4 software package.^[9]^ The measurement was performed on single crystals coated with perfluorinated ether. The crystal was fixed on the top of a microsampler, transferred to the diffractometer and measured under a stream of cold nitrogen. A matrix scan was used to determine the initial lattice parameters. Reflections were merged and corrected for Lorenz and polarization effects, scan speed, and background using SAINT.^[10]^ Absorption corrections, including odd and even ordered spherical harmonics were performed using SADABS.^[11]^ Space group assignments were based upon systematic absences, E statistics, and successful refinement of the structures. Structures were solved by direct methods with the aid of successive difference Fourier maps, and were refined against all data using the APEX4^[9]^ in conjunction with SHELXL-2018/3.^[12-13]^ and SHELXLE.^[14]^ Methyl hydrogen atoms were refined as part of rigid rotating groups, with a C–H distance of 0.98 Å and Uiso(H) = 1.5·Ueq(C). Other H atoms were placed in calculated positions and refined using a riding model, with methylene and aromatic C-H distances of 0.99 and 0.95 Å, respectively, and Uiso(H) = 1.2·Ueq(C). The crystal structure of **2** was refined as a 2-component inversion twin. The crystal structure of **6** was refined as a 2-component twin. If not mentioned otherwise, non-hydrogen atoms were refined with anisotropic displacement parameters. Full-matrix least-squares refinements were carried out by minimizing Δw(Fo^2^-Fc^2^)^2^ with SHELXL-2014^[15]^ weighting scheme. Neutral atom scattering factors for all atoms and anomalous dispersion corrections for the non-hydrogen atoms were taken from International Tables for Crystallography.^[16]^ Images of the crystal structures were generated by PLATON and MERCURY.^[17-18]^ The CCDC numbers CCDC- (2432139-2432148) contain the supplementary crystallographic data for the structures **L^CPI^**, **1**, **2**, **3**, **4**, **5**, **6**, **7**, **8**, and **9**. These data can be obtained free of charge from the Cambridge Crystallographic Data Centre via https://www.ccdc.cam.ac.uk/structures/.The CIF files were generated using FinalCif.^[19]^

Table S 1: Crystallographic details

|  | L^CPI^ | 1 | 2 | 3 |
| --- | --- | --- | --- | --- |
| CCDC number | 2432148 | 2432142 | 2432139 | 2432141 |
| Empirical formula | C_32_H_60_N_6_ | C_32_H_60_Cl_2_N_6_Ge | C_32_H_60_Br_2_N_6_Sn∙  OC_4_H_8_ | C_32_H_60_Br_2_N_6_Pb |
| Formula weight | 528.86 | 672.35 | 879.47 | 895.87 |
| Temperature [K] | 100(2) | 100(2) | 100(2) | 100(2) |
| Crystal system | monoclinic | monoclinic | monoclinic | monoclinic |
| Space group (number) | $P2_{1}/c$ (14) | $P2_{1}/c$ (14) | $Cc$ (9) | $P2_{1}$ (4) |
| *a* [Å] | 10.6749(6) | 15.821(7) | 12.6437(12) | 11.0795(4) |
| *b* [Å] | 9.9742(5) | 11.763(4) | 18.4951(17) | 11.3370(4) |
| *c* [Å] | 16.0336(10) | 20.397(9) | 19.8674(19) | 16.0708(7) |
| α [°] | 90 | 90 | 90 | 90 |
| β [°] | 99.982(2) | 105.742(14) | 108.533(3) | 105.8560(10) |
| γ [°] | 90 | 90 | 90 | 90 |
| Volume [Å^3^] | 1681.31(16) | 3654(3) | 4405.0(7) | 1941.82(13) |
| *Z* | 2 | 4 | 4 | 2 |
| *ρ*_calc_ [gcm^−3^] | 1.045 | 1.222 | 1.326 | 1.532 |
| *μ* [mm^−1^] | 0.062 | 1.014 | 2.426 | 6.429 |
| *F*(000) | 588 | 1440 | 1816 | 892 |
| Crystal size [mm^3^] | 0.094×0.135×0.173 | 0.205×0.284×0.338 | 0.227×0.258×0.378 | 0.314×0.552×0.598 |
| Crystal color | colorless | colorless | colorless | colorless |
| Crystal shape | plate | plate | fragment | block |
| Radiation | Mo*K_α_* (λ=0.71073 Å) | Mo*K_α_* (λ=0.71073 Å) | Mo*K_α_* (λ=0.71073 Å) | Mo*K_α_* (λ=0.71073 Å) |
| 2θ range [°] | 4.83 to 50.70 (0.83 Å) | 4.04 to 50.70 (0.83 Å) | 4.05 to 50.70 (0.83 Å) | 3.82 to 51.43 (0.82 Å) |
| Index ranges | −12 ≤ h ≤ 12 −12 ≤ k ≤ 12 −19 ≤ l ≤ 19 | −19 ≤ h ≤ 19 −14 ≤ k ≤ 14 −24 ≤ l ≤ 24 | −15 ≤ h ≤ 15 −22 ≤ k ≤ 22 −23 ≤ l ≤ 23 | −13 ≤ h ≤ 13 −13 ≤ k ≤ 13 −19 ≤ l ≤ 19 |
| Reflections collected | 66726 | 134588 | 49785 | 74306 |
| Independent reflections | 3068  *R*_int_ = 0.0434 *R*_sigma_ = 0.0162 | 6673  *R*_int_ = 0.0575 *R*_sigma_ = 0.0520 | 8060  *R*_int_ = 0.0435 *R*_sigma_ = 0.0336 | 7370  *R*_int_ = 0.0522 *R*_sigma_ = 0.0262 |
| Completeness to  θ = 25.242° | 99.6 % | 99.8 % | 100.0 % | 100.0 % |
| Data / Restraints / Parameters | 3068 / 0 / 180 | 6673 / 0 / 404 | 8060 / 42 / 432 | 7370 / 23 / 381 |
| Absorption correction T_min_/T_max_ (method) | 0.7148 / 0.7453  (multi-scan) | 0.6239 / 0.7453  (multi-scan) | 0.5990 / 0.7452  (multi-scan) | 0.3397 / 0.7453  (multi-scan) |
| Goodness-of-fit on *F*^2^ | 1.029 | 0.812 | 0.976 | 1.048 |
| Final *R* indexes  [*I*≥2σ(*I*)] | *R*_1_ = 0.0627 w*R*_2_ = 0.1608 | *R*_1_ = 0.0428 w*R*_2_ = 0.1171 | *R*_1_ = 0.0175 w*R*_2_ = 0.0425 | *R*_1_ = 0.0142 w*R*_2_ = 0.0337 |
| Final *R* indexes  [all data] | *R*_1_ = 0.0682 w*R*_2_ = 0.1661 | *R*_1_ = 0.0599 w*R*_2_ = 0.1256 | *R*_1_ = 0.0185 w*R*_2_ = 0.0429 | *R*_1_ = 0.0144 w*R*_2_ = 0.0337 |
| Largest peak/hole [eÅ^−3^] | 0.75/−0.36 | 0.73/−1.37 | 0.28/−0.31 | 0.64/−0.68 |

|  | 4 | 5 | 6 |
| --- | --- | --- | --- |
| CCDC number | 2432146 | 2432143 | 2432147 |
| Empirical formula | C_36_H_60_FeGeN_6_O_4_∙  OC_4_H_8_ | C_36_H_60_FeN_6_O_4_Sn | C_36_H_60_FeN_6_O_4_Pb∙  C_6_H_6_ |
| Formula weight | 841.44 | 815.44 | 982.04 |
| Temperature [K] | 100(2) | 100(2) | 100(2) |
| Crystal system | triclinic | monoclinic | triclinic |
| Space group (number) | $P\overline{1}$ (2) | $P2_{1}/n$ (14) | $P\overline{1}$ (2) |
| *a* [Å] | 11.8245(11) | 12.6455(9) | 12.055(2) |
| *b* [Å] | 12.5077(12) | 11.9196(7) | 12.7501(17) |
| *c* [Å] | 16.1306(16) | 27.0991(18) | 16.374(2) |
| α [°] | 99.500(3) | 90 | 97.931(5) |
| β [°] | 108.045(3) | 96.344(2) | 109.393(4) |
| γ [°] | 96.204(3) | 90 | 95.916(5) |
| Volume [Å^3^] | 2204.7(4) | 4059.6(5) | 2321.2(6) |
| *Z* | 2 | 4 | 2 |
| *ρ*_calc_ [gcm^−3^] | 1.267 | 1.334 | 1.405 |
| *μ* [mm^−1^] | 1.057 | 1.015 | 3.977 |
| *F*(000) | 896 | 1704 | 1000 |
| Crystal size [mm^3^] | 0.211×0.222×0.236 | 0.167×0.182×0.372 | 0.227×0.282×0.352 |
| Crystal colour | yellow | yellow | orange |
| Crystal shape | block | block | block |
| Radiation | Mo*K_α_* (λ=0.71073 Å) | Mo*K_α_* (λ=0.71073 Å) | Mo*K_α_* (λ=0.71073 Å) |
| 2θ range [°] | 3.84 to 52.84 (0.80 Å) | 4.56 to 51.37 (0.82 Å) | 3.83 to 50.70 (0.83 Å) |
| Index ranges | −14 ≤ h ≤ 14 −15 ≤ k ≤ 15 −20 ≤ l ≤ 20 | −15 ≤ h ≤ 15 −14 ≤ k ≤ 14 −33 ≤ l ≤ 33 | −14 ≤ h ≤ 14 −15 ≤ k ≤ 15 −19 ≤ l ≤ 19 |
| Reflections collected | 65119 | 206307 | 88884 |
| Independent reflections | 8966  *R*_int_ = 0.0462 *R*_sigma_ = 0.0286 | 7694  *R*_int_ = 0.0547 *R*_sigma_ = 0.0173 | 8514  *R*_int_ = 0.0454 *R*_sigma_ = 0.0220 |
| Completeness to  θ = 25.242° | 99.3 % | 99.9 % | 99.9 % |
| Data / Restraints / Parameters | 8966 / 301 / 603 | 7694 / 69 / 479 | 8514 / 282 / 559 |
| Absorption correction T_min_/T_max_ (method) | 0.7014 / 0.7454  (multi-scan) | 0.6813 / 0.7453  (multi-scan) | 0.4719 / 0.7453  (multi-scan) |
| Goodness-of-fit on *F*^2^ | 1.056 | 1.175 | 1.087 |
| Final *R* indexes  [*I*≥2σ(*I*)] | *R*_1_ = 0.0388 w*R*_2_ = 0.1017 | *R*_1_ = 0.0446 w*R*_2_ = 0.1133 | *R*_1_ = 0.0410 w*R*_2_ = 0.1433 |
| Final *R* indexes  [all data] | *R*_1_ = 0.0427 w*R*_2_ = 0.1052 | *R*_1_ = 0.0454 w*R*_2_ = 0.1134 | *R*_1_ = 0.0435 w*R*_2_ = 0.1496 |
| Largest peak/hole [eÅ^−3^] | 0.85/−0.83 | 2.33/−1.09 | 4.29/−2.60 |

|  | 7 | 8 | 9 |
| --- | --- | --- | --- |
| CCDC number | 2432140 | 2432144 | 2432145 |
| Empirical formula | C_40_H_60_Fe_2_GeN_6_O_8_ | C_40_H_60_Fe_2_N_6_O_8_Sn | C_40_H_60_Fe_2_N_6_O_8_Pb |
| Formula weight | 937.23 | 983.33 | 1071.83 |
| Temperature [K] | 100(2) | 100(2) | 100(2) |
| Crystal system | triclinic | triclinic | monoclinic |
| Space group (number) | $P\overline{1}$ (2) | $P\overline{1}$ (2) | $C2/c$ (15) |
| *a* [Å] | 10.084(2) | 9.8913(11) | 28.0584(13) |
| *b* [Å] | 11.262(3) | 21.478(3) | 19.4695(13) |
| *c* [Å] | 22.844(4) | 23.349(3) | 19.9035(14) |
| α [°] | 76.941(10) | 110.451(4) | 90 |
| β [°] | 87.512(7) | 90.636(4) | 123.388(3) |
| γ [°] | 63.537(7) | 102.943(4) | 90 |
| Volume [Å^3^] | 2257.4(9) | 4507.8(9) | 9078.5(10) |
| *Z* | 2 | 4 | 8 |
| *ρ*_calc_ [gcm^−3^] | 1.379 | 1.449 | 1.568 |
| *μ* [mm^−1^] | 1.350 | 1.239 | 4.384 |
| *F*(000) | 980 | 2032 | 4320 |
| Crystal size [mm^3^] | 0.092×0.151×0.232 | 0.289×0.301×0.304 | 0.170×0.197×0.290 |
| Crystal colour | yellow | yellow | red |
| Crystal shape | fragment | block | fragment |
| Radiation | Mo*K_α_* (λ=0.71073 Å) | Mo*K_α_* (λ=0.71073 Å) | Mo*K_α_* (λ=0.71073 Å) |
| 2θ range [°] | 4.13 to 50.05 (0.84 Å) | 3.91 to 50.70 (0.83 Å) | 4.17 to 50.06 (0.84 Å) |
| Index ranges | −12 ≤ h ≤ 12 −13 ≤ k ≤ 13 −27 ≤ l ≤ 27 | −11 ≤ h ≤ 11 −25 ≤ k ≤ 25 −28 ≤ l ≤ 28 | −33 ≤ h ≤ 33 −23 ≤ k ≤ 23 −23 ≤ l ≤ 23 |
| Reflections collected | 87213 | 191317 | 200721 |
| Independent reflections | 7939  *R*_int_ = 0.0476 *R*_sigma_ = 0.0255 | 16099  *R*_int_ = 0.0824 *R*_sigma_ = 0.0430 | 8037  *R*_int_ = 0.0561 *R*_sigma_ = 0.0146 |
| Completeness to  θ = 25.242° | 99.6 % | 97.8 % | 100.0 % |
| Data / Restraints / Parameters | 7939 / 0 / 530 | 16099 / 1242 / 1187 | 8037 / 12 / 530 |
| Absorption correction T_min_/T_max_ (method) | 0.6721 / 0.7452  (multi-scan) | 0.6494 / 0.7453  (multi-scan) | 0.6068 / 0.7453  (multi-scan) |
| Goodness-of-fit on *F*^2^ | 1.162 | 1.259 | 1.232 |
| Final *R* indexes  [*I*≥2σ(*I*)] | *R*_1_ = 0.0316 w*R*_2_ = 0.0752 | *R*_1_ = 0.0881 w*R*_2_ = 0.1901 | *R*_1_ = 0.0473 w*R*_2_ = 0.1002 |
| Final *R* indexes  [all data] | *R*_1_ = 0.0330 w*R*_2_ = 0.0758 | *R*_1_ = 0.1053 w*R*_2_ = 0.1976 | *R*_1_ = 0.0573 w*R*_2_ = 0.1084 |
| Largest peak/hole [eÅ^−3^] | 1.13/−0.38 | 2.22/−1.77 | 7.85/−4.57 |

The data have been assigned the following deposition numbers which can either be quoted as CCDC Numbers or CSD Numbers. A CCDC Number is usually quoted for an organic or metal-organic structure, whereas a CSD Number is usually quoted for an inorganic structure.

CCDC XXXXXXX-YYYYYYY (generally used for organic and metal-organic structures)

CSD XXXXXXX-YYYYYYY (generally used for inorganic structures)

Deposition Number 2432139-2432148

---------------------------------------------------------------
Summary of Data - Deposition Number 2432139
---------------------------------------------------------------
Compound Name: **2**
Data Block Name: data_mo_HirSi10_0m
Unit Cell Parameters: a 12.6437(12) b 18.4951(17) c 19.8674(19) Cc
---------------------------------------------------------------
---------------------------------------------------------------
Summary of Data - Deposition Number 2432140
---------------------------------------------------------------
Compound Name: **7**
Data Block Name: data_HirSi75_0ma
Unit Cell Parameters: a 10.084(2) b 11.262(3) c 22.844(4) P-1
---------------------------------------------------------------
---------------------------------------------------------------
Summary of Data - Deposition Number 2432141
---------------------------------------------------------------
Compound Name: **3**
Data Block Name: data_mo_HirSi56_0ma
Unit Cell Parameters: a 11.0795(4) b 11.3370(4) c 16.0708(7) P21
---------------------------------------------------------------
---------------------------------------------------------------
Summary of Data - Deposition Number 2432142
---------------------------------------------------------------
Compound Name: **1**
Data Block Name: data_HirSi57_0ma
Unit Cell Parameters: a 15.821(7) b 11.763(4) c 20.397(9) P21/c
---------------------------------------------------------------
---------------------------------------------------------------
Summary of Data - Deposition Number 2432143
---------------------------------------------------------------
Compound Name: **5**
Data Block Name: data_hirsi64_0ma
Unit Cell Parameters: a 12.6455(9) b 11.9196(7) c 27.0991(18) P21/n
---------------------------------------------------------------
---------------------------------------------------------------
Summary of Data - Deposition Number 2432144
---------------------------------------------------------------
Compound Name: **8**
Data Block Name: data_HirSi53_0m
Unit Cell Parameters: a 9.8913(11) b 21.478(3) c 23.349(3) P-1
---------------------------------------------------------------
---------------------------------------------------------------
Summary of Data - Deposition Number 2432145
---------------------------------------------------------------
Compound Name: **9**
Data Block Name: data_mo_HirSi76_0ma
Unit Cell Parameters: a 28.0584(13) b 19.4695(13) c 19.9035(14) C2/c
---------------------------------------------------------------
---------------------------------------------------------------
Summary of Data - Deposition Number 2432146
---------------------------------------------------------------
Compound Name: **4**
Data Block Name: data_HirSi58_0m
Unit Cell Parameters: a 11.8245(11) b 12.5077(12) c 16.1306(16) P-1
---------------------------------------------------------------
---------------------------------------------------------------
Summary of Data - Deposition Number 2432147
---------------------------------------------------------------
Compound Name: **6**
Data Block Name: data_mo_HirSi_60_0ma
Unit Cell Parameters: a 12.055(2) b 12.7501(17) c 16.374(2) P-1
---------------------------------------------------------------
---------------------------------------------------------------
Summary of Data - Deposition Number 2432148
---------------------------------------------------------------
Compound Name: **L^CPI^**
Data Block Name: data_HirSi63_0m
Unit Cell Parameters: a 10.6749(6) b 9.9742(5) c 16.0336(10) P21/c
---------------------------------------------------------------

## 2.1 Single Crystal Analysis of selected Bond lengths and angles

**Precursor 1, 2, 3**

Table S 2: Selected bond lengths and angles to compare the E(II) precursors **1**, **2,** and **3**.

| [Å,°] | **1 (Ge)** | **2 (Sn)** | **3 (Pb)** |
| --- | --- | --- | --- |
| N(1;2)-E(1) | 1.955(2); 1.967(2) | 2.181(3); 2.183(3) | 2.340(3); 2.312(3) |
| N(1)-E(1)-N(2) | 82.75(9) | 77.14(9) | 75.69(10) |
| X(1)-E(1)-X(2) | x | 172.136(12) | 165.12(16) |
| C(1)-N(1);N(2)-C(4) | 1.337(3); 1.343(3) | 1.322(4); 1.320(4) | 1.332(5); 1.336(4) |
| C(2)-C(3); C(5)-C(6) | 1.394(4); 1.379(3) | 1.388(5); 1.389(5) | 1.381(5); 1.387(5) |
| C(1)-C(2);C(1)-C(3) | 1.394(3); 1.393(3) | 1.402(4);  1.391(4) | 1.396(5); 1.382(5) |
| C(4)-C(5);C(4)-C(6) | 1.391(3); 1.390(3) | 1.401(4); 1.386(5) | 1.402(5); 1.380(5) |

**Tetracarbonyl iron- tetrylones 4, 5 and 6**

Table S 3: Selected bond lengths and angles for comparison of the tetracarbonyl iron-tetrylones **4**, **5,** and **6**.

| [Å,°] | **4 (Ge)** | **5 (Sn)** | **6 (Pb)** |
| --- | --- | --- | --- |
| N(1;2)-E(1) | 2.0419(18); 2.0282(17) | 2.256(3);  2.248(3) | 2.370(4), 2.393(4) |
| N(1)-E(1)-N(2) | 79.59(7) | 74.94(11) | 73.12(15) |
| E(1)-Fe(1) | 2.5856(4) | 2.6958(6) | 2.7298(8) |
| N(1;2)-E(1)-Fe(1) | 106.78(6);  101.94(5) | 105.71(8);  98.47(8) | 98.97(11), 103.81(12) |
| C(1)-N(1);  N(2)-C(4) | 1.323(3);  1.321(3) | 1.319(5);  1.318(5) | 1.319(7); 1.313(7) |
| C(2)-C(3);  C(5)-C(6) | 1.385(3);  1.382(3) | 1.384(5);  1.378(5) | 1.375(8); 1.376(8) |
| C(1)-C(2);  C(1)-C(3) | 1.400(3);  1.390(3) | 1.401(5);  1.393(5) | 1.395(7); 1.385(8) |
| C(4)-C(5);  C(4)-C(6) | 1.396(3);  1.390(3) | 1.396(5);  1.392(5) | 1.410(8); 1.397(8) |

**Bis-tetracarbonyl iron tetrylones 7, 8 and 9**

Table S 4: Selected bond lengths and angles for comparison of the bis-(tetracarbonyl iron)-tetrylones **7**, **8,** and **9**.

| [Å,°] | **7 (Ge)** | **8 (Sn)** | **9 (Pb)** |
| --- | --- | --- | --- |
| N(1;2)-E(1) | 1.9923(19)  1.9892(19) | 2.174(7);  2.180(7) | 2.370(5); 2.333(6) |
| N(1)-E(1)-N(2) | 81.83(8) | 77.5(3) | 75.5(2) |
| E(1)-Fe(1;2) | 2.3993(6)  2.4084(6) | 2.5339(14);  2.5322(15) | 2.6006(10); 2.5961(10) |
| Fe(1)-E(1)-Fe(2) | 124.86(2) | 128.06(5) | 136.55(3) |
| C(1)-N(1);  N(2)-C(4) | 1.327(3);  1.390(3) | 1.318(11);  1.335(11) | 1.327(9); 1.321(9) |
| C(2)-C(3);  C(5)-C(6) | 1.382(3);  1.387(3) | 1.383(14);  1.383(15) | 1.384(10); 1.385(10) |
| C(1)-C(2);  C(1)-C(3) | 1.390(3);  1.392(3) | 1.388(12);  1.385(13) | 1.397(9);  1.402(9) |
| C(4)-C(5);  C(4)-C(6) | 1.394(3);  1.393(3) | 1.383(13);  1.383(13) | 1.409(10); 1.395(10) |

## 2.2 Crystal Structures


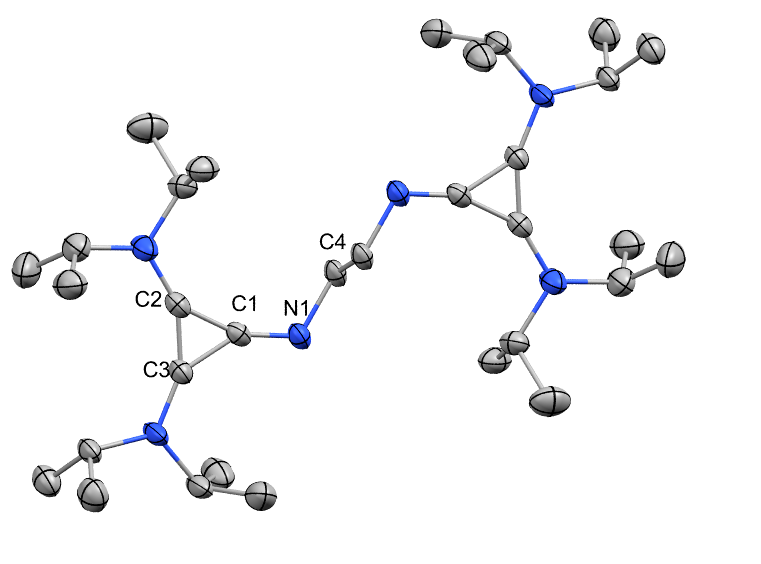


Figure S 50: L thermal ellipsoids' molecular structure is set to a 50% probability level. Hydrogen atoms are omitted for clarity.


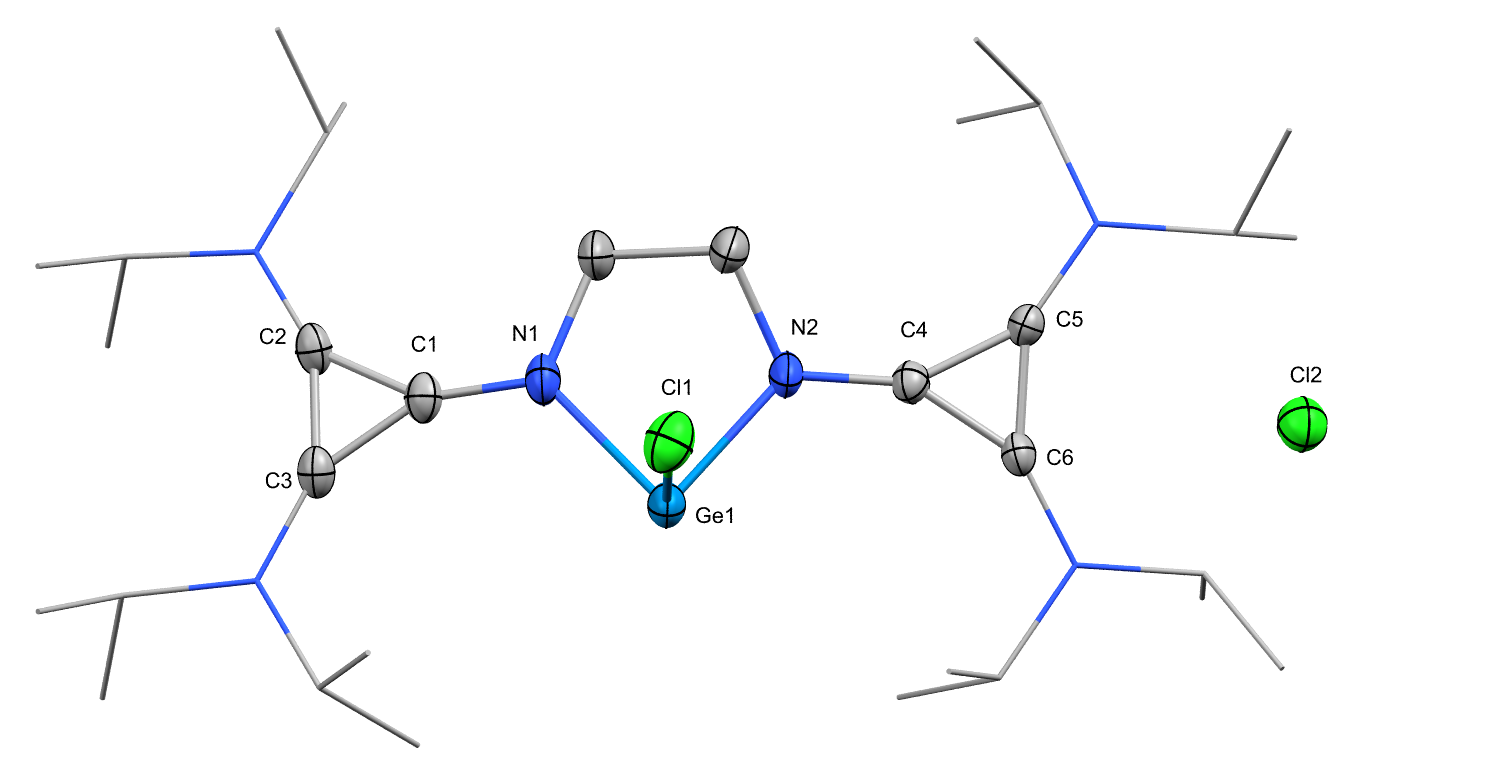


Figure S 51: Molecular structure of **1** thermal ellipsoid is set to 50% probability level. Hydrogen atoms are omitted for clarity; isopropyl‑substituents are depicted as wireframes for simplicity.


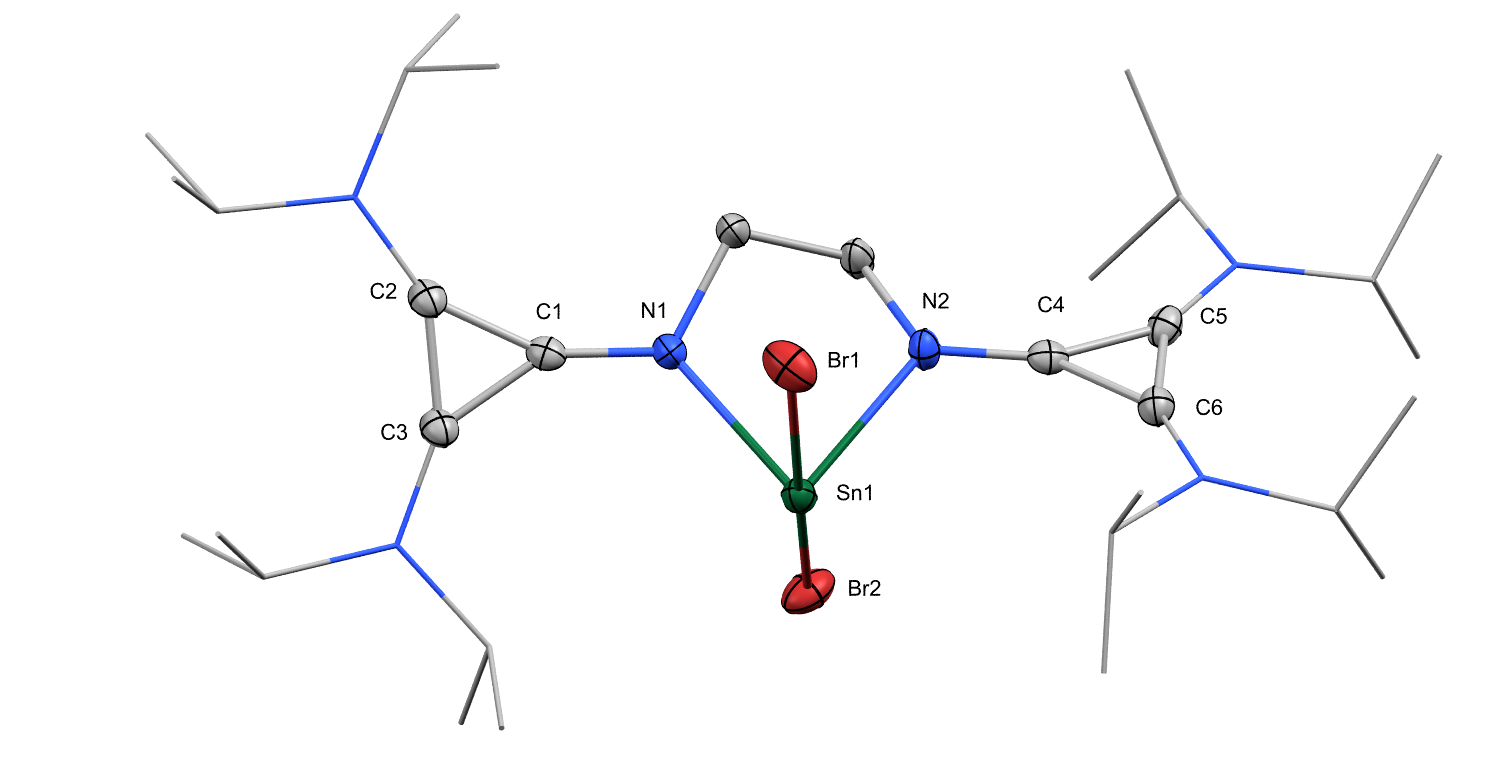


Figure S 52: Molecular structure of **2** thermal ellipsoids is set to 50% probability level. Hydrogen atoms and lattice solvent (THF) are omitted for clarity; isopropyl‑substituents are depicted as wireframes for simplicity.


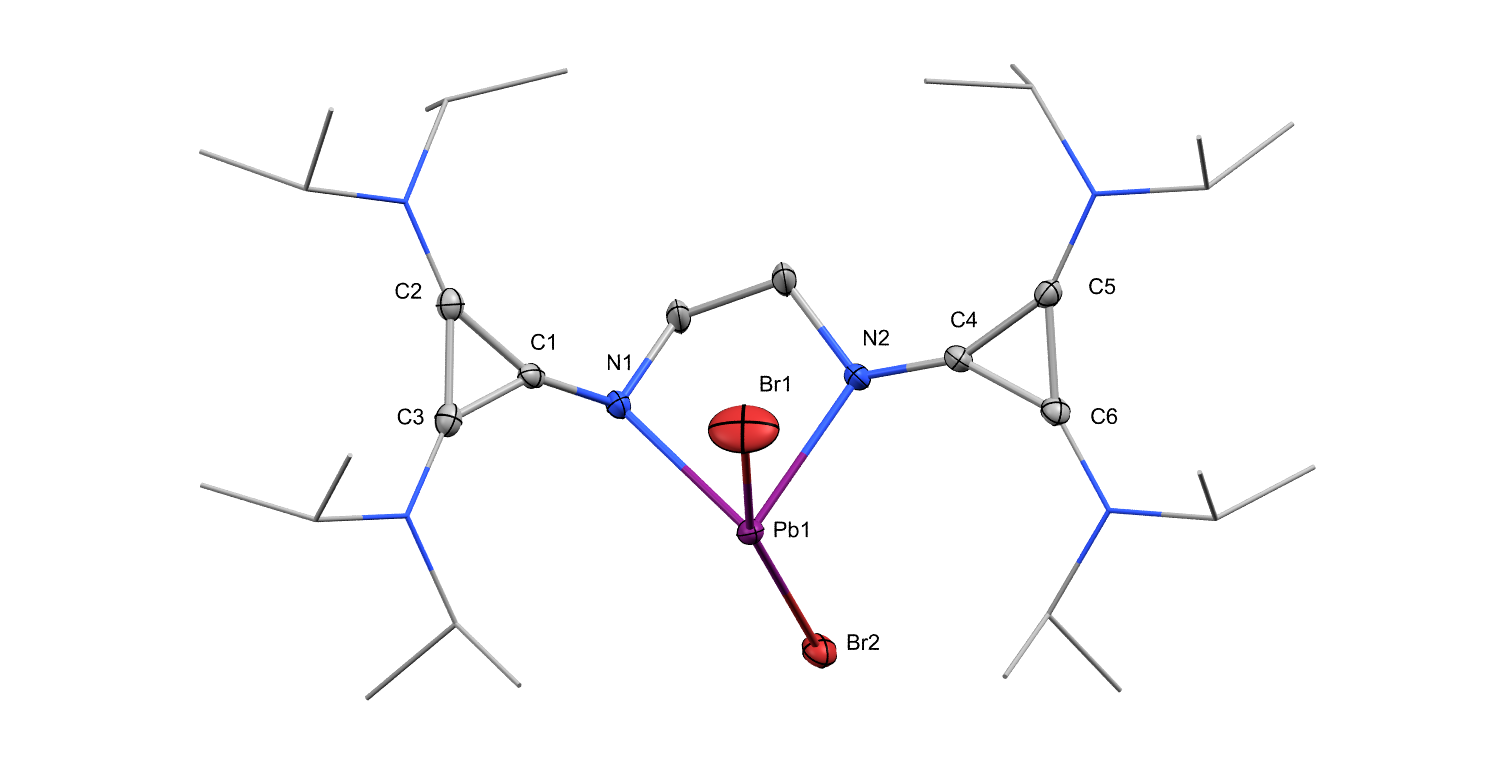


Figure S 53: Molecular structure of **3** thermal ellipsoids is set to 50% probability level. Hydrogen atoms are omitted for clarity; isopropyl‑substituents are depicted as wireframes for simplicity.


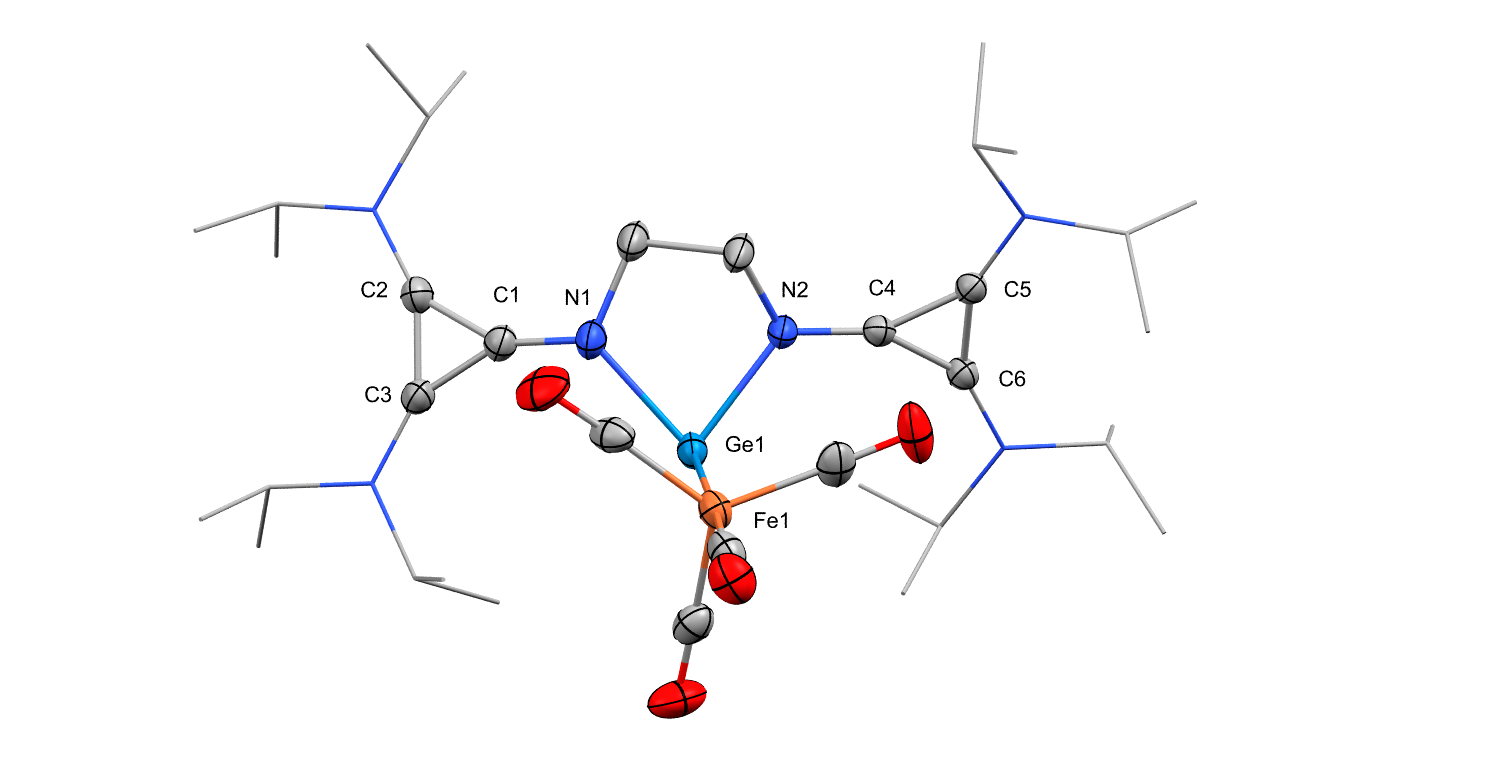


Figure S 54: The molecular structure of 4 thermal ellipsoids is set to a 50% probability level. Hydrogen atoms are omitted for clarity; isopropyl‑substituents are depicted as wireframes for simplicity.


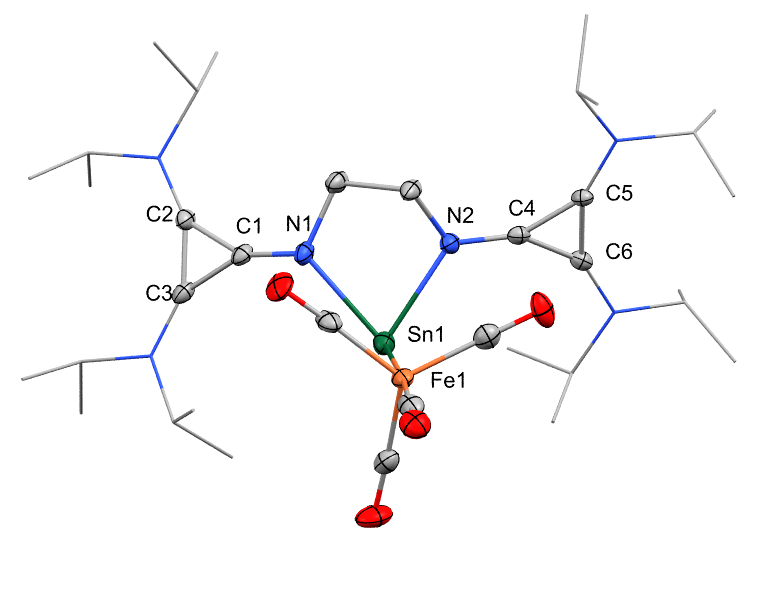


Figure S 55: The molecular structure of **5** thermal ellipsoids is set to a 50% probability level. Hydrogen atoms are omitted for clarity; isopropyl‑substituents are depicted as wireframes for simplicity.


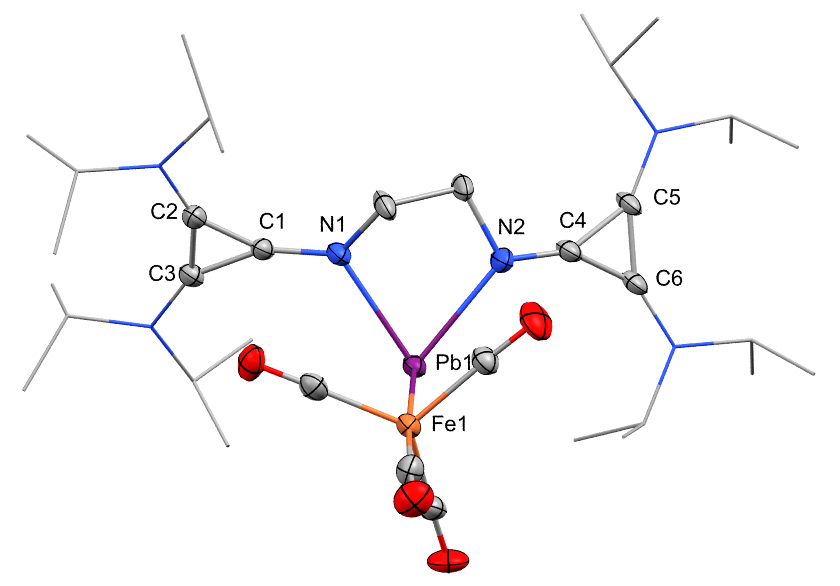


Figure S 56: The molecular structure of **6** thermal ellipsoids is set to a 50% probability level. Hydrogen atoms and lattice solvent (benzene) are omitted for clarity; isopropyl‑substituents are depicted as wireframes for simplicity.


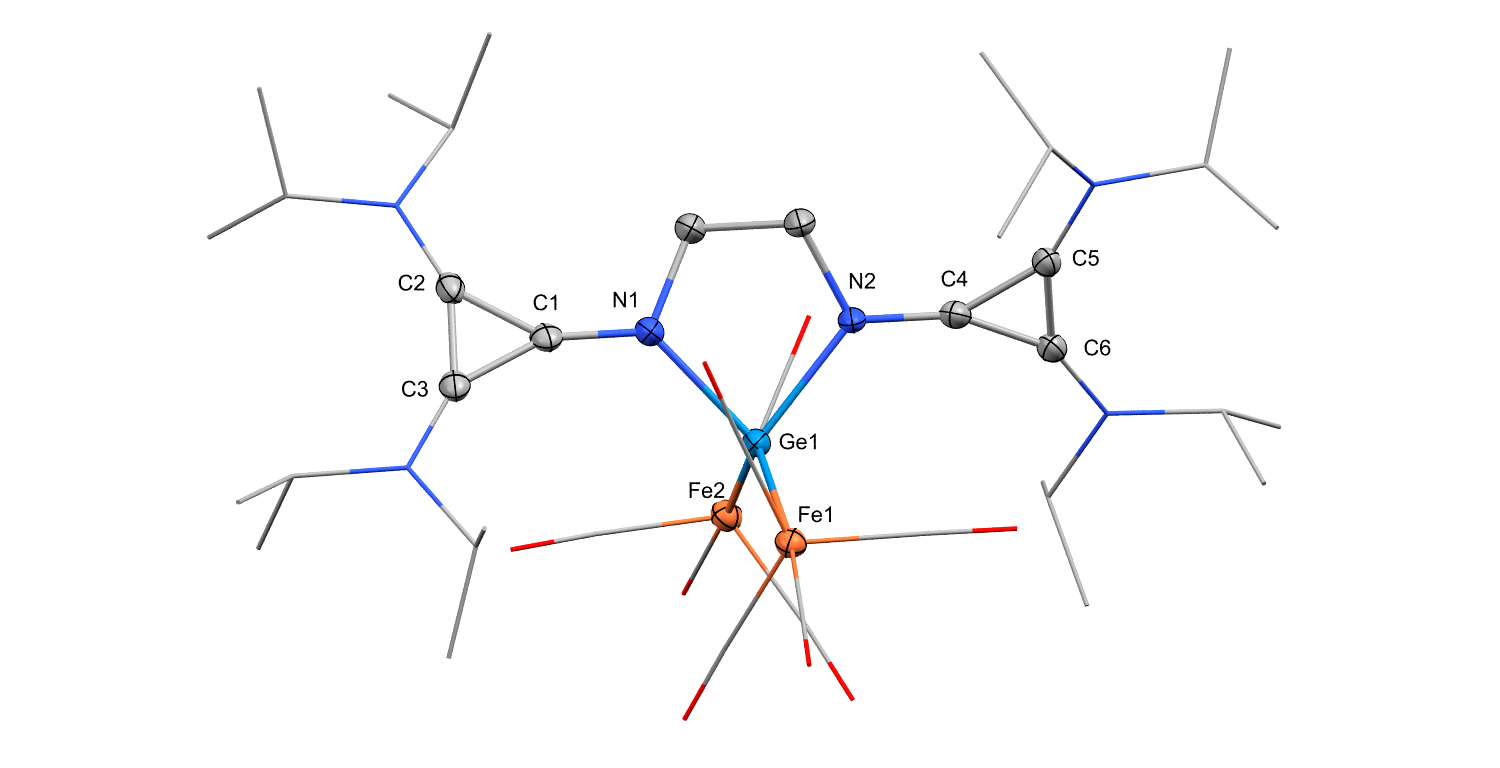


Figure S 57: The molecular structure of **7** thermal ellipsoids is set to a 50% probability level. Hydrogen atoms are omitted for clarity; isopropyl‑substituents and CO are depicted as wireframes for simplicity.


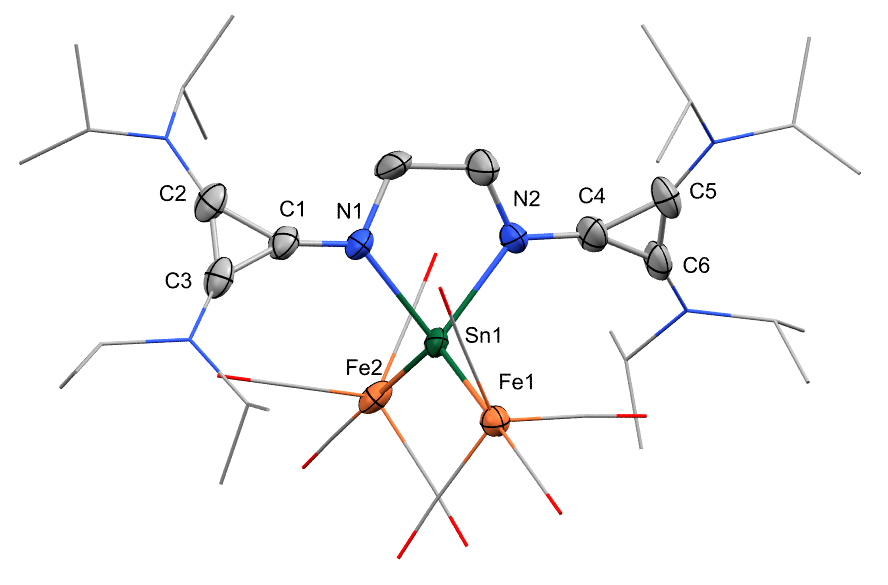


Figure S 58: The molecular structure of **8** thermal ellipsoids is set to a 50% probability level. Hydrogen atoms are omitted for clarity; isopropyl‑substituents and CO are depicted as wireframes for simplicity. The unit cell contains two chemically independent structures.


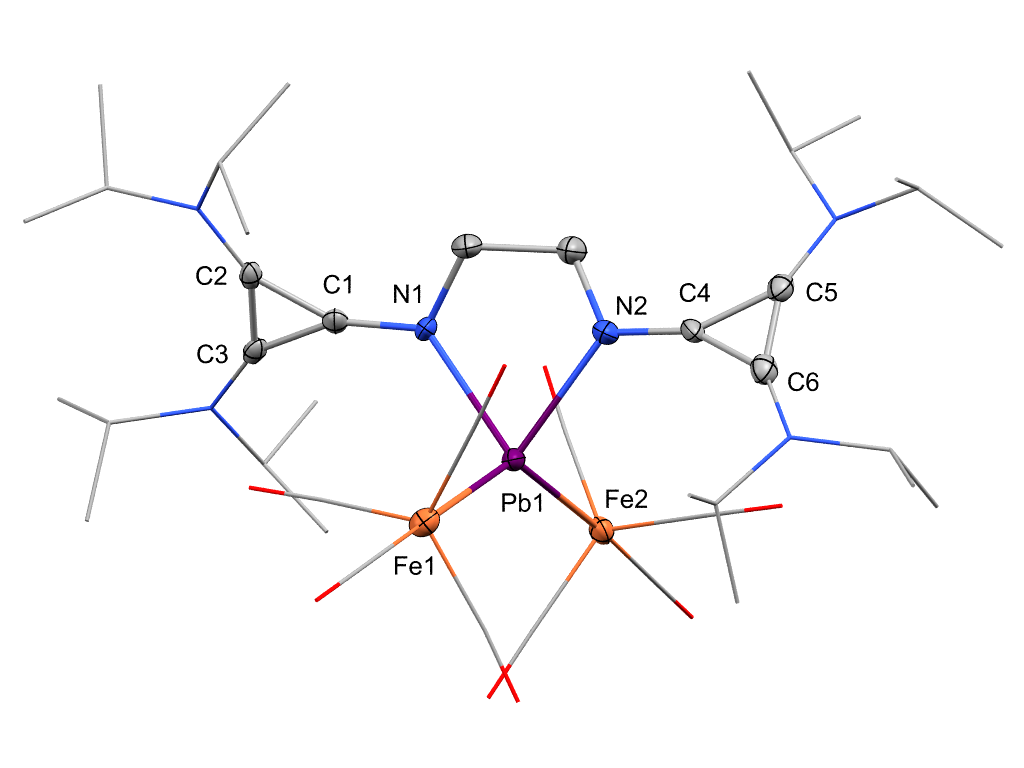


Figure S 59: The molecular structure of **9** thermal ellipsoids is set to a 50% probability level. Hydrogen atoms are omitted for clarity; isopropyl‑substituents and CO are depicted as wireframes for simplicity.

# 3 Computational Details

**General**

All the calculations were performed using the Gaussian 16 software package.^[20]^ Geometry optimization of the compounds was conducted at the PBE0^[21]^-D3(BJ)^[22]^ density functional theory level, according to the best agreement with the metric data from X-ray structure analyses (Table S1). The def2-SVP^[23]^ basis set describes C, O, N, H, Cl, and Br atoms, whereas the ma-TZVP^[24-25]^ basis set is used to describe Ge, Sn, and Pb atoms. The basis set ma-TZVP is the abbreviation of def2-TZVP with minimal augmentation, proposed by Truhlar and co-workers. In addition, frequency calculations are carried out at the same level of theory to confirm that the stationary points are minima with no imaginary frequencies.The electron density of delocalized bonds (EDDB) calculations were performed at the CAM-B3LYP^[26]^/def2-TZVP level using the RunEDDB script program.Viewing of optimized structures and rendering of molecular orbitals were performed using the program *CYLview^[27]^* and *VMD^[28]^*, respectively. Density matrices of natural atomic orbitals (NAO) used for Wiberg bond indices (WBI)^[29]^ analysis were obtained using the NBO 7.0 program ^[30-31]^.

**Optimized Structures**

Table S 5 Key distances (Å) of experimental and DFT-optimized structures of compound **8**.

| Functional | Exp. | TPSS | B3PW91 | PBE0 | B3LYP |
| --- | --- | --- | --- | --- | --- |
| Sn1-Fe2 | 2.535 | 2.547 | 2.533 | 2.533 | 2.572 |
| Sn1-Fe3 | 2.537 | 2.546 | 2.533 | 2.533 | 2.572 |
| Sn1-N13 | 2.183 | 2.209 | 2.190 | 2.198 | 2.211 |
| N13-C19 | 1.316 | 1.318 | 1.309 | 1.308 | 1.311 |
| N13-C25 | 1.482 | 1.462 | 1.447 | 1.447 | 1.455 |
| C25-C28 | 1.482 | 1.528 | 1.519 | 1.517 | 1.524 |
| C28-N14 | 1.480 | 1.462 | 1.447 | 1.447 | 1.455 |
| N14-C22 | 1.316 | 1.318 | 1.309 | 1.308 | 1.311 |
| N14-Sn | 2.176 | 2.209 | 2.190 | 2.198 | 2.211 |
| C19-C20 | 1.395 | 1.410 | 1.399 | 1.399 | 1.402 |
| C19-C21 | 1.388 | 1.408 | 1.398 | 1.399 | 1.401 |
| C21-C20 | 1.384 | 1.391 | 1.380 | 1.380 | 1.381 |
| C22-C23 | 1.396 | 1.41 | 1.399 | 1.388 | 1.402 |
| C22-C24 | 1.404 | 1.408 | 1.380 | 1.399 | 1.401 |
| C23-C24 | 1.376 | 1.391 | 1.380 | 1.380 | 1.381 |
| ∠N13-Sn-N14 | 77.5 | 77.0 | 77.3 | 77.2 | 77.1 |
| ∠Fe2-Sn-Fe3 | 128 | 143.1 | 143.1 | 141.7 | 143.0 |
| ∠Sn1-N13-C25 | 110.7 | 111.7 | 111.6 | 111.3 | 111.5 |
| ∠N13-N14-C28-C25 | 29.2 | 27.1 | 26.7 | 26.9 | 26.6 |
| RD(%)*^a^* | 0.00 | 1.87 | 1.80 | 1.69 | 1.97 |

*^a^* RD = $\frac{\sum_{i=1}^{n} \frac{\mid BL(DFT) - BL(Exp)\mid}{BL(Exp)}*100\%}{n}$, BL means bond length.

Figure S 60: Optimized geometries of **L^CPI^**, **1** - **9** and **1-0**, **2-0** and **3-0** computed at PBE0-D3(BJ)/def2-SVP~ma-TZVP level. Bond lengths (black) are in Å; and Wiberg bond index (WBI, blue).

Figure S 61: Optimized geometries of L, 1-9, 1-0, 2-0 and 3-0 computed at PBE0-D3(BJ)/def2-SVP~ma-TZVP level, Mayer bond order (MBO, blue); natural population analysis (NPA, red).

Figure S 62: Comparison of NPA values between compounds **4**-**9** and reported compounds **Fe(CO)_5_**, **SnFe_2_**, and **PbFe** (blue: Sum of the charges on the ligand scaffold, black: charges on the central element, red: charges on Fe, purple: charges on the Fe(CO)_4_ moiety).

Table S 6: Comparison of relative electronic energies (ΔE_ST_ = E_Triplet_ - E_Singlet_; kcal/mol) for different spin states of **L^CPI^** and **1**~**9** computed at (U)PBE0-D3(BJ)/def2-TZVP // PBE0-D3(BJ)/def2-SVP~ma-TZVP level.

| Entry | **L^CPI^** | **1** | **2** | **3** | **4** | **5** | **6** | **7** | **8** | **9** |
| --- | --- | --- | --- | --- | --- | --- | --- | --- | --- | --- |
| Δ*E*_ST_ | +56.7 | +67.8 | +56.7 | +65.0 | +41.5 | +40.2 | +37.0 | +38.2 | +34.8 | +34.2 |

According to the calculation results within the same main group, the binding energies between zero valent compounds and Fe(CO)_4_ gradually decrease from Ge to Pb (Table S 7)

Table S 7: Comparison of the bonding energies (*E*_b_, kcal mol^-1^) for tetrylone complexes **4** to **9** computed at (U)PBE0-D3(BJ)/def2-TZVP // PBE0-D3(BJ)/def2-SVP~ma-TZVP level.

| **complexes** | **4** | **5** | **6** | **7** | **8** | **9** |
| --- | --- | --- | --- | --- | --- | --- |
| *E*_b_ | -127.0 | -122.8 | -120.2 | -227.5 | -198.3 | -179.3 |

The calculation results indicate that the vibration frequency of CO in a zero-valent compound containing only one Fe(CO)_4_ unit is slightly lower than that of CO in a zero-valent compound containing two Fe(CO)_4_ units (Table S 8).

Table S 8: IR wavenumbers (*ν*, cm^-1^) of CO in tetrylone complexes **4** to **9** computed at PBE0-D3(BJ)/def2-SVP~ma-TZVP level.

| **complexes** | **4** | **7** | **5** | **8** | **6** | **9** |
| --- | --- | --- | --- | --- | --- | --- |
| *ν*_CO_ | 1956, 1980, 2036, 2108 | 2000, 2009, 2030, 2035, 2063, 2076, 2122, 2145 | 1959, 1984, 2029, 2101 | 2014, 2020, 2026, 2045, 2062, 2085, 2129, 2152 | 1961, 1983, 2024, 2099 | 2020, 2025, 2047, 2053, 2066, 2088, 2125, 2153 |

**Natural Resonance Theory**

The simplified model with zero-valence was used to perform NRT analysis, and the results showed that the first Lewis structural with a neutral and uncharged state had the highest weight.

Figure S 63. The natural resonance theory (NRT) analyses of tetrylone complexes stabilized by simplified **L^CPI^** (replace *i*Pr_2_N with H) computed at PBE0-D3(BJ)/def2-SVP~ma-TZVP level. Due to the relatively small weights of other resonance structures, only the top three weights are listed here.

**Aromaticity**

As shown in Table S 9 the electron density of delocalized bonds (EDDB) is used to further quantify the aromaticity of the three-membered rings (3MRs).^[32-33]^ The calculation results indicate that the EDDB values of the 3MRs in isolated ligand **L^CPI^** (0.844, 0.829e) are slightly smaller than that in the corresponding model compounds formed by its coordination with the metals [Ge (**1-0**), Sn (**2-0**), Pb (**3-0**) (Figure S 64); EDDBs: from 0.848 to 0.957e]. The EDDB values of the 3MRs in these compounds (**1** - **9**; EDDBs: ranging from 1.066 to 1.329e) that can be stably separated experimentally are more significant, supporting their thermodynamic stability.

Figure S 64: Overview of the isolated complexes 1 to 9, and the free tetrylones 1-0 (germylone), 2-0 (stannylone) and 3-0 (plumbylone).

Table S 9: Comparison of the EDDB values (e, A larger EDDB value indicates a more significant aromaticity.) of the three-membered rings (3MRs) in compounds **L^CPI^**, **1-0 - 3-0,** and **1** - **9** computed at CAM-B3LYP/def2-TZVP level.

| **complexes** | **L^CPI^** | **1-0** | **2-0** | **3-0** | **1** | **2** | **3** |
| --- | --- | --- | --- | --- | --- | --- | --- |
| EDDB_3MR_  (left/right) | 0.844/  0.829 | 0.848/  0.874 | 0.957/  0.957 | 0.924/  0.938 | 1.251/  1.263 | 1.222/  1.222 | 1.202/  1.176 |
| **complexes** | **4** | **5** | **6** | **7** | **8** | **9** |  |
| EDDB_3MR_  (left/right) | 1.146/  1.193 | 1.109/  1.297 | 1.257/  1.066 | 1.286/  1.329 | 1.183/  1.183 | 1.211/  1.211 |  |

**Dissociation Energies**

As shown in Scheme S 1, the germanium (**1-0**)/tin (**2-0**)/lead (**3-0**) atom is strongly bonded to the chelating ligand **L^CPI^** with a calculated bond dissociation energy of *D*e = 56.6/49.0/40.5 kcal/mol (Δ*G*^298^ = 46.2/38.6/31.1 kcal/mol). The Fe(CO)_4_ fragment in complexes **4**-**6** has a calculated bond strength at room temperature (RT) of 113.8/107.0/107.6 kcal/mol. The second Fe(CO)_4_ ligand is calculated with a bond strength of 77.9, 74.0, and 59.2 kcal/mol in complexes **7**, **8**, and **9**.

Scheme S 1: The bond dissociation energies (*D*e, kcal/mol) and free energy changes (∆*G*^298K^, kcal/mol) at 298 K computed at the PBE0-D3BJ/def2-TZVP // PBE0-D3(BJ)/def2-SVP~ma-TZVP level.

**Natural Bond Orbital Analysis**

Figure S 65: The key orbitals in natural bond orbital analysis (NBO) of germylone **1-0**. The number of electrons occupied is listed below the plot. The isosurface 0.050 a.u. is plotted.

Figure S 66: The key orbitals in natural bond orbital analysis (NBO) of stannylone **2-0**. The number of electrons occupied is listed below the plot. The isosurface 0.050 a.u. is plotted.

**Table S9**. Natural bond orbital analysis (NBO) of **3-0**

NATURAL BOND ORBITAL ANALYSIS

======================================================

(a) **3-0**

(Occupancy) Bond orbital / Coefficients / Hybrids

54. (1.98320) LP ( 1)Pb 99 s( 95.70%)p 0.04( 4.29%)d 0.00( 0.01%)

f 0.00( 0.00%)

0.0000 0.9782 -0.0014 0.0000 -0.0002

0.0000 0.0000 0.0000 -0.0156 0.0000

-0.0008 -0.0004 0.0000 0.0000 -0.1181

-0.0183 -0.0001 -0.0039 -0.0002 0.0000

0.1683 0.0034 0.0014 0.0006 0.0001

0.0000 0.0002 0.0001 0.0000 -0.0006

0.0007 0.0000 0.0016 -0.0009 0.0000

0.0071 0.0025 0.0000 -0.0072 -0.0030

-0.0028 0.0004 0.0010 0.0002 0.0011

-0.0014 -0.0010 0.0000 -0.0004 0.0003

-0.0003 -0.0003 -0.0042 -0.0008

55. (1.88468) LP ( 2)Pb 99 s( 1.42%)p69.43( 98.54%)d 0.01( 0.01%)

f 0.02( 0.02%)

0.0000 0.1191 0.0009 0.0013 0.0015

-0.0001 0.0000 0.0000 0.0858 0.0016

0.0019 0.0019 0.0001 0.0000 -0.3463

0.0137 -0.0063 0.0039 -0.0001 0.0000

-0.9261 0.0164 0.0035 -0.0019 -0.0001

0.0000 -0.0004 0.0000 0.0000 -0.0022

0.0001 0.0000 0.0109 0.0007 0.0000

-0.0021 -0.0026 0.0000 0.0027 0.0007

0.0094 0.0056 -0.0020 -0.0011 0.0072

0.0039 -0.0044 -0.0022 -0.0014 -0.0006

0.0011 0.0006 -0.0011 -0.0005

59. (1.96725) BD ( 2) C 1- N 93

( 30.10%) 0.5486* C 1 s( 0.00%)p 1.00( 99.81%)d 0.00( 0.19%)

0.0000 -0.0037 0.0033 0.0464 -0.0046

0.1115 -0.0014 0.9916 -0.0083 0.0088

0.0428 -0.0005 0.0019 -0.0019

( 69.90%) 0.8361* N 93 s( 0.24%)p99.99( 99.67%)d 0.39( 0.09%)

0.0000 0.0483 -0.0054 0.0544 0.0047

0.1525 -0.0053 0.9849 -0.0182 -0.0032

-0.0260 0.0148 -0.0034 -0.0032

61. (1.60340) BD ( 2) C 2- C 3

( 51.30%) 0.7162* C 2 s( 0.01%)p 1.00( 99.77%)d 0.00( 0.22%)

0.0000 0.0071 -0.0022 0.0669 -0.0033

0.0675 -0.0037 0.9942 0.0164 -0.0002

0.0214 -0.0415 0.0043 0.0036

( 48.70%) 0.6979* C 3 s( 0.00%)p 1.00( 99.76%)d 0.00( 0.24%)

0.0000 0.0039 -0.0006 0.0495 0.0095

0.0772 -0.0016 0.9943 0.0199 0.0046

0.0143 0.0454 -0.0032 -0.0081

67. (1.96492) BD ( 2) C 4- N 94

( 30.01%) 0.5478* C 4 s( 0.22%)p99.99( 99.59%)d 0.87( 0.19%)

0.0000 0.0464 0.0053 0.0043 -0.0042

-0.1476 -0.0020 0.9869 -0.0089 0.0052

-0.0430 -0.0005 -0.0015 0.0043

( 69.99%) 0.8366* N 94 s( 0.40%)p99.99( 99.51%)d 0.22( 0.09%)

0.0000 0.0633 0.0011 0.1258 -0.0062

-0.1603 -0.0040 0.9763 -0.0176 -0.0015

0.0254 0.0143 0.0057 -0.0013

69. (1.60009) BD ( 2) C 5- C 6

( 51.44%) 0.7172* C 5 s( 0.00%)p 1.00( 99.78%)d 0.00( 0.22%)

0.0000 0.0044 0.0000 0.0372 0.0023

-0.1711 -0.0027 0.9833 0.0157 0.0020

-0.0220 -0.0398 -0.0077 -0.0093

( 48.56%) 0.6969* C 6 s( 0.00%)p 1.00( 99.76%)d 0.00( 0.24%)

0.0000 0.0011 -0.0017 0.0468 -0.0042

-0.1459 -0.0030 0.9867 0.0210 0.0034

-0.0137 0.0444 0.0057 0.0139

======================================================

**Frontier Molecular Orbitals**

**Figure S 67:** The key frontier molecular orbitals of compounds **L^CPI^**, Hydrogen atoms in 3D structures are omitted for clarity. The highest occupied molecular orbital (HOMO) is the lowest unoccupied molecular orbital (LUMO). (isovalue = 0.04 a.u.)

Figure S 68: The key frontier molecular orbitals of compounds **1-0**, **2-0**, and **3-0**, Hydrogen atoms in 3D structures are omitted for clarity. The highest occupied molecular orbital (HOMO) is the lowest unoccupied molecular orbital (LUMO). (isovalue = 0.04 a.u.)

**Figure S 69:** The key frontier molecular orbitals of the precursor compounds **1**, **2**, and **3**, Hydrogen atoms in 3D structures are omitted for clarity. The highest occupied molecular orbital (HOMO) is the lowest unoccupied molecular orbital (LUMO). (isovalue = 0.04 a.u.)

**Figure S 70:** The key frontier molecular orbitals of the tetracarbonyl iron complexes **4**, **5**, and **6**, Hydrogen atoms in 3D structures are omitted for clarity. The highest occupied molecular orbital (HOMO) is the lowest unoccupied molecular orbital (LUMO). (isovalue = 0.04 a.u.)

**Figure S 71** The key frontier molecular orbitals of the bis- tetracarbonyl iron complexes **7**, **8**, and **9**, Hydrogen atoms in 3D structures are omitted for clarity. The highest occupied molecular orbital (HOMO) is the lowest unoccupied molecular orbital (LUMO). (isovalue = 0.04 a.u.)

**Cartesian Coordinates**

**L^CPI^**

PBE0-D3(BJ)/def2-SVP∼ma-TZVP

E = -1580.992185 a.u.

C 2.66519800 -0.68001500 0.33805700

C 3.22483400 0.61864000 0.17657200

C 4.04618000 -0.47875200 0.16248900

C 0.39984300 -0.94171900 0.60614100

H 0.36847200 -0.07266700 1.29747500

H -0.29985300 -1.67924100 1.03902800

C 1.63854000 2.45782200 0.15393900

H 0.99810500 1.58117000 0.30663500

C 1.43290900 3.37873200 1.35085500

H 0.37360300 3.66708400 1.42503400

H 2.02335500 4.30608600 1.26814000

H 1.71680200 2.87159200 2.28499600

C 1.19292000 3.09919900 -1.15373200

H 0.12191400 3.34147900 -1.10156800

H 1.33257100 2.40183600 -1.99247300

H 1.74751400 4.02674700 -1.37283500

C 5.08287900 2.88921900 1.04140000

H 5.91030100 3.59345300 0.86566800

H 5.51529500 1.89308200 1.21464700

H 4.56465800 3.18529400 1.96501500

C 6.48642500 -0.43162000 -0.14764700

H 6.22876200 0.63502900 -0.19174400

C 7.44696300 -0.61660400 1.02318400

H 8.36113500 -0.02189000 0.87343500

H 7.75111400 -1.66900700 1.13536500

H 6.97595500 -0.29808200 1.96495200

C 7.12262100 -0.79947800 -1.48473600

H 8.03329200 -0.20581700 -1.65824900

H 6.42267800 -0.61091700 -2.31228200

H 7.40849000 -1.86239500 -1.51829200

C 5.18938700 -2.58546800 0.17602100

H 6.23701500 -2.91138700 0.07369800

C 4.67483100 -3.02977300 1.54159400

H 4.75823300 -4.12277000 1.64437100

H 3.61191700 -2.75851800 1.65090300

H 5.25246600 -2.56126400 2.35272800

C 4.37444500 -3.20974200 -0.95262700

H 4.44925800 -4.30760900 -0.91628600

H 4.73629100 -2.87111200 -1.93522500

H 3.31154800 -2.93756800 -0.84502700

N 1.71424200 -1.52684600 0.51391800

N 3.01063300 1.94998600 0.08680200

N 5.22561400 -1.12310400 0.06649400

C 4.10761400 2.88289500 -0.13229000

H 3.64073100 3.87958400 -0.16368400

C 4.77479200 2.66561100 -1.48797200

H 5.60360900 3.37382800 -1.63984200

H 4.04528500 2.80634200 -2.29845400

H 5.17662900 1.64656400 -1.58335700

C -2.40503100 0.08250600 -0.39043800

C -3.43821700 -0.87982700 -0.22357200

C -3.74423900 0.45245400 -0.15312400

C -0.15309300 -0.45750300 -0.74363600

H -0.43463600 -1.33787600 -1.35955200

H 0.68068300 0.01820100 -1.28979600

C -2.70815800 -3.18415300 -0.30711300

H -1.80007000 -2.60371600 -0.52010100

C -2.94777300 -4.11006100 -1.49402800

H -2.09293100 -4.78994600 -1.62648700

H -3.84478800 -4.73492100 -1.35448400

H -3.07267700 -3.52978800 -2.42001600

C -2.46716600 -3.94956900 0.98903400

H -1.59649500 -4.61412200 0.88469300

H -2.26547300 -3.25476800 1.81735700

H -3.33234700 -4.57519900 1.26307300

C -6.08942500 -2.21886300 -1.00896100

H -7.11700000 -2.54576800 -0.78887300

H -6.10359300 -1.12912900 -1.15640000

H -5.77630400 -2.67327600 -1.96007700

C -5.98671500 1.37534200 0.25691500

H -6.17631800 0.29359000 0.26930800

C -6.81933000 1.96765100 -0.87591900

H -7.89141400 1.78373400 -0.70709600

H -6.67889100 3.05697600 -0.95517000

H -6.53791200 1.51870300 -1.84002000

C -6.39035300 1.92205400 1.62268000

H -7.45797500 1.73569400 1.81534800

H -5.80500300 1.44285000 2.42145200

H -6.22710300 3.00909200 1.68834000

C -3.94290200 2.84060000 -0.06485500

H -4.76838400 3.55462100 0.08594400

C -3.33028000 3.09158900 -1.43902200

H -2.96052100 4.12629900 -1.51009000

H -2.47747500 2.41213300 -1.60153500

H -4.07179000 2.93496900 -2.23680000

C -2.91767200 3.04479000 1.04653400

H -2.53724500 4.07813200 1.03218700

H -3.36192800 2.85447100 2.03502300

H -2.06376200 2.36362500 0.89900500

N -1.21233200 0.50650800 -0.59920600

N -3.77073600 -2.18879500 -0.17041900

N -4.55919600 1.51180500 0.01361300

C -5.13334300 -2.62134600 0.11004300

H -5.09498900 -3.72182300 0.11687100

C -5.59382700 -2.18652100 1.49884600

H -6.62307900 -2.52237300 1.69656700

H -4.93643700 -2.61160400 2.27087500

H -5.56601000 -1.09314600 1.61148700

**1-0**

PBE0-D3(BJ)/def2-SVP∼ma-TZVP

E = -3657.700991 a.u.

C 2.63727600 0.16518800 -0.08550000

C 3.86738600 0.82779100 0.11601200

C 3.86518200 -0.53115700 -0.07899600

C -2.63208700 0.13248600 -0.22685400

C -3.90035000 0.76042100 -0.18462000

C -3.81364200 -0.58202100 0.07617500

C -0.65279800 1.28933700 -0.84574500

H -1.24707000 2.20860200 -0.73215600

H -0.43145500 1.15142000 -1.92109100

C 3.64929000 3.15610700 0.72159400

H 2.66561500 2.73128700 0.96175400

C 4.15008100 3.84829100 1.98391600

H 3.44831400 4.64204400 2.28050100

H 5.13345000 4.32192200 1.83592900

H 4.22976800 3.13384500 2.81602000

C 3.47292900 4.12738600 -0.44113300

H 2.77542700 4.93548300 -0.17294900

H 3.07406000 3.60748400 -1.32505400

H 4.42854600 4.59711600 -0.72465300

C 5.90547600 2.21889900 0.11293200

H 6.03795700 3.31249600 0.10552400

C 6.76857400 1.67961000 1.25166800

H 7.83793300 1.83716000 1.04355100

H 6.60800400 0.60410500 1.41125100

H 6.52402700 2.18612200 2.19565300

C 6.32734800 1.72735300 -1.26684100

H 7.39258100 1.93756600 -1.44386000

H 5.73932700 2.23098100 -2.04793300

H 6.17354700 0.64607200 -1.38533300

C 5.95483700 -1.77408700 -0.05182200

H 6.27649400 -0.74553500 0.15280000

C 6.32467900 -2.61495200 1.16525000

H 7.41153900 -2.58418800 1.33756400

H 6.03986000 -3.66966500 1.02936400

H 5.81876800 -2.23886400 2.06657200

C 6.68067700 -2.22946600 -1.31372100

H 7.77023200 -2.17588600 -1.16782700

H 6.41333800 -1.59436300 -2.17116600

H 6.43380300 -3.26957400 -1.57550800

C 3.76097600 -2.91764100 -0.56150100

H 4.53725400 -3.68025400 -0.73262700

C 2.91924100 -3.37291300 0.62231100

H 2.35894900 -4.28445700 0.36776100

H 2.17433800 -2.61479100 0.91985100

H 3.55153200 -3.58301600 1.49767700

C 2.97137100 -2.77123100 -1.85363100

H 2.55541900 -3.74442900 -2.15286000

H 3.62023400 -2.40443700 -2.66425400

H 2.10296900 -2.09752600 -1.73270500

C -4.13316300 3.20204400 -0.16265500

H -5.00397100 3.84741500 0.03652300

C -3.22889900 3.30262500 1.06060000

H -2.86891700 4.33417300 1.18965800

H -2.35174000 2.64466000 0.98173300

H -3.77695200 3.01140000 1.96872700

C -3.48672500 3.70935900 -1.44833400

H -3.07622500 4.72133400 -1.30916100

H -4.22277900 3.74663600 -2.26389200

H -2.66933400 3.05067400 -1.77226500

C -6.13268100 1.68586500 -0.37425700

H -6.28917200 0.62198600 -0.60183200

C -6.79459900 1.98882600 0.96747100

H -7.86978300 1.75510900 0.93662000

H -6.69587200 3.05344000 1.23256200

H -6.33537200 1.39776500 1.77423900

C -6.76309000 2.47287100 -1.51736400

H -7.83857100 2.24903000 -1.58181300

H -6.29710500 2.20794300 -2.47741900

H -6.66410200 3.56007700 -1.37346700

C -5.84415100 -1.73782600 0.69293600

H -6.07837500 -0.69096600 0.93299100

C -6.12687100 -2.54478000 1.95479200

H -7.18520900 -2.44094100 2.23811200

H -5.50672300 -2.19133600 2.79089800

H -5.92786800 -3.61744700 1.81010700

C -6.72495800 -2.16654200 -0.47743800

H -7.79156200 -2.07088400 -0.22221700

H -6.54094600 -3.21714800 -0.75115900

H -6.52373600 -1.55044200 -1.36708600

C -3.74907500 -3.03132600 0.11904100

H -4.56404700 -3.77370500 0.10476300

C -2.81479800 -3.37683400 1.26881700

H -2.33418400 -4.35109100 1.09320800

H -3.36124900 -3.42625100 2.22184600

H -2.00646400 -2.62937700 1.37732000

C -3.07398900 -3.08489900 -1.24379800

H -2.72139600 -4.10697100 -1.44579300

H -2.17981600 -2.43706800 -1.29907200

H -3.77787800 -2.79770000 -2.04047900

Ge -0.00232700 -1.41389400 0.08150400

N 1.34280800 0.16450600 -0.14198900

N -1.34390300 0.12660300 -0.34196400

N 4.48030400 2.01007300 0.35158600

N 4.51315500 -1.69189300 -0.24976900

N -4.68286100 1.85505000 -0.32369300

N -4.42860500 -1.74403600 0.33794400

C 0.65424400 1.43394700 -0.09382900

H 1.25005800 2.23894300 -0.55882400

H 0.43656700 1.71010900 0.95759200

**1**

PBE0-D3(BJ)/def2-SVP∼ma-TZVP

E = -4117.625122 a.u.

C -2.60880800 0.26205600 -0.06444800

C -3.84731500 0.88086600 -0.22624300

C -3.77861600 -0.50147600 -0.09854700

C 2.61260800 0.25953100 -0.02481100

C 3.89676900 0.80997700 -0.03035100

C 3.72820800 -0.54842000 -0.25547100

C 0.70937600 1.61683100 0.56868300

H 1.27609300 2.50102200 0.25026300

H 0.68612500 1.59606500 1.67227900

C -3.75657200 3.26782300 -0.58069600

H -2.73381500 2.94488500 -0.81114800

C -4.24575800 4.05971100 -1.78622900

H -3.58589000 4.92341100 -1.95329200

H -5.26305800 4.45481200 -1.64441000

H -4.23659200 3.44185900 -2.69568600

C -3.72126100 4.09850800 0.69578600

H -3.08062200 4.98312000 0.56447900

H -3.32992300 3.51112200 1.54029500

H -4.72476600 4.45957300 0.97027400

C -5.97633400 2.11625600 -0.24171700

H -6.18367200 3.19615100 -0.22148200

C -6.71246000 1.55480300 -1.45316300

H -7.80102500 1.62021200 -1.31003100

H -6.45986500 0.50305600 -1.64616800

H -6.45334400 2.12231300 -2.35787800

C -6.43825300 1.57343400 1.10531500

H -7.52680000 1.68741900 1.21085100

H -5.95564600 2.12438200 1.92546000

H -6.20051700 0.50943300 1.24010600

C -5.83832300 -1.79633100 -0.19587800

H -6.20033900 -0.76424000 -0.25356400

C -6.22123400 -2.48786500 -1.49775200

H -7.31388200 -2.47578600 -1.62383000

H -5.90175000 -3.54105400 -1.51369300

H -5.77040100 -1.97955400 -2.36307500

C -6.48410000 -2.43515000 1.02638700

H -7.57921300 -2.41312900 0.92707700

H -6.21072400 -1.89633600 1.94556000

H -6.18881500 -3.48832600 1.14793000

C -3.60101800 -2.92744700 0.07849200

H -4.35458900 -3.72840900 0.07030900

C -2.71046200 -3.14218200 -1.13737300

H -2.17121700 -4.09678200 -1.05216600

H -1.95183200 -2.35177400 -1.23409000

H -3.30837900 -3.16332600 -2.05994000

C -2.88154900 -3.00089500 1.41536800

H -2.30394700 -3.93330900 1.49250600

H -3.60284600 -2.97387900 2.24508800

H -2.17845000 -2.16990600 1.56854900

C 4.27285100 3.22948300 0.02029900

H 5.18958300 3.82707400 -0.09412300

C 3.43915100 3.47652000 -1.23087500

H 3.12502300 4.52902900 -1.28107700

H 2.53350900 2.85330100 -1.26044700

H 4.02465300 3.25029600 -2.13391000

C 3.61049300 3.66773300 1.32107700

H 3.25697400 4.70658900 1.24445000

H 4.32090400 3.61251900 2.15729900

H 2.75157900 3.03394400 1.57805400

C 6.18329800 1.58131200 0.19934200

H 6.26818700 0.50230700 0.38751500

C 6.91568200 1.90764700 -1.09581000

H 7.97820700 1.63374600 -1.01859900

H 6.87060500 2.98384200 -1.32424800

H 6.48037800 1.36371100 -1.94789400

C 6.79195800 2.27894400 1.40874200

H 7.84480000 1.97984700 1.51511800

H 6.26399300 2.00305400 2.33274400

H 6.77544700 3.37497800 1.31088700

C 5.70861700 -1.79155100 -0.88798100

H 6.00941400 -0.74930600 -1.05879800

C 5.91806200 -2.52338100 -2.20751200

H 6.97653200 -2.46168500 -2.49945200

H 5.31526400 -2.07402100 -3.00977800

H 5.66251500 -3.59131200 -2.13785100

C 6.55665700 -2.36039900 0.24179600

H 7.62358100 -2.33129600 -0.02428800

H 6.29822600 -3.41003900 0.45029800

H 6.41609900 -1.78827000 1.17138600

C 3.52906400 -2.98156800 -0.39217500

H 4.28221000 -3.77338600 -0.51891000

C 2.51657600 -3.13297000 -1.51873800

H 1.98004000 -4.08867800 -1.42731300

H 3.01415800 -3.10767300 -2.49852900

H 1.76105500 -2.33407400 -1.49169200

C 2.94939800 -3.14054400 1.00530300

H 2.40575700 -4.09246300 1.08904500

H 2.24238300 -2.34039200 1.26929100

H 3.75002600 -3.13422200 1.75948200

Cl 0.06223800 -0.98920000 2.55116000

Ge 0.00982600 -1.11131300 0.26658900

N -1.29703100 0.37216900 0.05284300

N 1.30012700 0.38575700 0.07141400

N -4.51847700 2.02463500 -0.39504800

N -4.38333300 -1.68610400 -0.04299100

N 4.74032400 1.83842300 0.08281500

N 4.28844500 -1.72691100 -0.51540100

C -0.70497300 1.70232600 0.02626200

H -1.28147900 2.41065100 0.64248800

H -0.68314700 2.09006300 -1.01024200

**2-0**

PBE0-D3(BJ)/def2-SVP∼ma-TZVP

E = -1795.296674 a.u.

Sn 0.00000200 -1.44233400 -0.00006600

N 1.39502200 0.39400300 -0.04729900

N -1.39503100 0.39399900 0.04716100

N 4.82049700 1.88145200 -0.52489300

N 4.24677400 -1.62686700 0.67184600

C 2.68808600 0.37029600 0.00301700

C 3.99754600 0.87355600 -0.15961100

C 3.82150600 -0.42099000 0.26768300

C -2.68809600 0.37029700 -0.00310100

C -3.99754000 0.87357100 0.15961500

N -4.82046800 1.88149000 0.52487700

C -3.82154000 -0.42099000 -0.26765200

N -4.24681000 -1.62686900 -0.67180600

C 0.65592400 1.58525000 -0.38424700

H 0.41968500 1.59831600 -1.46679200

H 1.21250400 2.50513300 -0.14299900

C -0.65593800 1.58526300 0.38406500

H -0.41969800 1.59837300 1.46661100

H -1.21252700 2.50513300 0.14278100

C 4.27301500 3.15888600 -0.98014700

H 5.14924200 3.75326100 -1.28224900

C 3.59378100 3.91724700 0.15644900

H 3.15982800 4.86203600 -0.20409100

H 4.31634400 4.15089700 0.95155000

H 2.78737400 3.32340200 0.60899400

C 3.40729300 2.98810000 -2.22208600

H 3.02628200 3.96093500 -2.56710600

H 2.54466000 2.33582900 -2.03338800

H 3.99334300 2.53784200 -3.03655100

C 6.26808300 1.72833400 -0.43842400

H 6.42785500 0.70472800 -0.08104700

C 6.93646500 1.83592700 -1.80510000

H 8.01129400 1.61350600 -1.72500600

H 6.84178400 2.84707100 -2.23085500

H 6.48847000 1.12526500 -2.51525200

C 6.88988700 2.66721700 0.58963000

H 7.96658500 2.46395900 0.69356300

H 6.41737400 2.53503600 1.57426500

H 6.78169500 3.72336600 0.29640900

C 5.64452900 -2.03950900 0.60133200

H 5.63519700 -3.10935800 0.85860900

C 6.19101700 -1.94721700 -0.81925200

H 7.23573400 -2.29069900 -0.85883300

H 6.15788900 -0.92174400 -1.21272500

H 5.59240100 -2.57501700 -1.49484000

C 6.50130600 -1.34548600 1.65714800

H 7.55358800 -1.65884000 1.57897800

H 6.14044200 -1.59661900 2.66453200

H 6.46560700 -0.25059800 1.56467100

C 3.24640900 -2.59761700 1.13419000

H 2.28980000 -2.04349400 1.20467500

C 3.54431800 -3.09252200 2.54216800

H 2.70537600 -3.71113600 2.89320800

H 3.65191500 -2.24858300 3.23946200

H 4.45701200 -3.70907000 2.58978400

C 3.05442500 -3.73472700 0.14195100

H 2.20926300 -4.36364000 0.46022800

H 3.94631500 -4.38074300 0.07332300

H 2.81743500 -3.34421700 -0.85853400

C -4.27296400 3.15891900 0.98011600

H -5.14917900 3.75331000 1.28221700

C -3.59372600 3.91723700 -0.15651100

H -3.15959100 4.86194500 0.20401700

H -4.31633300 4.15102700 -0.95153300

H -2.78745800 3.32327900 -0.60915900

C -3.40723400 2.98814300 2.22205100

H -3.02630400 3.96099500 2.56711400

H -2.54454700 2.33594800 2.03333100

H -3.99325600 2.53780500 3.03649200

C -6.26806100 1.72835800 0.43855300

H -6.42786700 0.70475800 0.08117700

C -6.88996200 2.66725700 -0.58942600

H -7.96667400 2.46401300 -0.69325300

H -6.41754900 2.53508400 -1.57411000

H -6.78173000 3.72340200 -0.29620500

C -6.93631000 1.83593100 1.80529700

H -8.01114300 1.61348700 1.72530400

H -6.84160800 2.84707600 2.23104700

H -6.48823300 1.12527200 2.51539900

C -5.64457600 -2.03947900 -0.60134500

H -5.63526900 -3.10931800 -0.85866900

C -6.19109600 -1.94722500 0.81923000

H -7.23585400 -2.29058200 0.85876500

H -6.15784700 -0.92178100 1.21277400

H -5.59256900 -2.57513900 1.49479300

C -6.50130100 -1.34540000 -1.65716500

H -7.55358000 -1.65879000 -1.57908400

H -6.14036300 -1.59646200 -2.66454100

H -6.46564500 -0.25051700 -1.56461600

C -3.24644000 -2.59763900 -1.13410600

H -2.28984800 -2.04350700 -1.20463200

C -3.05445800 -3.73469100 -0.14180300

H -2.20936000 -4.36368700 -0.46007800

H -3.94639000 -4.38064100 -0.07308200

H -2.81739300 -3.34412100 0.85864100

C -3.54435600 -3.09260500 -2.54206100

H -2.70541500 -3.71123200 -2.89308000

H -3.65195400 -2.24869300 -3.23938700

H -4.45705000 -3.70915400 -2.58965000

**2**

PBE0-D3(BJ)/def2-SVP∼ma-TZVP

E = -6943.229681 a.u.

Sn -0.00004700 -0.00078700 0.86690200

N 1.33218600 0.28326300 -0.87158700

Br 0.78981400 -2.77040800 0.64053100

Br -0.78995300 2.76929800 0.64547400

N -1.33218200 -0.28170000 -0.87215500

N 4.87514400 -0.72986300 -1.64605100

N 3.88865700 1.40368000 1.29159500

C 2.62700200 0.26948400 -0.67028400

C 3.96163700 -0.08068100 -0.91095400

C 3.63675100 0.69117000 0.19261200

C -2.62700100 -0.26829500 -0.67083600

C -3.96164200 0.08225300 -0.91087300

N -4.87510800 0.73287500 -1.64474500

C -3.63673100 -0.69161400 0.19127600

N -3.88863200 -1.40619700 1.28891200

C 0.73180600 -0.20020800 -2.09727400

H 0.80320200 -1.30065000 -2.17585100

H 1.21812400 0.24976800 -2.97874900

C -0.73178800 0.20409100 -2.09691200

H -0.80319600 1.30467700 -2.17342600

H -1.21809100 -0.24422900 -2.97924500

C 4.45733100 -1.65587900 -2.70737200

H 5.39125900 -2.12798700 -3.04899800

C 3.87436900 -0.91351100 -3.90215400

H 3.61948600 -1.61973900 -4.70628400

H 4.59325500 -0.18168800 -4.29865500

H 2.95746400 -0.37540100 -3.62954400

C 3.55891600 -2.75898900 -2.16701200

H 3.26264600 -3.44451300 -2.97464200

H 2.64213300 -2.37158700 -1.69873200

H 4.08083600 -3.34306400 -1.39549700

C 6.29788600 -0.60971500 -1.33732200

H 6.35094300 0.09271300 -0.49644400

C 6.89663400 -1.92996300 -0.86427300

H 7.93303700 -1.77786500 -0.52730700

H 6.91946400 -2.68217800 -1.66761300

H 6.31791200 -2.34723100 -0.02728200

C 7.07838600 0.00407500 -2.49355100

H 8.13029100 0.15601000 -2.20811500

H 6.65316600 0.97756500 -2.77894400

H 7.06887600 -0.64769800 -3.38115000

C 5.20009900 1.45084300 1.93458700

H 5.02728100 1.98364500 2.88172200

C 5.69353600 0.05666300 2.29809200

H 6.65668500 0.11161500 2.82685200

H 5.83390000 -0.57538700 1.41027600

H 4.96580100 -0.44856500 2.94914200

C 6.20305900 2.27987800 1.13745300

H 7.17683900 2.31036200 1.64925200

H 5.84396600 3.31148500 1.01847400

H 6.36512800 1.87239800 0.12897500

C 2.78180500 2.19163500 1.86305500

H 1.92062200 2.02931300 1.19667000

C 3.06225400 3.68628500 1.82471600

H 2.15367500 4.22833800 2.12471700

H 3.32228200 4.01051200 0.80645300

H 3.87612800 3.97691400 2.50913500

C 2.39767000 1.70461700 3.25202800

H 1.47608100 2.21235200 3.57191600

H 3.17892400 1.91941000 3.99929400

H 2.20175200 0.62198900 3.24980800

C -4.45721400 1.66070600 -2.70444800

H -5.39111700 2.13341300 -3.04531400

C -3.87425400 0.92034900 -3.90047600

H -3.61931800 1.62793600 -4.70339300

H -4.59318400 0.18924800 -4.29823200

H -2.95738100 0.38172000 -3.62877400

C -3.55878400 2.76286600 -2.16217000

H -3.26214400 3.44954900 -2.96867800

H -2.64221000 2.37462200 -1.69417100

H -4.08084600 3.34588100 -1.38995000

C -6.29783500 0.61255600 -1.33601300

H -6.35095300 -0.09131700 -0.49634400

C -7.07864300 0.00095500 -2.49319300

H -8.13053600 -0.15126000 -2.20786200

H -6.65366800 -0.97212300 -2.78034700

H -7.06913300 0.65427000 -3.37965800

C -6.89615500 1.93215500 -0.86063100

H -7.93259900 1.77980200 -0.52391100

H -6.91874800 2.68577800 -1.66265800

H -6.31728100 2.34778100 -0.02292800

C -5.20008600 -1.45460000 1.93179000

H -5.02732400 -1.98938700 2.87781500

C -5.69344100 -0.06114900 2.29818600

H -6.65659400 -0.11713700 2.82682900

H -5.83375500 0.57275800 1.41168800

H -4.96567500 0.44267600 2.95028800

C -6.20305200 -2.28192700 1.13289000

H -7.17689600 -2.31334300 1.64450800

H -5.84403800 -3.31333000 1.01188800

H -6.36495600 -1.87241200 0.12520700

C -2.78178300 -2.19519700 1.85891800

H -1.92057600 -2.03160100 1.19287600

C -2.39770700 -1.71082000 3.24883400

H -1.47611600 -2.21914100 3.56778800

H -3.17898500 -1.92706400 3.99565800

H -2.20181400 -0.62818600 3.24869200

C -3.06222900 -3.68977500 1.81773500

H -2.15366000 -4.23240700 2.11671900

H -3.32225700 -4.01206300 0.79885700

H -3.87611500 -3.98169500 2.50159200

**3-0**

PBE0-D3(BJ)/def2-SVP∼ma-TZVP

E = -1773.862500 a.u.

C -2.69755400 0.46447200 -0.24790200

C -3.98362900 1.05612500 -0.22434400

C -3.86806300 -0.30811800 -0.12075900

C 2.71475900 0.46282800 -0.04057500

C 4.00919800 1.03564000 -0.01684000

C 3.87288200 -0.31495000 -0.22356300

C -0.61638000 1.59849100 -0.57504100

H -1.16904400 2.53763500 -0.40747700

H -0.31453900 1.57783800 -1.64117600

C 0.64890400 1.63680300 0.26921900

H 1.20390900 2.55492600 0.01471200

H 0.35161100 1.70059900 1.33470800

C -4.14167600 3.49065800 -0.25132600

H -4.98365200 4.19804900 -0.19628400

C -3.41815100 3.76687700 -1.56602000

H -4.12204500 3.72050100 -2.40960700

H -2.62510500 3.03095500 -1.75437300

H -2.95794800 4.76659400 -1.55672500

C -3.29018000 3.71870800 0.99321500

H -2.82167800 4.71402100 0.97166100

H -2.49205500 2.96827100 1.08350100

H -3.91133100 3.64804000 1.89816200

C -6.21004000 2.05701100 -0.28415400

H -6.41913000 0.98185600 -0.23389100

C -6.87397100 2.70033200 0.92882500

H -6.73477600 3.79259000 0.94338800

H -6.46044300 2.29278900 1.86308700

H -7.95766300 2.50912500 0.91815800

C -6.78401400 2.56988700 -1.60068500

H -7.86993500 2.39479800 -1.64262900

H -6.31808100 2.05610600 -2.45452300

H -6.62019300 3.65248000 -1.72092700

C -5.73260300 -1.86169000 0.30253200

H -5.74545400 -2.94213300 0.51328000

C -6.66901500 -1.63407100 -0.88197500

H -7.71080900 -1.86160800 -0.60876000

H -6.38566800 -2.27879900 -1.72551100

H -6.63206600 -0.59582200 -1.24179500

C -6.17173100 -1.16763800 1.58655700

H -7.20660300 -1.44025400 1.84235100

H -6.12261300 -0.07293800 1.50248400

H -5.51685400 -1.46228400 2.41919700

C -3.39614200 -2.66913500 -0.19427900

H -2.44853300 -2.20709600 -0.52506500

C -3.81657500 -3.60948400 -1.31601300

H -4.74163400 -4.15838000 -1.07538600

H -3.02304600 -4.35184700 -1.48610800

H -3.96832100 -3.05811000 -2.25549800

C -3.13823800 -3.40586800 1.11292000

H -4.03109300 -3.95169000 1.46150500

H -2.81490400 -2.70779400 1.89906000

H -2.33474800 -4.14464500 0.97007200

C 4.22160000 3.43259400 0.40125800

H 5.08273100 4.10513300 0.53755300

C 3.43667300 3.45135000 1.70754400

H 3.01861900 4.45136400 1.89704000

H 4.09265000 3.18578500 2.54948200

H 2.60481400 2.73461300 1.69531000

C 3.44311400 3.94930000 -0.80551400

H 2.64059000 3.25588300 -1.09345400

H 4.10786300 4.06320300 -1.67398500

H 2.98671000 4.92718400 -0.59007200

C 6.25092800 2.00384800 -0.00458300

H 6.43894500 0.93896400 -0.18397400

C 6.76845500 2.76419900 -1.22103200

H 6.62654300 3.85114000 -1.11354100

H 6.24738000 2.43947700 -2.13387800

H 7.84598700 2.58531400 -1.35668200

C 6.98931100 2.38088000 1.27538300

H 6.86754000 3.44743400 1.52101300

H 8.06782900 2.19080800 1.16657400

H 6.61852100 1.79203400 2.12733400

C 5.73868800 -1.91641700 -0.35042400

H 5.75578600 -3.01476000 -0.41498600

C 6.53294500 -1.39695900 -1.54577300

H 6.46838400 -0.30344900 -1.64030000

H 6.13992500 -1.82649300 -2.47817500

H 7.59630600 -1.66835700 -1.46103300

C 6.33389800 -1.55755000 1.00712100

H 6.28115400 -0.47929700 1.21260700

H 7.39032900 -1.86057500 1.06282200

H 5.78310400 -2.07000200 1.80900300

C 3.33496800 -2.62186000 -0.64178200

H 2.36383900 -2.11084800 -0.79099600

C 3.59310100 -3.40775300 -1.91824900

H 2.74777000 -4.08840400 -2.09838800

H 4.50951100 -4.01813300 -1.86464500

H 3.66884400 -2.73317100 -2.78382200

C 3.19725800 -3.51894800 0.57984600

H 2.99812200 -2.92282900 1.48328500

H 4.09957200 -4.12899900 0.75363600

H 2.34915800 -4.20675100 0.43719100

N -1.40801700 0.43186300 -0.27641900

N 1.42673700 0.44343700 0.05514700

N -4.75689000 2.16448000 -0.23631100

N -4.34333000 -1.55977700 -0.02452200

N 4.80504400 2.11642100 0.14590000

N 4.32673000 -1.55928000 -0.43729700

Pb -0.03071100 -1.45801700 0.43095700

**3**

PBE0-D3(BJ)/def2-SVP∼ma-TZVP

E = -6921.796832 a.u.

C -2.50049000 -0.18249400 -0.79132200

C -3.51700800 0.74251300 -0.50124600

C -3.33583300 -0.35420800 0.31503400

C 2.20782400 0.64431000 -0.76929500

C 2.94796400 1.72669700 -0.27086400

C 3.32888400 0.43368500 0.03005300

C -0.89516200 -0.04805800 -2.59520100

H -1.61684300 0.56981000 -3.15374900

H -0.53705100 -0.83528700 -3.27824600

C 0.29165300 0.81662800 -2.18412900

H 0.77522000 1.19107100 -3.10598200

H -0.07727500 1.69383400 -1.62308400

C -3.93480700 2.61977500 -2.01681400

H -4.67362700 3.43553500 -2.00858700

C -4.22966100 1.76810600 -3.24624600

H -5.27635700 1.43069500 -3.24096700

H -3.59274600 0.87291000 -3.28249400

H -4.05665300 2.34231800 -4.16849900

C -2.55729600 3.26575800 -2.03038500

H -2.40923000 3.85055800 -2.95066100

H -1.76252300 2.51190000 -1.97678200

H -2.43581600 3.93522400 -1.16694800

C -5.24019300 2.34874600 0.11252100

H -5.22690100 1.65705800 0.96203200

C -4.93945500 3.73626700 0.66656700

H -4.93706200 4.50381200 -0.12322300

H -3.95796800 3.75011800 1.16300500

H -5.70399700 4.02613400 1.40292700

C -6.61869800 2.25550800 -0.53162200

H -7.39998100 2.52471000 0.19513600

H -6.81659600 1.23285900 -0.88600800

H -6.71851100 2.93906700 -1.38931200

C -4.48358700 -0.73958700 2.46000100

H -4.38000400 -1.54496400 3.20227500

C -5.95682800 -0.70096300 2.06325200

H -6.57986900 -0.39206900 2.91625400

H -6.29522000 -1.69318000 1.73247100

H -6.14672200 -0.00103500 1.23795800

C -3.96798700 0.53027400 3.12604400

H -4.57394700 0.77503100 4.01126200

H -3.99409600 1.39604700 2.44900300

H -2.91823500 0.40249100 3.42767600

C -2.99616700 -2.47685100 1.39382900

H -2.37015500 -2.52234300 0.48924100

C -4.03808600 -3.58170100 1.26566700

H -4.70653900 -3.61104000 2.14107300

H -3.54204100 -4.56142500 1.19843000

H -4.65199500 -3.44464200 0.36327800

C -2.09303800 -2.66259200 2.60548600

H -2.67055200 -2.70876900 3.54228800

H -1.36574300 -1.83908200 2.67952700

H -1.54480200 -3.61269500 2.51701200

C 2.12199600 4.00020800 -0.61796400

H 2.52012200 4.99049000 -0.34831500

C 0.79311700 3.83217500 0.10764900

H 0.04178300 4.52743900 -0.29639300

H 0.90942100 4.03215400 1.18206600

H 0.40188700 2.80717500 0.03122700

C 2.01136400 3.97339800 -2.13679700

H 1.67633700 2.99430600 -2.50106000

H 2.98429300 4.19250200 -2.60085000

H 1.28594100 4.72504300 -2.48279900

C 4.29700900 3.56639700 0.57379400

H 4.86033800 2.67814700 0.88432300

C 5.19999200 4.37927900 -0.34688300

H 4.69924200 5.29259800 -0.70487700

H 5.49752900 3.78621600 -1.22427200

H 6.11136000 4.69111800 0.18546900

C 3.91511000 4.32501500 1.83994700

H 3.37467600 5.25691100 1.61258000

H 4.81839400 4.59931900 2.40541900

H 3.27315900 3.70943400 2.48692100

C 5.10767500 -0.22014100 1.60168400

H 5.40127800 -1.20603200 1.99275200

C 6.36038800 0.40081500 0.99093100

H 6.14252000 1.35117600 0.48239500

H 6.80190200 -0.27429300 0.24435000

H 7.11524800 0.59886700 1.76693200

C 4.52695000 0.56218600 2.77266300

H 4.16490000 1.55320400 2.46738400

H 5.28587700 0.70633800 3.55607600

H 3.66841100 0.02780600 3.20362100

C 3.81731700 -1.90853900 0.23397000

H 3.03937500 -1.86901900 -0.54488100

C 5.02174300 -2.57804100 -0.41190400

H 4.73495300 -3.57717700 -0.77105800

H 5.85669800 -2.70013100 0.29707200

H 5.37537000 -1.99740000 -1.27620000

C 3.27753500 -2.70149300 1.41792200

H 2.43910000 -2.17391700 1.89860700

H 4.05209700 -2.87707100 2.18159800

H 2.92154700 -3.68409000 1.07433500

N -1.49660500 -0.69963200 -1.45121800

N 1.22262800 0.04888200 -1.39091400

N -4.18030500 1.88112700 -0.77699100

N -3.62088000 -1.14722400 1.35222500

N 3.12776700 3.04963500 -0.13202800

N 4.09025800 -0.50833800 0.59293900

Br -0.04511700 0.47691500 1.87763000

Br 1.09638700 -3.41907000 -2.35035600

Pb 0.22417400 -1.73743800 -0.24450600

**4**

PBE0-D3(BJ)/def2-SVP∼ma-TZVP

E = -5373.891219 a.u.

C 2.61428200 0.39152700 0.80653100

C 3.87907800 0.98619900 0.83169500

C -3.86492700 0.99649700 0.62169400

C -3.74939800 -0.33248800 0.97983700

C 0.73149200 1.82329900 0.90467800

H 1.29535200 2.57375100 0.32944700

H 0.74365300 2.12801000 1.96890800

C -0.69994300 1.81227500 0.39164500

H -1.25486000 2.64351500 0.85854500

H -0.69449800 1.96415600 -0.70277100

C 3.84521900 3.38841200 0.93646900

H 2.91209100 3.12411900 1.45155600

C 4.56039600 4.39723200 1.82527400

H 4.77697300 3.96665000 2.81389800

H 3.92477300 5.28378900 1.96695700

H 5.50724500 4.74604200 1.38451700

C 3.49222300 3.95233800 -0.43610800

H 4.39381500 4.28085100 -0.97757100

H 2.83297300 4.82829300 -0.33802000

H 2.98281900 3.19500100 -1.05133700

C 5.95280600 2.18514600 0.29107200

H 6.10462600 3.24380700 0.03160300

C 7.00414200 1.84228000 1.34135100

H 6.86096400 0.83462600 1.75637200

H 6.95193100 2.54906800 2.18179600

H 8.01632200 1.89133600 0.91177800

C 6.08300900 1.41037200 -1.01575600

H 7.07402800 1.58068600 -1.46195000

H 5.31356200 1.73264400 -1.73176300

H 5.96035200 0.32771400 -0.88094200

C 5.82701800 -1.68547900 0.91315700

H 6.19829300 -0.65749700 0.83419700

C 6.21840300 -2.20417600 2.29283100

H 5.87938900 -3.23985300 2.44967300

H 5.77921700 -1.57794800 3.08365900

H 7.31267600 -2.19248100 2.41067800

C 6.46603400 -2.48199600 -0.21726700

H 7.56226900 -2.45257900 -0.12723300

H 6.18633600 -2.06705800 -1.19657000

H 6.16221400 -3.53958800 -0.19867300

C -5.75011200 -1.67188700 1.33783200

H -6.15732100 -0.67288800 1.14284900

C -6.26397100 -2.59395900 0.23881300

H -5.91700800 -3.62901800 0.37989000

H -5.92371400 -2.25271200 -0.74993400

H -7.36441400 -2.61061600 0.24054100

C -6.22907100 -2.08755100 2.72422300

H -5.87012600 -3.09139500 2.99838300

H -7.32872600 -2.11138100 2.75640300

H -5.87482900 -1.37975200 3.48831900

C -3.44204900 -2.68937900 1.48992800

H -4.12902500 -3.47915900 1.83199400

C -2.43047100 -2.46048000 2.60403900

H -1.86685700 -3.38493300 2.79671500

H -2.93955800 -2.15849700 3.53200200

H -1.69154600 -1.69235900 2.32998700

C -2.82671200 -3.14644800 0.17346000

H -2.13820700 -3.98849700 0.33013900

H -2.24695900 -2.34522100 -0.30831200

H -3.60383300 -3.46047900 -0.53778400

C 0.02430700 -0.73794700 -4.10370600

C -0.05204200 -2.50755600 -2.18529400

C 1.35519800 0.25725000 -2.12336700

Fe -0.04976900 -0.75113500 -2.35083200

Ge -0.00343600 -0.97745500 0.17257400

N 1.31229100 0.49842000 0.77482900

N -1.29253000 0.52991700 0.70812400

N 4.59252700 2.12951500 0.83816800

C 3.78190200 -0.38821400 0.80222200

N 4.37754600 -1.58430100 0.76039900

C -2.59749900 0.42747300 0.76185000

N -4.29955000 -1.50938500 1.29771300

O 0.07359600 -0.71863000 -5.25680300

O -0.07393300 -3.66322600 -2.07464800

O 2.28229300 0.96457700 -2.02467400

C 3.58621800 -2.82516800 0.75870200

H 4.33035800 -3.62500600 0.89466800

C 2.91582700 -3.03978200 -0.58692600

H 2.29035000 -3.94311400 -0.58070000

H 3.66665300 -3.14041300 -1.38445600

H 2.26283200 -2.19806100 -0.86272200

C 2.63860800 -2.89276900 1.94822400

H 2.14796900 -3.87674300 1.97978100

H 1.83412000 -2.14471800 1.87241800

H 3.18450400 -2.74339500 2.89224900

O -2.60863500 0.61071300 -2.12624400

C -1.58754400 0.04486600 -2.19407200

N -4.55726000 2.09044300 0.27700000

C -3.83480200 3.19227200 -0.37174300

C -6.00382300 2.19553800 0.45264600

H -2.82124900 2.80927200 -0.53478700

C -4.39142100 3.51184500 -1.75255900

C -3.75709800 4.41273900 0.53620300

H -6.23941300 3.24694200 0.22637000

C -6.40520300 1.96953800 1.90586300

C -6.77362100 1.34261800 -0.55229100

H -5.42079700 3.90192300 -1.71157100

H -4.36488300 2.61551500 -2.38723600

H -3.76923900 4.28300500 -2.23039600

H -4.75414700 4.84015800 0.73053800

H -3.14740400 5.20165000 0.07067500

H -3.30395400 4.15299000 1.50500900

H -7.49259400 2.08025100 2.03012900

H -5.90524500 2.70117500 2.55723600

H -6.12728200 0.96771100 2.26344300

H -7.85844500 1.42884300 -0.38830300

H -6.50218800 0.28047600 -0.48332500

H -6.55286200 1.66313200 -1.57926000

**7**

PBE0-D3(BJ)/def2-SVP∼ma-TZVP

E = -7090.030545 a.u.

C 2.52371800 -0.26063200 -1.22092400

C 3.72045000 0.42907100 -1.33381600

C 3.66536400 -0.76135300 -0.60836600

C -2.59782100 0.32495700 -1.05223400

C -3.92607200 -0.03690600 -1.28229800

C -3.61501900 0.85801800 -0.26621100

C 0.64745800 0.18816800 -2.63982900

H 1.11978400 -0.26537000 -3.52752300

H 0.76101700 1.28510900 -2.70887200

C -0.82856400 -0.16212700 -2.60553900

H -1.36391900 0.39776900 -3.38941300

H -0.95164700 -1.24270000 -2.79822000

C 3.51315600 2.62296900 -2.23064400

H 2.49363400 2.26431600 -2.03969700

C 3.66179000 3.88739500 -1.39707300

H 2.88139200 4.60172900 -1.69676500

H 4.63617400 4.37817500 -1.53662700

H 3.51747600 3.66934100 -0.32943200

C 3.61388900 2.87569600 -3.72991900

H 2.78937600 3.53419300 -4.04221700

H 3.52564400 1.93302700 -4.29015200

H 4.55476600 3.36328600 -4.01924000

C 5.80690700 1.59785200 -1.48964100

H 6.14938700 0.55040600 -1.52239600

C 6.10152900 2.13251200 -0.08932300

H 7.15285400 1.93807500 0.17321600

H 5.46065400 1.64910900 0.66257600

H 5.93618300 3.21633800 -0.02229300

C 6.58791100 2.33332200 -2.56704900

H 7.66259400 2.25107300 -2.34807300

H 6.34348500 3.40450700 -2.59870800

H 6.40794200 1.90270600 -3.56196500

C 5.62081400 -1.57268200 0.53635800

H 5.92053500 -0.60845900 0.10375500

C 5.80700900 -1.43423800 2.04415400

H 6.81696300 -1.04634500 2.24808600

H 5.71004900 -2.39296400 2.56920600

H 5.07371300 -0.72993200 2.45806500

C 6.52494200 -2.63618300 -0.07412600

H 7.57485300 -2.40761200 0.16328700

H 6.41924700 -2.67301700 -1.16843200

H 6.30894500 -3.63713700 0.32590900

C 3.39051300 -2.93334400 0.27975300

H 2.35436400 -2.56118800 0.32056300

C 3.61606200 -3.68424600 1.57851600

H 2.85097200 -4.47104700 1.64979600

H 3.49349000 -3.02907300 2.44969500

H 4.59941300 -4.17545800 1.62433800

C 3.49220800 -3.83715600 -0.94349000

H 2.72256700 -4.62047600 -0.88634200

H 4.47487800 -4.32558600 -1.02170500

H 3.30668800 -3.26754000 -1.86641500

C -4.46803500 -1.94938100 -2.68976600

H -5.41121700 -2.49654400 -2.84068000

C -3.91791600 -1.59461200 -4.06415700

H -3.69551300 -2.50768000 -4.63608300

H -2.98589200 -1.01988200 -3.97945000

H -4.63966200 -0.99437600 -4.63678800

C -3.54288200 -2.84747700 -1.88308400

H -3.32130300 -3.76951600 -2.43926900

H -3.99958000 -3.12081100 -0.92145100

H -2.58409900 -2.36171400 -1.65771400

C -6.26538100 -0.49687900 -1.65015800

H -6.29299800 0.49903500 -1.18299400

C -7.09310600 -0.40808800 -2.92510400

H -8.12817700 -0.12882100 -2.67900400

H -7.13308700 -1.36902200 -3.46039200

H -6.68446300 0.35117900 -3.60752300

C -6.81887500 -1.51033900 -0.65445700

H -7.84684900 -1.24739600 -0.36281800

H -6.19606600 -1.54860600 0.25214100

H -6.84535000 -2.52307700 -1.08595900

C -5.34154700 1.50242300 1.27250800

H -5.74327700 0.63993900 0.72417000

C -6.25471000 2.68720400 0.98444400

H -7.27916800 2.45591700 1.31340800

H -6.28235400 2.91953200 -0.09099100

H -5.93436400 3.59242100 1.51961800

C -5.34502300 1.08584600 2.74048700

H -6.34379800 0.70580500 3.00519600

H -5.11854100 1.91843900 3.41810500

H -4.61337300 0.28365700 2.90856800

C -3.07406100 2.79084000 0.99667500

H -2.06472200 2.36673700 0.87236300

C -3.22770600 3.89675100 -0.04233700

H -2.41428500 4.62818800 0.07386700

H -4.18488200 4.43127000 0.05559300

H -3.15654400 3.48903200 -1.06217700

C -3.13266000 3.32616600 2.41498000

H -2.31889400 4.05622600 2.53454600

H -2.97279700 2.53299900 3.15477500

H -4.07586200 3.84681700 2.63795400

C -0.74815100 -3.97005300 1.66521200

C 0.66742200 -1.91987700 2.29697200

C -0.12894500 -2.91979000 -0.57247600

C -2.14045300 -1.79855600 1.36307700

C 0.79953200 3.28317300 2.21346500

C -0.51539900 1.11291700 2.54895800

C 0.33348700 2.55608700 -0.16797700

C 2.25168900 1.17354500 1.66551200

N 1.21722800 -0.36707500 -1.42761700

N -1.30428600 0.22541300 -1.29155200

N 4.36593400 1.50730300 -1.77071900

N 4.21663900 -1.71396800 0.12648600

N -4.84825300 -0.74463700 -1.93348200

N -3.99045400 1.66398000 0.71958200

O -0.90445000 -5.03933100 2.06180800

O 1.42104500 -1.66885600 3.13558600

O 0.09795700 -3.33859900 -1.63061800

O -3.24270800 -1.50922800 1.58043100

O 0.89911500 4.29255900 2.75955800

O -1.24566100 0.71869900 3.35185000

O 0.16080200 3.12698800 -1.16642500

O 3.34529900 0.88180100 1.91628600

Fe -0.50264700 -2.34225100 1.04355200

Fe 0.62414800 1.75494400 1.36303000

Ge 0.01170700 -0.20438500 0.14608500

**5**

PBE0-D3(BJ)/def2-SVP∼ma-TZVP

E = -3511.478418 a.u.

C 4.21109400 1.16757700 0.03024000

C 2.93790400 0.57900000 0.13443200

C 4.10883100 -0.19453300 0.22116700

C -2.37485300 1.27463700 0.24265300

C -3.46706200 1.15881400 -0.61547700

C -3.65318000 1.11217200 0.76229800

C 1.14906200 2.04264100 -0.18960200

H 1.72672900 2.49157200 -1.01571400

H 1.24100900 2.70765600 0.69359900

C -0.30305300 1.99381100 -0.59837400

H -0.64589600 3.02647000 -0.79959100

H -0.37156600 1.43148200 -1.54472800

C 4.22937100 3.58422300 -0.10888800

H 3.23342700 3.36373500 0.29422800

C 4.88436300 4.57286000 0.84812000

H 4.27753300 5.48818700 0.91173700

H 5.89016700 4.87283600 0.51470900

H 4.96634200 4.14599000 1.85826400

C 4.05442200 4.15976700 -1.50988200

H 3.44474000 5.07533400 -1.47977100

H 3.55468600 3.43641200 -2.17134200

H 5.02300700 4.42414000 -1.96363200

C 6.34432900 2.28145000 -0.50119400

H 6.56156600 3.30932300 -0.83039400

C 7.24760200 2.00929500 0.69897300

H 7.13019500 2.79757000 1.45564900

H 8.30496400 1.97993300 0.39522700

H 7.00832500 1.05480200 1.18813300

C 6.60785800 1.38655500 -1.70642500

H 6.00845800 1.71958700 -2.56615400

H 6.34873000 0.33712400 -1.51321900

H 7.66999300 1.42345000 -1.99007900

C 6.12808300 -1.54024400 0.44778400

H 6.52902900 -0.52717100 0.32787000

C 6.54837600 -2.03616500 1.82671300

H 6.18475200 -3.05738600 2.01822300

H 6.15282200 -1.37984500 2.61589400

H 7.64578500 -2.05453300 1.90855100

C 6.70727100 -2.39088400 -0.67726600

H 6.40080000 -2.00592500 -1.66098500

H 6.38120700 -3.43965500 -0.60943600

H 7.80656500 -2.38454300 -0.62951300

C -2.89664800 0.99768400 -2.94910600

H -1.93708500 0.87234600 -2.43465000

C -2.84869300 2.28906100 -3.75429300

H -2.04580700 2.24085200 -4.50497000

H -2.65677100 3.15268100 -3.09970700

H -3.79421600 2.46894900 -4.29163400

C -3.07829600 -0.23741300 -3.81787400

H -2.23582500 -0.31050000 -4.52050300

H -4.00594200 -0.20500400 -4.41090400

H -3.07152200 -1.14644900 -3.20149000

C -5.31796500 1.00828200 -2.21214500

H -5.35511600 1.11170900 -3.30742500

C -6.08749900 2.19217400 -1.64003700

H -7.14586800 2.14392300 -1.93601200

H -5.66646400 3.13806000 -2.01151000

H -6.04905200 2.21980700 -0.54240600

C -5.90677400 -0.35550300 -1.86268900

H -5.43576100 -1.14413400 -2.46485600

H -6.99082900 -0.37605100 -2.05088300

H -5.72588800 -0.62061200 -0.81180800

C -5.84746700 0.70002000 1.75051100

H -6.03829900 0.56238500 0.67927600

C -6.70916000 1.86295000 2.23061000

H -7.77346100 1.64668200 2.05369700

H -6.45239100 2.79241700 1.70060500

H -6.58650200 2.04366700 3.30981700

C -6.19939800 -0.61162000 2.44059800

H -5.57548200 -1.42715600 2.05128000

H -7.25660600 -0.85695600 2.25820900

H -6.06093000 -0.54811900 3.53120300

C -3.81111600 1.18241300 3.17154400

H -4.66274500 1.27747300 3.86333600

C -3.00006500 2.46940500 3.24786500

H -3.61288000 3.33789600 2.96259600

H -2.12188500 2.42245400 2.58412500

H -2.63765100 2.62417900 4.27502500

C -2.99820600 -0.03421400 3.59033100

H -2.08793200 -0.13358000 2.97805900

H -3.57429900 -0.96298700 3.48829700

H -2.67106600 0.06644900 4.63614600

C -1.66061600 -3.23979000 -1.89721900

C -0.19200400 -3.45025700 0.25477700

C -0.19068800 -1.16982300 -1.76406700

C -2.59569300 -1.90425500 0.11343900

Fe -1.04348900 -2.20197100 -0.63335600

N 1.63956100 0.70777500 0.11658400

N -1.07880800 1.40198300 0.47509400

N 4.92829800 2.29654600 -0.13351100

N 4.67972200 -1.39771400 0.33540800

N -3.89651500 1.08336600 -1.87464800

N -4.42241100 1.01589500 1.84516400

O -2.07363000 -3.91577800 -2.73882300

O 0.32771900 -4.32982200 0.80911800

O 0.35729300 -0.56440600 -2.60017000

O -3.66488700 -1.82690800 0.57795300

Sn -0.02733600 -0.54952700 1.16984000

C 3.83764600 -2.60163600 0.41256000

H 4.54894800 -3.44154100 0.43167700

C 3.04894900 -2.64423700 1.71150000

H 2.42554000 -3.54694500 1.75809800

H 2.37279100 -1.77851800 1.80231400

H 3.72104200 -2.62631200 2.58198000

C 2.98788500 -2.74749100 -0.83858900

H 2.41372800 -3.68260300 -0.80686600

H 3.62337600 -2.75201700 -1.73672800

H 2.25845300 -1.93135300 -0.93684600

**8**

PBE0-D3(BJ)/def2-SVP∼ma-TZVP

E = -5227.612063 a.u.

Sn 0.00000500 -0.56128100 -0.00024900

Fe -0.31032800 -1.39369100 2.37207500

Fe 0.31009500 -1.39205800 -2.37321800

O 1.64074200 0.63055100 3.14704700

O 0.40541700 -4.07863400 1.46179800

O -0.51312300 -2.36004400 5.10347700

O -3.06638600 -0.44970000 2.36134500

O -1.64148300 0.63227900 -3.14666400

O -0.40755400 -4.07750700 -1.46598300

O 0.51389500 -2.35522600 -5.10567100

O 3.06677800 -0.44986900 -2.36044300

C 1.97155900 -0.82951000 -2.30967200

N -1.37040000 1.15668800 -0.05280700

N 1.37058100 1.15656600 0.05349400

N -4.75511000 2.71687600 0.23353000

N -4.36933200 -0.79872300 -0.79404500

N 4.75535000 2.71670200 -0.23240500

N 4.36939800 -0.79902700 0.79473500

C -2.67185700 1.12296100 -0.18358300

C -3.94786900 1.68731000 -0.08225600

C -3.81572400 0.36891200 -0.46676000

C 2.67202900 1.12274900 0.18429800

C 3.94806700 1.68707900 0.08310900

C 3.81585800 0.36866000 0.46750800

C -0.69200600 2.38171200 0.31026200

H -1.24566500 3.26320600 -0.04479600

H -0.58711700 2.46229500 1.40887600

C 0.69228700 2.38173300 -0.30926100

H 0.58742600 2.46264300 -1.40785200

H 1.24600600 3.26307500 0.04607800

C -4.32182300 3.76921400 1.16078000

H -5.25701600 4.20742200 1.54372600

C -3.56134000 4.88593800 0.45495500

H -3.26530700 5.66657500 1.17188000

H -4.17844700 5.35177300 -0.32550500

H -2.64949400 4.50767700 -0.02658600

C -3.58689300 3.19602100 2.36505000

H -3.37644100 3.99122600 3.09508900

H -2.62920700 2.73620900 2.08846700

H -4.19009700 2.42030100 2.85781800

C -6.16994800 2.65370600 -0.13914400

H -6.22448900 1.88113300 -0.92075300

C -6.64894800 3.95071400 -0.77965300

H -7.68368900 3.83145900 -1.13338600

H -6.01990200 4.22034900 -1.64009500

H -6.64340000 4.79014800 -0.06740000

C -7.05410500 2.22445700 1.02712200

H -8.09323600 2.08088600 0.69425100

H -7.06566400 2.98521900 1.82327700

H -6.69848700 1.28170900 1.46607300

C -5.80124300 -0.96485200 -0.50344200

H -6.21351400 0.05013300 -0.58518000

C -6.54503900 -1.80633100 -1.52822500

H -7.62211800 -1.76370200 -1.30813000

H -6.24728800 -2.86344800 -1.49394100

H -6.38753000 -1.43243600 -2.54933900

C -6.05970900 -1.43132400 0.92822200

H -7.12406800 -1.30000100 1.17773600

H -5.45545900 -0.85359500 1.64176800

H -5.81276500 -2.49234800 1.06595000

C -3.47307300 -1.90222400 -1.18684900

H -2.46428400 -1.47796900 -1.07845200

C -3.59635700 -2.27682400 -2.65915800

H -2.72368400 -2.88096100 -2.94437300

H -3.59660600 -1.37605700 -3.28836900

H -4.49778100 -2.86537900 -2.87505500

C -3.54017900 -3.11041600 -0.26439500

H -2.73429500 -3.80645200 -0.53472200

H -4.49204800 -3.65436400 -0.35639900

H -3.39434900 -2.82137100 0.78506900

C 6.17011400 2.65353700 0.14052400

H 6.22451300 1.88097100 0.92214700

C 7.05450600 2.22429300 -1.02555800

H 8.09358300 2.08082800 -0.69247500

H 7.06617200 2.98500600 -1.82175700

H 6.69905300 1.28149400 -1.46451100

C 6.64895700 3.95056900 0.78110700

H 7.68366300 3.83138000 1.13496600

H 6.01978100 4.22015200 1.64147200

H 6.64343700 4.79001100 0.06886400

C 5.80138100 -0.96508700 0.50445100

H 6.21365700 0.04986600 0.58666000

C 6.06022700 -1.43113900 -0.92728100

H 7.12460700 -1.29957100 -1.17656500

H 5.45599700 -0.85338700 -1.64083000

H 5.81350600 -2.49217900 -1.06528200

C 6.54487600 -1.80693600 1.52915100

H 7.62203100 -1.76412300 1.30946300

H 6.24722100 -2.86405900 1.49429800

H 6.38697700 -1.43348900 2.55036400

C 3.47301400 -1.90266600 1.18685500

H 2.46426600 -1.47830200 1.07851200

C 3.54018400 -3.11043000 0.26384200

H 2.73403800 -3.80640800 0.53354100

H 4.49188500 -3.65465500 0.35597700

H 3.39485600 -2.82087300 -0.78554800

C 3.59595600 -2.27805300 2.65899200

H 2.72292900 -2.88189800 2.94376300

H 3.59651400 -1.37760600 3.28866700

H 4.49706400 -2.86715100 2.87472500

C 0.87085900 -0.16341600 2.80100700

C 0.11430800 -3.00587900 1.77514000

C -0.43475900 -1.98070800 4.02071900

C -1.97146600 -0.83005100 2.30966200

C -0.87132000 -0.16168100 -2.80121000

C -0.11556200 -3.00463200 -1.77806300

C 0.43513600 -1.97721500 -4.02248200

C 4.32230400 3.76897800 -1.15986700

H 5.25760100 4.20713300 -1.54260900

C 3.58764900 3.19569400 -2.36426300

H 3.37777000 3.99077000 -3.09461000

H 2.62968200 2.73631900 -2.08791700

H 4.19076800 2.41962100 -2.85657900

C 3.56168200 4.88582200 -0.45439800

H 3.26634800 5.66660700 -1.17145000

H 4.17842300 5.35141200 0.32649800

H 2.64939400 4.50781700 0.02649600

**6**

PBE0-D3(BJ)/def2-SVP∼ma-TZVP

E = -3490.045650 a.u.

Pb -0.04655000 -0.58534800 -1.18549000

N -4.46858100 1.16926300 -1.68566100

N 4.99530000 2.36282900 0.19982300

N 4.72806400 -1.34969100 -0.18607000

O -2.23213500 -3.95797500 2.71568700

O 0.27829800 -0.63131200 2.66318600

O -3.70051800 -1.75803800 -0.58539700

O 0.33702100 -4.34752800 -0.73345300

Fe -1.11191700 -2.23889600 0.66343000

C 3.00308400 0.64201600 -0.05560000

C 4.27875500 1.23079700 0.05252800

C 4.17573500 -0.13329100 -0.10768300

C -0.26326900 2.02404400 0.65168600

C 1.18415000 2.07949600 0.21949400

C -2.81932200 1.01535000 3.07181200

C -3.66978200 1.21784000 -0.61773800

C -2.37158500 1.36448000 -0.13843800

C -3.43973700 1.21538400 0.75083500

C -2.77366000 2.29553300 3.89552100

C -2.97024100 -0.23356100 3.92682100

C -5.25280100 1.00470000 2.38133300

C -6.04513000 2.19760400 1.86102100

C -5.83460400 -0.35392400 2.00127300

C -5.88744600 0.83283000 -1.57955700

C -6.77152600 2.00769600 -1.98447400

C -6.23761500 -0.44570400 -2.33082700

C -3.88744800 1.41519600 -3.01113200

C -3.14055800 2.74227900 -3.05018100

C -3.01807600 0.25779400 -3.48195600

C 4.29461700 3.64820900 0.14781500

C 4.94602400 4.61515900 -0.83355000

C 4.12224100 4.25487500 1.53585400

C 6.41106800 2.35049100 0.56514400

C 7.30675600 2.02666500 -0.62767200

C 6.67121000 1.49254700 1.79784800

C 6.17391600 -1.52971900 -0.25932600

C 6.61375300 -2.07762200 -1.61236500

C 6.70410500 -2.35997400 0.90447900

C 3.85146700 -2.52796300 -0.25547600

C 3.08408700 -2.56511600 -1.56848000

C 2.96924600 -2.62175100 0.97859200

C -1.78251900 -3.27906100 1.89559800

C -0.26424500 -1.22435600 1.81624300

C -2.64258300 -1.88408800 -0.10552300

C -0.22052600 -3.47785000 -0.19727400

N -1.09290400 1.51322200 -0.42325400

N 1.70693200 0.75139500 -0.05549800

N -3.83966200 1.10302700 2.01856600

H -0.57895300 3.05020800 0.92320800

H -0.31409100 1.41236400 1.56900300

H 1.75921800 2.56372700 1.02882600

H 1.25262200 2.72883700 -0.67790600

H -1.86716700 0.91272100 2.53878400

H -2.60432300 3.17102800 3.25052300

H -3.71219300 2.45489700 4.45135900

H -1.95754100 2.24712600 4.63185600

H -2.96939800 -1.13376100 3.29747600

H -2.11318900 -0.30892500 4.61113400

H -3.88619400 -0.21829300 4.53848400

H -5.27002800 1.07395600 3.47973500

H -6.02425900 2.26160200 0.76459800

H -7.09802200 2.12768700 2.17205000

H -5.62839100 3.13565500 2.25646400

H -5.67414900 -0.58289100 0.93848500

H -5.34100500 -1.15554000 2.56741000

H -6.91397300 -0.39389000 2.21218500

H -6.05604600 0.63773000 -0.51335600

H -7.83016300 1.76969000 -1.80136300

H -6.51653700 2.91230800 -1.41236000

H -6.66933500 2.24456700 -3.05500900

H -6.11453400 -0.32533400 -3.41866500

H -5.60148300 -1.27388700 -1.99171800

H -7.29004700 -0.71060300 -2.14813900

H -4.75104000 1.48902400 -3.69019900

H -2.79886300 2.95396900 -4.07451200

H -3.79017200 3.56851700 -2.72434600

H -2.25330900 2.70920200 -2.39746400

H -2.72317000 0.39922300 -4.53257300

H -2.09028400 0.20696200 -2.89181700

H -3.53675600 -0.70503000 -3.38459800

H 3.29718500 3.41646200 -0.24631300

H 4.33948300 5.52914700 -0.91650500

H 5.02519600 4.16497100 -1.83379000

H 5.95313700 4.92193000 -0.51023100

H 5.09207800 4.52445800 1.98392600

H 3.51638400 5.17213000 1.48617100

H 3.61897100 3.54759100 2.21186900

H 6.63813500 3.38724200 0.85797500

H 8.36563800 2.00110800 -0.32890400

H 7.05720400 1.05510000 -1.07688800

H 7.19006200 2.78575900 -1.41384600

H 7.73443500 1.52902400 2.07760800

H 6.07682500 1.85696900 2.64827000

H 6.40230000 0.43982300 1.63719700

H 6.59708100 -0.52295000 -0.16000400

H 7.71185300 -2.12656400 -1.66781800

H 6.22822900 -3.09476500 -1.78205600

H 6.25382900 -1.43558800 -2.42990100

H 7.80380500 -2.38990400 0.88061000

H 6.38956600 -1.93306200 1.86816000

H 6.34555700 -3.39962100 0.86341700

H 4.53266400 -3.39268500 -0.24690200

H 3.77005400 -2.61375400 -2.42690100

H 2.40715500 -3.42943000 -1.60132400

H 2.47467800 -1.65582800 -1.69441300

H 2.35796100 -3.53313300 0.94830800

H 3.58463900 -2.63816900 1.89043100

H 2.27496900 -1.77276100 1.04993400

**9**

PBE0-D3(BJ)/def2-SVP∼ma-TZVP

E = -5206.153401 a.u.

C -2.67790800 -0.47595700 -1.16625100

C -3.92943400 -0.07242700 -1.65678200

N -4.70396700 0.63318700 -2.49508700

C -3.86569800 -0.94121300 -0.58515000

N -1.36698500 -0.47172600 -1.19776400

C -0.74350900 0.07617600 -2.38817400

H -0.89317900 1.16827000 -2.47354300

H -1.17143700 -0.39366700 -3.29147400

C -4.20769500 1.85503300 -3.14546400

H -5.11571500 2.39188500 -3.46090000

C -3.40685500 1.54966900 -4.40393000

H -3.11387800 2.48313700 -4.90742400

H -3.99713500 0.94693200 -5.10913300

H -2.48951000 0.99631800 -4.16628200

C -3.47376600 2.75325200 -2.16174500

H -2.57227700 2.27323800 -1.76091300

H -4.11642800 3.00541300 -1.30602500

H -3.15887200 3.68700800 -2.64905200

C -6.14645000 0.37986400 -2.50078600

H -6.26638500 -0.60748000 -2.03192900

C -6.69450000 0.25944900 -3.91702400

H -6.62368000 1.20908200 -4.46927000

H -7.75776400 -0.02130800 -3.88549100

H -6.15043600 -0.51023900 -4.48331900

C -6.90741400 1.40462300 -1.66464500

H -6.48122500 1.48202700 -0.65288200

H -7.96802200 1.12452900 -1.57616200

H -6.86760500 2.40573800 -2.12125600

C -5.94131400 -1.48710800 0.54057000

H -6.20137100 -0.58184600 -0.02226000

C -6.74669300 -2.65254300 -0.02249800

H -6.52106000 -2.81122400 -1.08813600

H -7.82602400 -2.46359900 0.07952000

H -6.52333600 -3.58962300 0.51099200

C -6.27050500 -1.19723100 1.99935400

H -6.05727600 -2.05853600 2.65058000

H -7.34269700 -0.97039700 2.09733400

H -5.69669800 -0.33207000 2.35783400

C -2.75158400 1.56785200 1.43672900

C -0.24709300 1.93625000 2.98304800

C -1.61651500 3.82368700 1.99656400

C -0.54219900 2.88191500 -0.07227500

Fe -1.12107600 2.23243800 1.46324500

N -4.50625300 -1.67695000 0.32005000

O -3.86520300 1.24899000 1.45374500

O 0.29510600 1.77857900 3.98855500

O -1.94589200 4.87144500 2.33817100

O -0.22773800 3.36273600 -1.07798200

Pb 0.00005800 0.01864600 0.68277700

C -3.78329500 -2.68595100 1.10597500

H -4.56897000 -3.30775900 1.56376500

C -2.95068600 -3.58043400 0.20192200

H -2.17818100 -3.00863300 -0.32988900

H -3.58521400 -4.08149600 -0.54460200

H -2.43424100 -4.34890100 0.79450000

C -2.97323300 -2.05144500 2.22247900

H -2.19990000 -1.39092200 1.80903300

H -2.45687100 -2.81736200 2.81590800

H -3.60031000 -1.44854900 2.89150900

C 2.67780700 0.41428500 -1.19062600

C 3.92913200 -0.01570200 -1.65886300

N 4.70343800 -0.76560500 -2.45810600

C 3.86594700 0.90900600 -0.63507200

N 1.36689900 0.40904600 -1.22177300

C 0.74313600 -0.20236600 -2.38063600

H 0.89261100 -1.29747200 -2.40665500

H 1.17116300 0.21775700 -3.30805900

C 4.20699400 -2.02064500 -3.04187400

H 5.11499900 -2.57403800 -3.32735100

C 3.40704700 -1.78350300 -4.31552200

H 3.11418800 -2.74280000 -4.76795000

H 3.99800000 -1.21996300 -5.05189300

H 2.48964900 -1.21794400 -4.10887600

C 3.47195000 -2.86389600 -2.01154900

H 2.57143800 -2.36139700 -1.63696900

H 4.11422300 -3.07108200 -1.14355200

H 3.15526600 -3.82177200 -2.44822500

C 6.14609700 -0.51382900 -2.47663100

H 6.26643500 0.49736600 -2.06181200

C 6.69492700 -0.47083000 -3.89703500

H 6.62383300 -1.44907500 -4.39678600

H 7.75833800 -0.18933500 -3.88030500

H 6.15151000 0.26717200 -4.50461700

C 6.90593800 -1.49234900 -1.58590800

H 6.47892600 -1.51504100 -0.57180300

H 7.96658900 -1.20830700 -1.51183300

H 6.86603300 -2.51658800 -1.98794200

C 5.94208600 1.51154400 0.46049600

H 6.20127500 0.57825500 -0.05487400

C 6.74933600 2.64539400 -0.16139600

H 6.52393100 2.74984400 -1.23375500

H 7.82836300 2.46009500 -0.04986600

H 6.52769100 3.60888500 0.32355800

C 6.27038500 1.29667600 1.93241400

H 6.05814900 2.19061100 2.53841100

H 7.34227700 1.07387000 2.04229100

H 5.69549300 0.45178700 2.33484300

N 4.50730300 1.69134900 0.22963100

C 3.78566800 2.74145500 0.96089400

H 4.57225300 3.38408700 1.38723200

C 2.95695700 3.59051000 0.01056600

H 2.18231600 2.99485400 -0.49116400

H 3.59391200 4.04936100 -0.76063200

H 2.44353800 4.39104400 0.56189400

C 2.97233900 2.16776900 2.10747600

H 2.19625800 1.49064500 1.72736800

H 2.45884600 2.96491700 2.66095200

H 3.59663000 1.59696800 2.80661000

C 2.75163000 -1.49033800 1.51876700

C 0.24593600 -1.77327500 3.08120300

C 1.61303500 -3.71196200 2.19781300

C 0.54329400 -2.88056900 0.08011500

Fe 1.12039200 -2.15079500 1.57976000

O 3.86573500 -1.17273000 1.51918200

O -0.29615000 -1.56126700 4.07674400

O 1.94068700 -4.74019600 2.59575100

O 0.22992400 -3.41311200 -0.89954900

# 3 References

[1] E. Bill, MPI Kohlenforschung, Mühlheim, Germany, **2019**.

[2] E. Bill, MPI Kohlenforschung, Mühlheim, Germany, **2019**.

[3] M. Muhr, P. Heiß, M. Schütz, R. Bühler, C. Gemel, M. H. Linden, H. B. Linden, R. A. Fischer, "Enabling LIFDI-MS measurements of highly air sensitive organometallic compounds: a combined MS/glovebox technique" *Dalton Transactions* **2021**, *50*, 9031-9036.

[4] J. Kouvetakis, A. Haaland, D. J. Shorokhov, H. V. Volden, G. V. Girichev, V. I. Sokolov, P. Matsunaga, "Novel Methods for CVD of Ge4C and (Ge4C)xSiy Diamond-like Semiconductor Heterostructures:  Synthetic Pathways and Structures of Trigermyl-(GeH3)3CH and Tetragermyl-(GeH3)4C Methanes" *J. Am. Chem. Soc.* **1998**, *120*, 6738-6744.

[5] S. Kuppuswamy, J. D. Wofford, C. Joseph, Z.-L. Xie, A. K. Ali, V. M. Lynch, P. A. Lindahl, M. J. Rose, "Structures, Interconversions, and Spectroscopy of Iron Carbonyl Clusters with an Interstitial Carbide: Localized Metal Center Reduction by Overall Cluster Oxidation" *Inorg. Chem.* **2017**, *56*, 5998-6012.

[6] H. Bruns, M. Patil, J. Carreras, A. Vázquez, W. Thiel, R. Goddard, M. Alcarazo, "Synthesis and Coordination Properties of Nitrogen(I)-Based Ligands" *Angew. Chem. Int. Ed.* **2010**, *49*, 3680-3683.

[7] J. C. Dobrowolski, "Three Queries about the HOMA Index" *ACS Omega* **2019**, *4*, 18699-18710.

[8] J. C. Dobrowolski, S. Ostrowski, "HOMA Index Establishes Similarity to a Reference Molecule" *J. Chem. Inf. Model.* **2023**, *63*, 7744-7754.

[9] Bruker, 2015.5-2 ed., Bruker AXS Inc, Madison, Wisconsin, USA, **2015**.

[10] Bruker, Version 8.40A ed., Bruker AXS Inc., Madison, Wisconsin, USA, **2016**.

[11] Bruker, Version 2016/2, ed., Bruker AXS Inc., Madison, Wisconsin, USA, **2016**.

[12] G. M. Sheldrick, *Acta Crystallographica Section C: Crystal Structure Communications* **2015**, *71*, 3-8.

[13] G. M. Sheldrick, *Acta Crystallographica Section A: Foundations of Crystallography* **2015**, *71*, 3-8.

[14] C. B. Huebschle, G. M. Sheldrick, B. Dittrich, "SHELXLE" *J. Appl. Cryst.* **2011**, *44*, 1281.

[15] G. M. Sheldrick, University of Göttingen, Göttingen, Germany, **2014**.

[16] A. J. C. Wilson, V. Geist*, Vol. C*, Kluwer Academic Publishers (published for the International Union of Crystallography), Dordrecht/Boston/London, **1992**, pp. Tables 6.1.1.4 (pp 500-502), 504.502.506.508 (pp. 219-222) and 504.502.504.502 (pp. 193-199).

[17] A. L. Spek, Utrecht University, Utrecht, Netherlands **2010**.

[18] C. F. Macrae, I. J. Bruno, J. A. Chisholm, P. R. Edgington, P. McCabe, E. Pidcock, L. Rodriguez-Monge, R. Taylor, J. van de Streek, P. A. Wood, "Mercury CSD 2.0 - new features for the visualization and investigation of crystal structures" *J. Appl. Crystallogr.* **2008**, *41*, 466-470.

[19] D. Kratzert, V139 ed.

[20] M. J. Frisch, G. W. Trucks, H. B. Schlegel, G. E. Scuseria, M. A. Robb, J. R. Cheeseman, G. Scalmani, V. Barone, G. A. Petersson, H. Nakatsuji, X. Li, M. Caricato, A. V. Marenich, J. Bloino, B. G. Janesko, R. Gomperts, B. Mennucci, H. P. Hratchian, J. V. Ortiz, A. F. Izmaylov, J. L. Sonnenberg, Williams, F. Ding, F. Lipparini, F. Egidi, J. Goings, B. Peng, A. Petrone, T. Henderson, D. Ranasinghe, V. G. Zakrzewski, J. Gao, N. Rega, G. Zheng, W. Liang, M. Hada, M. Ehara, K. Toyota, R. Fukuda, J. Hasegawa, M. Ishida, T. Nakajima, Y. Honda, O. Kitao, H. Nakai, T. Vreven, K. Throssell, J. A. Montgomery Jr., J. E. Peralta, F. Ogliaro, M. J. Bearpark, J. J. Heyd, E. N. Brothers, K. N. Kudin, V. N. Staroverov, T. A. Keith, R. Kobayashi, J. Normand, K. Raghavachari, A. P. Rendell, J. C. Burant, S. S. Iyengar, J. Tomasi, M. Cossi, J. M. Millam, M. Klene, C. Adamo, R. Cammi, J. W. Ochterski, R. L. Martin, K. Morokuma, O. Farkas, J. B. Foresman, D. J. Fox, Wallingford, CT, **2016**.

[21] C. Adamo, V. Barone, "Toward reliable density functional methods without adjustable parameters: The PBE0 model" *The Journal of Chemical Physics* **1999**, *110*, 6158-6170.

[22] S. Grimme, S. Ehrlich, L. Goerigk, "Effect of the damping function in dispersion corrected density functional theory" *J. Comput. Chem.* **2011**, *32*, 1456-1465.

[23] F. Weigend, R. Ahlrichs, "Balanced basis sets of split valence, triple zeta valence and quadruple zeta valence quality for H to Rn: Design and assessment of accuracy" *Phys. Chem. Chem. Phys.* **2005**, *7*, 3297-3305.

[24] J. Zheng, X. Xu, D. G. Truhlar, "Minimally augmented Karlsruhe basis sets" *Theor. Chem. Acc.* **2011**, *128*, 295-305.

[25] E. Papajak, J. Zheng, X. Xu, H. R. Leverentz, D. G. Truhlar, "Perspectives on Basis Sets Beautiful: Seasonal Plantings of Diffuse Basis Functions" *Journal of Chemical Theory and Computation* **2011**, *7*, 3027-3034.

[26] T. Yanai, D. P. Tew, N. C. Handy, "A new hybrid exchange–correlation functional using the Coulomb-attenuating method (CAM-B3LYP)" *Chem. Phys. Lett.* **2004**, *393*, 51-57.

[27] C. Y. Legault, in *CYLview, Vol. 1.0b ed*, Université de Sherbrooke, **2009**.

[28] W. Humphrey, A. Dalke, K. Schulten, "VMD: Visual molecular dynamics" *Journal of Molecular Graphics* **1996**, *14*, 33-38.

[29] K. B. Wiberg, "Application of the pople-santry-segal CNDO method to the cyclopropylcarbinyl and cyclobutyl cation and to bicyclobutane" *Tetrahedron* **1968**, *24*, 1083-1096.

[30] E. D. Glendening, C. R. Landis, F. Weinhold, "NBO 7.0: New vistas in localized and delocalized chemical bonding theory" *J. Comput. Chem.* **2019**, *40*, 2234-2241.

[31] E. D. B. Glendening, J. K.; Reed, A. E.; Carpenter, J. E.; Bohmann, J. A.; Morales, C. M.; Landis, C. R.; Weinhold, Theoretical Chemistry Institute, University of Wisconsin, **2020**.

[32] D. W. Szczepanik, M. Andrzejak, K. Dyduch, E. Żak, M. Makowski, G. Mazur, J. Mrozek, "A uniform approach to the description of multicenter bonding" *Phys. Chem. Chem. Phys.* **2014**, *16*, 20514-20523.

[33] D. W. Szczepanik, M. Andrzejak, J. Dominikowska, B. Pawełek, T. M. Krygowski, H. Szatylowicz, M. Solà, "The electron density of delocalized bonds (EDDB) applied for quantifying aromaticity" *Phys. Chem. Chem. Phys.* **2017**, *19*, 28970-28981.
